# Evolutionary diversity of bile salts in reptiles and mammals, including analysis of ancient human and extinct giant ground sloth coprolites

**DOI:** 10.1186/1471-2148-10-133

**Published:** 2010-05-06

**Authors:** Lee R Hagey, Nicolas Vidal, Alan F Hofmann, Matthew D Krasowski

**Affiliations:** 1 Department of Medicine, University of California - San Diego, La Jolla, CA, USA; 2 Département Systématique et Evolution, Muséum National d'Histoire Naturelle, Paris, France; 3 Department of Pathology, University of Iowa Hospitals and Clinics, Iowa City, IA, USA

## Abstract

**Background:**

Bile salts are the major end-metabolites of cholesterol and are also important in lipid and protein digestion and in influencing the intestinal microflora. We greatly extend prior surveys of bile salt diversity in both reptiles and mammals, including analysis of 8,000 year old human coprolites and coprolites from the extinct Shasta ground sloth (*Nothrotherium shastense*).

**Results:**

While there is significant variation of bile salts across species, bile salt profiles are generally stable within families and often within orders of reptiles and mammals, and do not directly correlate with differences in diet. The variation of bile salts generally accords with current molecular phylogenies of reptiles and mammals, including more recent groupings of squamate reptiles. For mammals, the most unusual finding was that the Paenungulates (elephants, manatees, and the rock hyrax) have a very different bile salt profile from the Rufous sengi and South American aardvark, two other mammals classified with Paenungulates in the cohort Afrotheria in molecular phylogenies. Analyses of the approximately 8,000 year old human coprolites yielded a bile salt profile very similar to that found in modern human feces. Analysis of the Shasta ground sloth coprolites (approximately 12,000 years old) showed the predominant presence of glycine-conjugated bile acids, similar to analyses of bile and feces of living sloths, in addition to a complex mixture of plant sterols and stanols expected from an herbivorous diet.

**Conclusions:**

The bile salt synthetic pathway has become longer and more complex throughout vertebrate evolution, with some bile salt modifications only found within single groups such as marsupials. Analysis of the evolution of bile salt structures in different species provides a potentially rich model system for the evolution of a complex biochemical pathway in vertebrates. Our results also demonstrate the stability of bile salts in coprolites preserved in arid climates, suggesting that bile salt analysis may have utility in selected paleontological research.

## Background

Bile salts are amphipathic, water-soluble end-metabolites of cholesterol that facilitate intestinal absorption of lipids, enhance proteolytic cleavage of dietary proteins, and exert potent antimicrobial activity in the small intestine [1]. The synthesis of bile salts is the major route for elimination of cholesterol (a water-insoluble molecule) from the body [2]. Bile salts are produced by every class of vertebrate animals and show substantial structural diversity across species [1-5].

**Figure 1:**
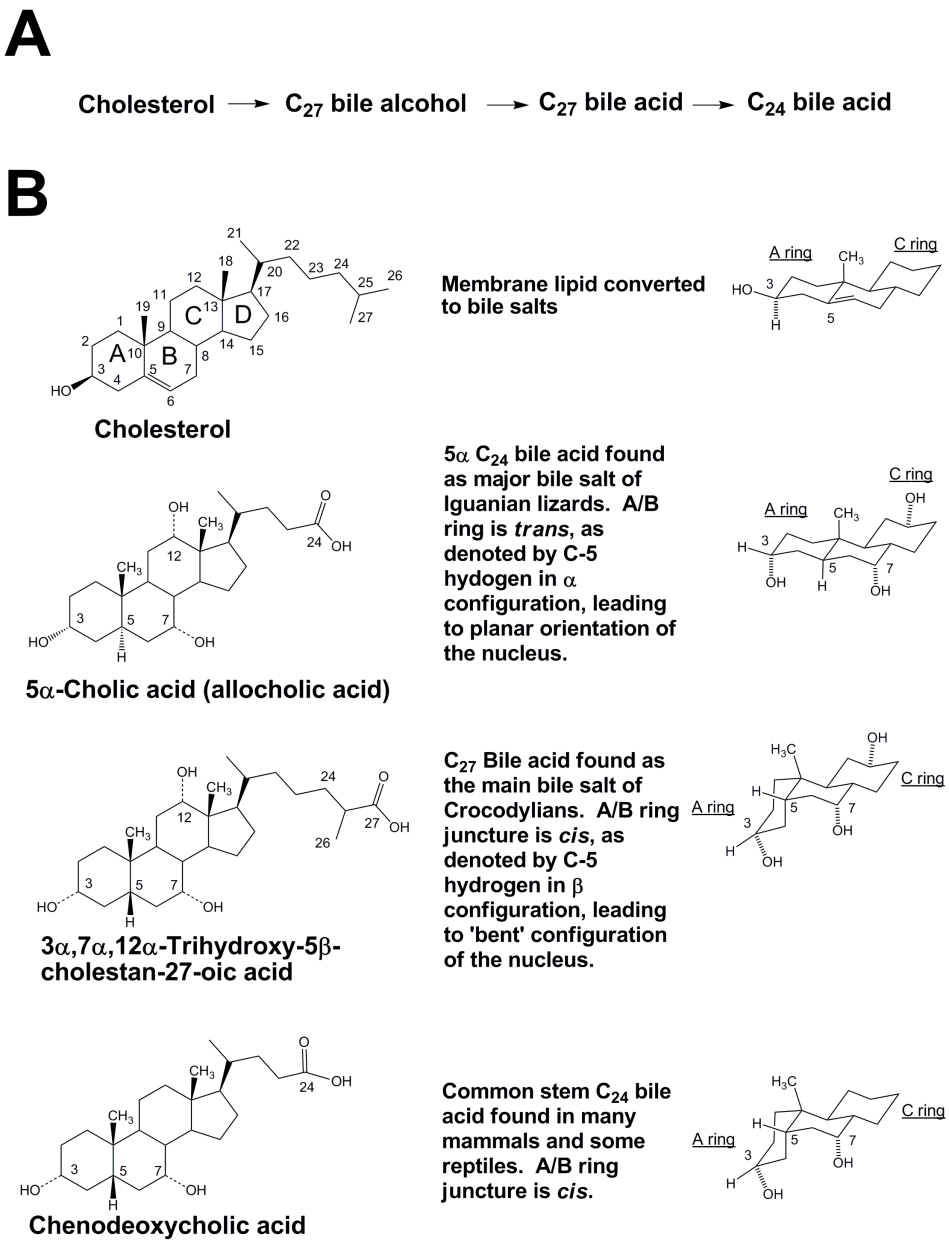
**Representative bile salts and their structures**. (A) Simplified version of the bile salt synthetic pathway showing the three major classes of compounds that can serve as primary bile salts (C_27 _bile alcohols, C_27 _bile acids, and C_24 _bile acids). (B) All bile salts are derived from cholesterol (topmost structure), illustrated with the carbon atoms numbered and the steroid rings labelled A, B, C, and D. Iguanian lizards utilize 5α C_24 _bile acids such as 5α-cholic acid (allocholic acid) that have an A/B ring juncture that is *trans*, resulting in an overall planar and extended structure of the steroid rings (see representation of A, B, and C rings on the right side). Crocodylians utilize trihydroxy 5β C_27 _bile acids with hydroxylation at C-12 in addition to the default 3α, 7α-dihydroxylation. One of the two most common primary salts in mammals is chenodeoxycholic acid (CDCA), the stem C_24 _bile acid that has the default default 3α, 7α-dihydroxylation.

The two basic structural components of bile salts are the 19-carbon (C_19_) steroid nucleus and a side-chain (Figure 1). In all bile salts characterized to date, the four-ring cyclopentanophenanthrene ('steroid') nucleus (with rings labelled A, B, C, and D as in cholesterol in Figure 1B) is fully saturated (i.e., the cholesterol double bond at C5-C6 has been reduced). The A/B ring juncture is variable, being *cis *in most bile salts but *trans *in some species (e.g., jawless fish, lobe-finned fish, agamid lizards), a shift that greatly influences the overall shape of the steroid nucleus. A/B *trans *(5α) bile salts have an extended, planar orientation of the steroid rings, while A/B *cis *(5β) bile salts have a 'bent' orientation of the A ring relative to the other three rings. Virtually all bile salts have a hydroxyl group at C-3 (like cholesterol) and at C-7, because cholesterol 7α-hydroxylase is the rate-limiting enzyme in bile salt biosynthesis [6,7]. Additional common sites of hydroxylation are at C-12 and C-16, but other sites of hydroxylation have been found, as will be described in our study. Hydroxyl groups may also vary in whether they project below (α configuration) or above (β) the plane of the steroid nucleus. In some species, hydroxyl groups have undergone dehydrogenation to an oxo group [1].

Bile salts that retain all the carbon atoms of cholesterol have a total of 27 carbon atoms (C_27_), possessing an 8-carbon (C_8_) side-chain on the D-ring in addition to the C_19 _nucleus. In many species, the side-chain is shortened by three carbon atoms, resulting in a C_5 _side-chain and C_24 _bile acids. Bile salts with total numbers of carbon atoms other than 24 or 27 are uncommon but can occur, usually as minor components of bile [1,5]. Other than length, further structural variation in the side-chain includes the presence and orientation of hydroxyl groups, the presence of unsaturation in the side-chain, and above all, the substituent on the terminal carbon atom, which is a hydroxyl group in bile alcohols and a carboxyl group in bile acids. Side-chain length and the state of oxidation at C-27 is used to assign bile salts to three broad classes (C_27 _bile alcohols, C_27 _bile acids, and C_24 _bile acids), and we use this classification throughout this manuscript. Primary bile salts are those synthesized by the liver, while secondary bile salts result from extra-hepatic modifications of bile salts, typically by host bacteria in the colon and distal small intestine [1,8]. We use the term 'bile salts' to refer to the broad class of cholesterol end-metabolites (bile acids and bile alcohols).

The evolutionarily 'earliest' bile salts are most likely 5α-bile alcohols that have a *trans *A/B junction (resulting in an overall planar structure) and retain all 27 carbon atoms of cholesterol [9,10]. C_27 _5α bile alcohols are the dominant bile salts of jawless and lobe-finned fish [9-13]. Bile alcohols are esterified with sulfate after their biosynthesis and secreted as the ester sulfate into bile. Cholic acid (CA; 3α,7α,12α-trihydroxy-5β-cholan-24-oic acid) and chenodeoxycholic acid (CDCA; 3α,7α-dihydroxy-5β-cholan-24-oic acid) are examples of more evolutionarily recent 24-carbon atom (C_24_) bile acids that have a 'bent' shape because of their *cis *A/B ring juncture (Figure 1B). We propose that bile acids are the derived or apomorphic state for bile salts. Bile acids are typically found in bile *N*-acylamidated ('conjugated') with taurine (or rarely an analog of taurine) or glycine. In prior surveys of bile salts, conjugation of bile acids with taurine was observed to be much more common than with glycine [2,3].

**Figure 2:**
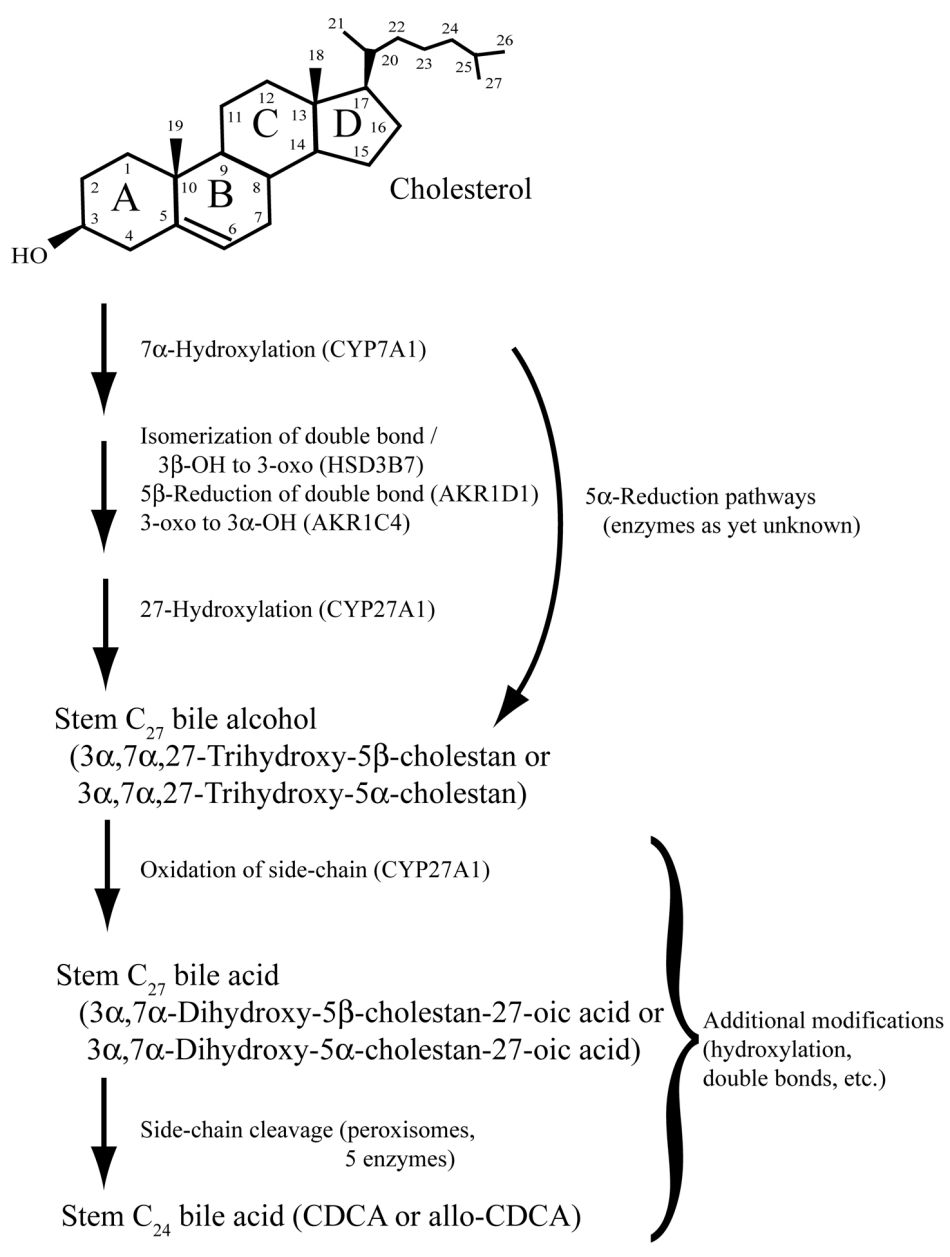
**Bile salt synthetic pathways**. The enzymatic pathways involved in bile salt synthesis have so far been elucidated only in humans and rodents. The pathway shown is the 'neutral' pathway, as opposed to the 'acidic' pathway that modifies the side-chain before the steroid nucleus [6,7]. The ultimate production of the default C_24 _bile acid chenodeoxycholic acid (CDCA) requires multiple changes to the original cholesterol structure including 7α-hydroxylation, isomerization of the C-5 - C-6 double bond (from cholesterol) to form a Δ^4 ^compound coupled with oxidation of the 3β-hydroxy group to a 3-oxo group, stereospecific reduction of the 3-oxo group to a 3α-hydroxy group, hydroxylation of the terminal carbon atom of the side-chain, oxidation of the side-chain (to form a C_27 _bile acid), and shortening of the side-chain. Animals using C_27 _bile alcohols or C_27 _bile acids as their primary bile salts presumably require fewer enzymatic steps than those synthesizing C_24 _bile acids. The enzymes involved in 5α-reduction leading to 5α-bile salts are currently unknown. The stem C_27 _5β-bile alcohol would be 3α,7α,27-trihydroxy-5β-cholestan, while the stem C_27 _5β-bile acid would be 3α,7α-dihydroxy-5β-cholestan-27-oic acid. Note that some steps in the pathway require more than one enzyme (e.g., isomerization of the 3β-hydroxy group of cholesterol to the 3α configuration), whereas some enzymes (e.g., CYP27A1) can catalyze more than one reaction. Double bonds or additional hydroxyl groups may be added to the nucleus or side-chain, either on an intermediate or on the completed stem bile alcohol or acid.

The details of bile salt biosynthetic pathways are known only in humans and rodents, and have not yet been resolved in other species. The synthetic pathway in humans and rodents using C_24 _5β bile acids is long and complex, involving up to 16 enzymes (Figure 2) and requiring the transport of intermediates between multiple organelles [6,7]. Although the details of the bile salt biosynthetic pathways used by non-mammalian species are unknown, animals using exclusively C_27 _bile alcohols likely have simpler and shorter synthetic pathways, as C_27 _bile alcohols do not require side-chain cleavage or oxidation of the terminal alcohol to a carboxylic acid [10,14].

Classification of bile salts into C_27 _bile alcohols, C_27 _bile acids, or C_24 _bile acids places bile salts on a spectrum based on the degree of oxidation (bile alcohol to acid) and alteration of the length of the side-chain, which correlates with the number of steps (and likely number of enzymes) in the bile salt synthetic pathway (Figure 2). C_27 _bile alcohols require only hydroxylation and reduction of the cholesterol double bond. The minimal or 'stem' C_27 _bile alcohol possesses hydroxyl groups at the 3α, 7α, and 27 positions (3α,7α,27-trihydroxy-5α-cholestan or 3α,7α,27-trihydroxy-5β-cholestan) [4]. In humans and rodents, the synthesis of a stem C_27 _bile alcohol structure is catalyzed by five enzymes [7]. C_27 _bile acids additionally require oxidation of the side-chain to a carboxylic acid group, a step which is accomplished in humans and rodents by the multi-functional enzyme cytochrome P450 (CYP) 27A1 that also catalyzes the 25-hydroxylation of 1α-hydroxyvitamin D_2 _and D_3 _[15]. Thus, synthesis of a stem C_27 _bile acid (3α,7α-dihydroxy-5α-cholestan-27-oic acid or 3α,7α-dihydroxy-5β-cholestan-27-oic acid) also only requires five enzymes. C_24 _bile acids require cleavage of the side-chain, a process involving five peroxisomal enzymes in humans and rodents [6,7], in addition to the other steps that are presumed to be shared with the pathways for C_27 _bile alcohols and C_27 _bile acids.

We greatly extend prior surveys of bile salt diversity in reptiles and mammals. The use of multiple analytical techniques enabled the identification of previously unreported bile salts and detailed resolution of biliary bile salt profiles. In this manuscript, we analyze and discuss the patterns of bile salt structural variation across reptiles and mammals, comparing and contrasting our results with current phylogenies of reptiles and mammals. We also discuss the implications for the evolution of the complex bile salt synthetic pathway, with one of the main goals being to provide background data for future studies of the molecular evolution of bile salt enzymes. Lastly, we also present data on analysis of bile salts recovered from human coprolites that are approximately 8,000 years old as well as from coprolites of the Shasta ground sloth (*Nothrotherium shastense*), a mammal that was one of 33 known genera of large (> 50 kg) North American animals that disappeared suddenly at the end of the Pleistocene period approximately 11 000 years ago [16].

## Results

### Overview of bile salt variation in reptiles and mammals

We analyzed the bile salts in biliary bile from 219 reptile species (33 Testudines, tuatara, 66 Squamates, 102 snakes, and 17 Crocodylians) and 326 mammalian species (2 monotremes, 18 marsupials, and 306 placental mammals). We grouped bile salts into three broad types (C_27 _bile alcohols, C_27 _bile acids, and C_24 _bile acids), which allowed us to classify each species into one of six categories based on which one or two bile salt types are present at 10% or greater of the biliary bile salt pool [10]: class I, C_27 _bile alcohols only; class II, C_27 _bile alcohols + C_27 _bile acids; class III, C_27 _bile alcohols + C_24 _bile acids; class IV, C_27 _bile acids only; class V, C_27 _bile acids + C_24 _bile acids; and class VI, C_24 _bile acids only. This classification makes no assumptions about the effectiveness or physiological functions of bile salts.

The bile salt profiles of all species analyzed are in Additional File 1 (reptiles) and Additional File 2 (mammals), with the bile salt class indicated for each species. Unusual bile salt profiles are summarized in Table 1. As noted in prior surveys of vertebrate bile salts [2,3,5], bile salt profiles tend to be conserved within families and even within orders of animals, especially for reptiles and mammals. There were only three reptile families (Chelidae, Testudinae, and Scincidae) and only one mammalian family (Cebidae) that showed different bile salt classes between species within a family, but even in these cases, there was always overlap of bile salts within all species of the family (e.g., some species within a family may have only C_24 _bile acids while other species within the family have both C_24 _and C_27 _bile acids, as seen in Cebidae).

**Table 1:** Unusual bile salt profiles in reptiles and mammals

Bile salt pattern	Examples	Comment
5α ('allo') bile acids	Lizards, especially Iguania	5α bile acids are generally rare in vertebrates

1α-Hydroxylation	Spotted cuscus (*Phalanger maculatus*)	Unusual hydroxylation leads to highly hydrophilic bile acids in this species.

1β-Hydroxylation	Feather-tailed glider (*Acrobates pygmaeus*)	Unusual hydroxylation site

6α-Hydroxylation	Suidae Lesser Malay chevrotain (*Tragulus javanicus*)	Unusual hydroxylation site. Hyocholic acid is only detected in Suidae and the chevrotain.

6β-Hydroxylation	Muridae	Unusual hydroxylation site

7-Oxo bile acids	Queensland koala (*Phascolarctos adjustus*)	Unusual bile salt modification

15α-Hydroxylation	Common wombat (*Vombatus ursinus*)	Not seen in other mammals but found in birds

16α-Hydroxylation	Snakes	Hydroxylation at 16α is common in snakes (Boidae, Cylindrophiidae, Pythonidae, Uropeltidae) and birds but not seen in mammals.

Ursodeoxycholic acid (at > 5%)	Ursidae Caviomorph rodents	Ursodeoxycholic acid is seen in other mammals but only as a small percentage of the bile salt pool.

Δ22 Bile acids	Agouti (*Dasyprocta punctata*) Mountain paca (*Cuniculus taczonowskii*) Pacharana (*Dinomys branickii*) Some snakes	This unusual bile acid side-chain modification is seen in a small number of mammals and is also found in some snakes in Colubridae.

22-Hydroxylated C_27 _bile acids	Turtles	This type of bile salt is unique to turtles.

23*R*-Hydroxylation	Pinnipeds Some snakes	23*R*-Hydroxylation of bile acids is common to Pinnipeds but is not seen in other Carnivora.

100% Bile alcohols	Paenungulates, rhinoceroses	Profile also seen in lobe-finned fish, jawless fish, some teleost fish, and some amphibians.

### Bile salts of reptiles

The bile salts of all reptiles analyzed are summarized in Additional File 1. Two orders of reptiles - Crocodylia and Testudines - showed little variation of bile salt profiles across species. Although species within Crocodylia and Testudines often have biliary bile salt mixtures that are complex and difficult to resolve analytically [17], each order is characterized by a single dominant bile salt (see representative electrospray ionization/tandem mass spectrometry, ESI/MS/MS, data in Additional File 3, Figures S3A-D, M, N). Crocodylian species share the phenotype of using a C_27 _bile acid with a hydroxylation pattern (3α,7α,12α-trihydroxy) common to many bile salts. This C_27 _bile acid (3α,7α,12α-trihydroxy-5β-cholestan-27-oic acid) can be thought of as analogous to CA, the most common C_24 _bile acid. Turtles and tortoises, on the other hand, have C_27 _bile acids with an additional 22-hydroxylation relative to 3α,7α,12α-trihydroxy-5β-cholestan-27-oic acid, a modification to C_27 _bile acids that is so far unique to Testudines. Turtles from the family Emydidae also had a portion of their C_27 _bile acids containing both 15α- and 22*S*-hydroxylation. Testudines also had the only two reptile species in our survey (Siebenrock's snake-neck turtle and California desert tortoise) that had C_27 _bile alcohols (the plesiomorphic state) present at more than 10% of total biliary bile salts.

**Figure 3:**
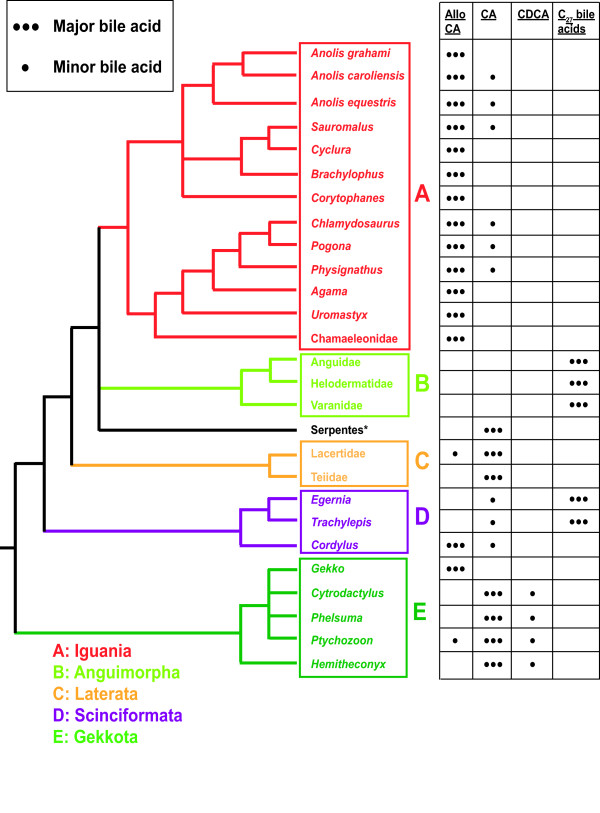
**Bile salts of Squamata**. The bile salt variation of lizards is overlaid on a tree that represents a summary of current knowledge of squamate phylogeny based on molecular data. Groups of squamates are color-coded following the nomenclature in the review by Hedges and Vidal [18]. Major bile salts are those constituting more than 50% of total biliary bile salts. Minor bile salts account for less than 50% but more than 10% of total bile salts. The bile salts of Iguania (group A) are dominated by 5α (allo) bile acids. The C_27 _bile acids of species within Anguimorpha (group B) are almost entirely varanic acid. The bile salts of Laterata (group C) are mainly CA with minor fractions of alloCA in some species. The bile salts of species sampled within Scinciformata showed the most diversity of any of the five squamate groups surveyed, with some species having mainly varanic acid (C_27 _bile acid) and others having mainly alloCA. Species within Gekkota (group E) had mainly CA and CDCA as major bile salts. Abbreviations: alloCA, allo (5α)-cholic acid; CA, cholic acid; CDCA, chenodeoxycholic acid.

The phylogeny of Squamata (lizards, snakes, and amphisbaenians) is still under active debate and study. With the rise of molecular-based phylogenetic studies, the traditional position of many taxa based on morphology and paleontology has been challenged [18-20]. To provide an evolutionary framework, we map the bile salt profiles of Squamata orders onto a tree that represents the summary of current knowledge on Squamata phylogeny, incorporating the species analyzed in our study (Figure 3).

Our survey of Squamata covered species from five groups derived from molecular phylogenetic analyses (Iguania, Anguimorpha, Laterata, Scinciformata, Gekkota; see representative ESI/MS/MS spectra in Additional File 3, Figures S3E-L, Q-T) [18]. All species examined within three families (Agamidae, Chamaeleonidae, Iguanidae) of Iguania had C_24 _5α-bile acids (mainly 5α-CA) as the primary bile salt (see Additional File 3, Figures S3Q, R). 5α Bile acids, also known as 'allo' bile acids, are generally uncommon in vertebrates, being very rare in mammals, fish, and amphibians [9]. Other than Iguanian lizards, the only other vertebrates that commonly use 5α bile acids are waterfowl [2]. Some of the Iguanian species had a minor fraction of 5β bile acids (mainly CA and CDCA). Outside Iguania, the only other reptiles in our sample to predominantly use 5α bile acids were Warren's girdle-tail (*Cordylus warreni*, Cordylidae) and the Tokay gecko (*Gekko gecko*, Gekkonidae). 5α bile acids were not found at greater than 5% of total biliary bile salts in any other reptile species examined.

All species examined within Anguimorpha (including families Anguidae, Helodermatidae, and Varanidae) had a 5β C_27 _bile acid known as varanic acid (3α,7α,12α,24*R*-tetrahydroxy-5β-cholestan-27-oic acid) as the primary bile salt (see representative ESI/MS/MS spectra in Additional File 3, Figures S3E-L), a structure also found in the tuatara (*Sphenodon punctatus*). The expression of this structure in bile as a major primary bile salt is unique to reptiles, but varanic acid is also synthesized (and a subsequently rapidly consumed intermediate) in the longer human bile acid synthetic pathway [6,7]. The species examined with the groups Laterata (including families Lacertidae and Teiidae) and Gekkota had CA as their dominant bile salt. We also examined three species within the group Scinciformata. Two skinks in the family Scincidae - pygmy spiny-tailed skink (*Egernia depressa*) and blotched blue-tailed skink (*Mabuya blandingii*) - had 24*R*-hydroxylated C_27 _bile acids as their major bile acids including varanic acid and 3α,7α,24*R*-trihydroxy-5β-cholestan-27-oic acid (differing from the root C_27 _bile acid by the addition of the 24*R*-hydroxyl group).

**Figure 4:**
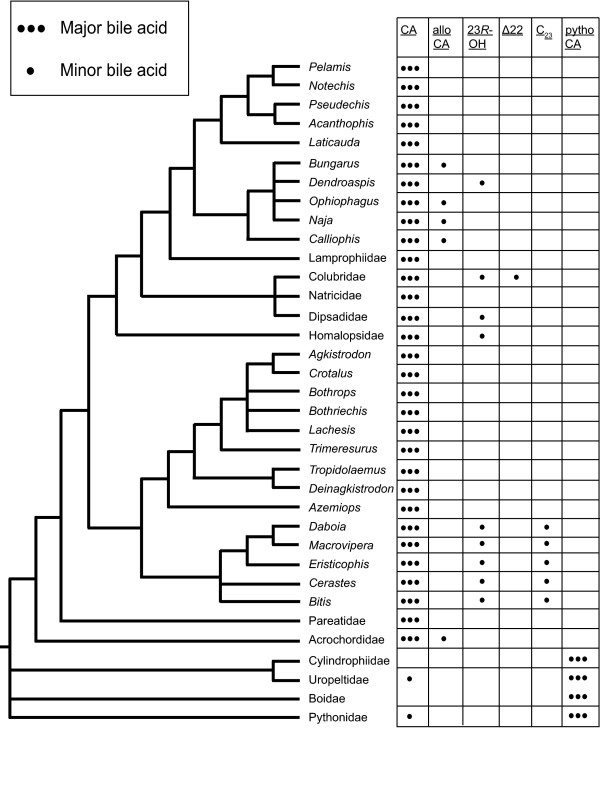
**Bile salts of snakes**. The bile salt variation of snakes is overlaid on a tree that represents a summary of current knowledge of snake phylogeny based on molecular data. Major bile salts are those constituting more than 50% of total biliary bile salts. Minor bile salts account for less than 50% but more than 10% of total bile salts. Abbreviations: CA, cholic acid; 23*R*-OH, 23*R*-hydroxylated C_24 _bile acids (mainly 23*R*-hydroxylated cholic acid); Δ22, C_24 _bile acids with double bond at C22-23; C_23_, C_23 _bile acids; pythoCA, pythocholic acid.

Of the 101 species of snakes examined, the most common major bile salt was the C_24 _bile acid CA (Figure 4). Within the family Viperidae, we examined the bile of 32 species, belonging to two subfamilies, the viperines (true vipers) and the crotalines (pit-vipers) [21]. The viperine clade that includes the genera *Bitis*, *Cerastes*, *Daboia*, *Eristicophis*, and *Macrovipera*, was notable in having the 7-dehydroxylated bile acid known as bitocholic acid (3α,12α,23*R*-trihydroxy-5β-cholan-24-oic acid) as well as C_23 _bile acids in their biliary bile salts. In contrast, the crotaline clade that includes the genera *Agkistrodon*, *Azemiops*, *Bothriechis*, *Bothrops*, *Crotalus*, *Deinagkistrodon*, *Lachesis*, *Trimeresurus*, and *Tropidolaemus *had bile salt profiles consistently of approximately 100% CA. The phylogenetically most basal snake families examined in our survey (Boidae, Cylindrophiidae, Pythonidae, and Uropeltidae) were notable in that all species shared the 7-deoxy bile acid known as pythocholic acid (3α,12α,16α-trihydroxy-5β-cholan-24-oic acid) as a major primary bile acid. Both bitocholic and pythocholic acids are unusual, since in humans and rodents (and presumably the majority of vertebrates) 7-hydroxyation of cholesterol is the first and rate-limiting step of bile acid biosynthesis [6,7]. The mechanism by which these 7-dehydroxylated bile acids are produced in snakes is currently unknown, although it has been suggested that pythocholic acid is formed by 16α-hydroxylation of the secondary bile acid deoxycholic acid (3α,12α-dihydroxy-5β-cholan-24-oic acid; DCA) [22]. This mechanism is physiologically plausible (although not proven) if snakes that feed infrequently shut off new bile acid synthesis between meals and thus have an abundance of 7-deoxy bile acids in their bile, as a result of intestinal bacteria removing the 7-hydroxy group from the original primary bile acids [4]. 7-Deoxy bile acids have not been identified as primary bile acids in any other species [2,9].

### Bile salts of mammals

**Figure 5:**
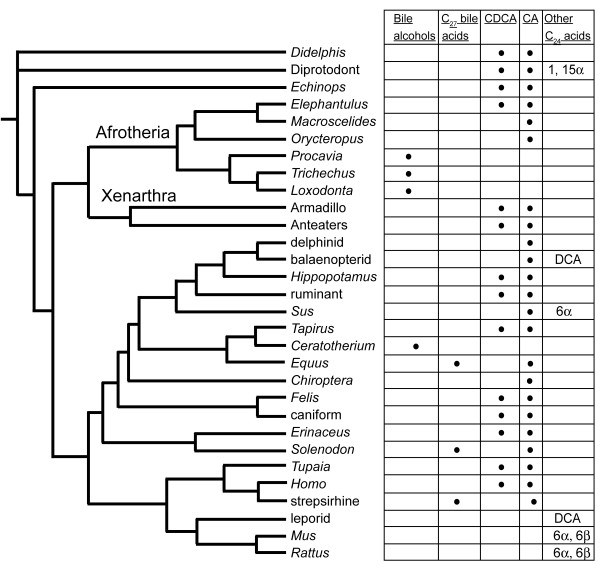
**Bile salts of mammals**. The bile salt variation of mammals is overlaid on a mammalian phylogeny based on molecular data [23], with the single revision to place Afrotheria and Xenarthra as sister groups. In the "Other C_24 _acids" columns are included the 1α-, 1β-, and 15α-hydroxylated bile acids found in some marsupials and the 6α- and 6β-hydroxylated bile acids of rodents and Suidae, as well as deoxycholic acid found in various species. Abbreviations: CDCA, chenodeoxycholic acid; CA, cholic acid; DCA, deoxycholic acid.

We examined the biliary bile salts of 326 mammals, of which 300 had type VI bile salt profiles (>90% C_24 _bile acids) (Additional File 2). We have mapped the bile salt profiles of mammals onto a molecular phylogenetic tree [23], incorporating the species analyzed in our study (Figure 5). Only one mammal analyzed (fox squirrel, *Sciurus niger*) had > 50% 5α bile acids; for most mammals, 5α bile acids were found only at trace levels. Perhaps the most striking finding was that all five species of Paenungulates examined (including two elephants, rock hyrax, and two manatees) had C_27 _bile alcohols as their primary bile salts (Additional File 2) [24,25]. The bile alcohols of Paenungulates utilize 25-hydroxylation, a rare substitution in C_27 _bile salts that is also found in the bile alcohols of lobe-finned fish (coelacanths, lungfishes) [9,11,12,26]. In contrast to the Paenungulates, the bile salts of the South African aardvark (*Orycteropus afer*) and rufous sengi (*Elephantulus rufescens*), two animals also grouped by molecular phylogenetic analyses with Paenungulates in the cohort Afrotheria [27,28], were typical C_24 _bile acids consisting mainly of CA.

The biliary bile salts of 2 species of monotremes (duck-billed platypus and short-nosed echidna) were both composed of greater than 95% C_24 _bile acids. The bile of both monotremes analyzed did, however, contain C_27 _bile acids at about 2% of total bile salts; the C_27 _bile acid present was 3α,7α,12α-trihydroxy-5β-cholestan-27-oic acid, the dominant bile salt of Crocodylians (Additional file 1) and also a primary bile salt of ratite birds (LR Hagey, unpublished data). We examined 18 species of marsupials, which revealed uncommon bile acids relative to placental mammals and other vertebrates: 1α-hydroxy-CDCA (1α,3α,7α-trihydroxy-5β-cholan-24-oic acid) in the spotted cuscus (*Phalanger maculatus*), 1β-hydroxy-CDCA (1β,3α,7α-trihydroxy-5β-cholan-24-oic acid) in the feather-tailed glider (*Acrobates pygmaeus*), 3α-hydroxy-7-oxo-5β-cholan-24-oic acid in the Queensland koala (*Phascolarctos adjustus*), 7-oxo-DCA (3α,12α-dihydroxy-7-oxo-5β-cholan-24-oic acid) in both the kowari (*Dasyuroides byrnei*) and spotted-tailed quoll (*Dasyrus maculatus maculatus*), and 15α-hydroxy bile acids in the common wombat (*Vombatus ursinus*) [29]. Some of these substitutions have also been described in birds, e.g., 1β-bile acids in the tinamou [30], fruit pigeons, and doves [31]; and 15α-hydroxy bile acids in swans, ducks, and geese [32,33].

In placental mammals, the common occurrence of the C_24 _bile acids CA and CDCA limits the phylogenetic information of bile salt variation. However, there are groups of mammals which share less common bile salts. One example is in the pinnipeds, a diverse group of semi-aquatic marine mammals that include the families Odobenidae (walrus), Otariidae (eared seals, including fur seals and sea lions), and Phocidae (earless seals). All were found to have 23*R*-tri- and tetrahydroxy bile acids as their major bile salts. We did not detect 23*R*-hydroxylated bile acids in any other mammals in our survey, including 16 species within Mustelidae, currently proposed as the sister taxa to pinnipeds [34], consistent with other studies suggesting that living pinnipeds comprise a monophyletic group within Carnivora [34,35].

Ursidae are another example of a family sharing an uncommon bile acid. Six of eight species within Ursidae had ursodeoxcholic acid (3α,7β-dihydroxy-5β-cholan-24-oic acid) as a primary bile acid constituting more than 6% of the total biliary bile salts. Ursodeoxycholic acid is unusual in that the orientation of 7-hydroxyl group is 7β, as opposed to the 7α orientation common to most bile salts [36]. Outside Ursidae, ursodeoxycholic acid was not detected at greater than 5% of total biliary bile salts in any other species within Carnivora. The only other mammals in our survey to have more than trace amounts of ursodeoxycholic acid were several rodents, including the North American beaver (*Castor canadensis*, Castoridae) and three caviomorph species within two families (Dasyproctidae, Caviidae) (Additional File 2). Recently published work by other investigators have identified two additional novel bile acids in the Asian black bear (*Selenaretos thibetanus*): 3α,5,7α-trihydroxy-5β-cholan-24-oic acid and 3α,7α,9α-trihydroxy-5β-cholan-24-oic acid [32].

Hydroxylation at C-6 was also uncommon in mammals, being found only in Suidae (6α-hydroxylation, 4 species), the Lesser Malay chevrotain (*Tragulus javanicus*, Tragulidae), and Muridae. The chevrotain and mammals within Suidae share the trait of having hyocholic acid (3α,6α,7α-trihydroxy-5β-cholan-24-oic acid), a 6α-hydroxylated bile acid, as their primary bile salt. Bile salts of Muridae use 6β-hydroxylated bile acids (e.g., 3α,6β,7α-trihydroxy-5β-cholan-24-oic acid and 3α,6β,7β-trihydroxy-5β-cholan-24-oic acid, also known as α-muricholic acid and β-muricholic acid, respectively).

### Relationship of diet to bile salt profile

To examine how diet might be related to bile salt profile, we divided mammals and reptiles into carnivores (diet > 90% meat), herbivores (diet > 90% plant matter), or omnivores (all remaining animals) (Additional Files 1 and 2). In our mammalian sample, there were 103 carnivores, 149 herbivores, and 74 omnivores. There were no clear differences between the different dietary groups in terms of percentage of animals that have CA as the major bile salt, CDCA as the major bile salt, or a C_24 _bile acid other than CA or CDCA as the major bile salt (Additional File 2). We also classified bile salt profiles as 'complex' or 'non-complex', with complex being defined as having a bile salt profile that includes two bile salt classes (C_27 _bile alcohols, C_27 _bile acids, or C_24 _bile acids) and/or the presence of three or more bile salts in bile, with each comprising 10% of greater of total biliary bile salts. Using this classification, 39.8% of carnivores, 70.5% of herbivores, and 50.0% of omnivores in our mammal sample had complex bile salt profiles.

In reptiles, the majority of animals analyzed were carnivores (n = 183), with fewer herbivores (n = 15) and omnivores (n = 17). In this sample, the majority of herbivores (66.7%) and omnivores (88.2%) use primarily C_27 _bile acids, a bile salt profile less common in carnivores (24.6%), but the herbivores and omnivores are mainly found in species from Testudines (Additional File 1). Using the classification of bile salt profile complexity defined in the above paragraph, there were few reptiles with complex bile salt profiles: 9.8% of carnivores, 6.7% of herbivores, and 5.9% of omnivores.

### Evolutionary variation of bile salts across reptiles, birds, and mammals

**Figure 6:**
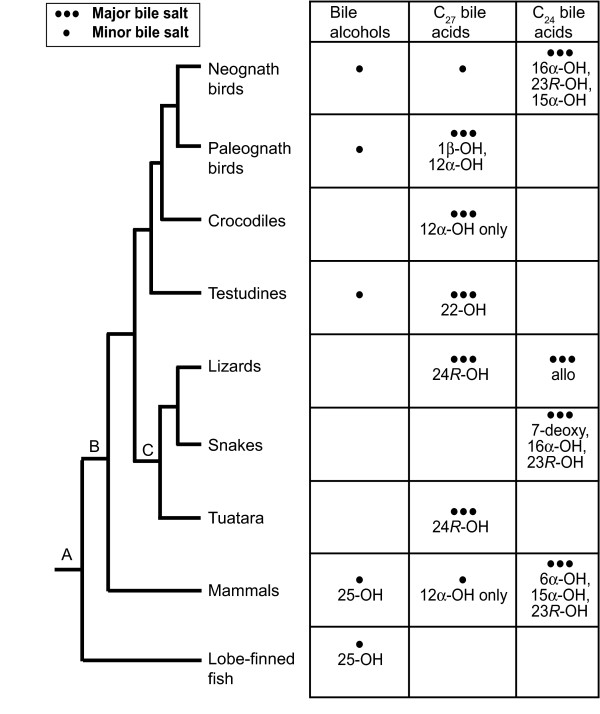
**Overall bile salt variation in reptiles, birds, and mammals**. The phylogeny depicted has Testudines as sister-group to Aves/Crocodylia. Lizards are paraphyletic but are indicated here as a single group for comparison purposes. Based on the patterns of bile salt variation across vertebrates, the ancestral bile salt profiles at nodes A, B, and C can be inferred by parsimony. The ancestral phenotype at node A would likely be C_27 _bile alcohols, the phenotype found in all living lobe-finned, jawless, and cartilaginous fish surveyed to date. The ancestral phenotype at node B would likely be C_27 _bile alcohols and trihydroxy-C_27 _bile acids, the major bile salts of Crocodylia and paleognath birds. The most recent common ancestor to tuatara, lizards, and snakes (node C) would additionally have the ability to produce 24*R*-hydroxylated C_27 _bile acids, the main bile salts of tuatara and lizards within Anguimorpha and Scinciformata. Based on the phylogeny depicted, there appear to be several 'innovations' unique to certain groups: (1) 24*R*-hydroxylation of C_27 _bile acids by lepidosaurs as mentioned above, (2) 22-hydroxylation of C_27 _bile acids by Testudines, and (3) production of 7-deoxy and C_23 _bile acids by snakes. The figure does not show all the known bile salt modifications, but only some of the more common ones within in each group.

Figure 6 shows a tree of reptiles, birds, and mammals, with lobe-finned fish as the outgroup. This tree fits the current molecular data and places Testudines as a sister-group to Crocodylia and Aves [37]. From these relationships, one can speculate about the bile salt profile of the most recent common ancestors at various nodes on the tree. For Testudines/Crocodylia/Aves, it can be inferred that the most recent common ancestor had a bile salt profile consisting mainly of C_27 _bile alcohols and a trihydroxy C_27 _bile acid (3α,7α,12α-trihydroxy-5β-cholestan-27-oic acid). This profile is still present in paleognath birds [4]. Crocodylia retain this trihydroxy C_27 _bile acid as their main primary bile salt but now produce only trace amounts of C_27 _bile alcohols. Testudines differ by having a (so far) unique modification to C_27 _bile acids, namely 22-hydroxylation (and in some cases additional 15α-hydroxylation). As noted above, two turtles in our survey have more than 10% C_27 _bile alcohols present as biliary bile salts and can be hypothesized to have retained the ancestral pathway for a significant fraction of their biliary bile salts.

For lizards/snakes/tuatara (Lepidosauria), 24*R*-hydroxylation of C_27 _bile acids is a trait shared by some lizards (e.g., varanids) and the tuatara. There have been a number of innovations in bile salt synthesis in snakes relative to other reptiles including 23*R*-hydroxylation, 7-dehydroxylation, 16α-hydroxylation, and production of C_23 _bile acids. It should be noted that a 24*R*-hydroxy C_27 _bile acid would lose the 24*R*-hydroxyl group upon side-chain shortening and oxidation to a C_24 _bile acid, so it is likely that 24*R*-hydroxylated C_27 _bile acids are produced as intermediates in the bile acid biosynthetic pathways of lizards and snakes that have primary bile salt profiles consisting of approximately 100% C_24 _bile acids.

**Figure 7:**
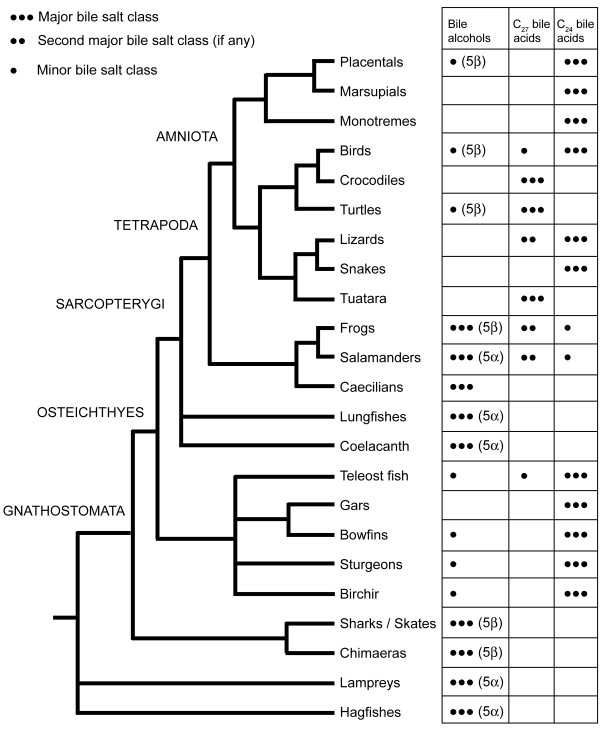
**Overall bile salt variation across vertebrates**. Variation of bile salts is overlaid on a vertebrate phylogeny, with turtles placed as sister group to birds/crocodiles, frogs and salamanders as sister groups, and placental mammals and marsupials as sister groups [37]. Lizards are paraphyletic but are indicated here as a single group for comparison purposes. Unresolved relationships are depicted as polyotomies. The figure shows two "shifts to the right" from 5α-C_27 _bile alcohols to C_24 _bile acids as the bile salt synthetic pathway presumably grew in length: (1) from Agnatha to ray-finned fish and (2) from lobe-finned fish to tetrapods. Note that C_27 _bile acids are common in reptiles and amphibians but uncommon in fish. Within amphibians, there is only preliminary data on caecilians based on analysis of two specimens, both of which showed only bile alcohols but with the orientation of the 5-hydroxyl group as yet undetermined pending additional analyses (LR Hagey, unpublished data).

**Figure 8:**
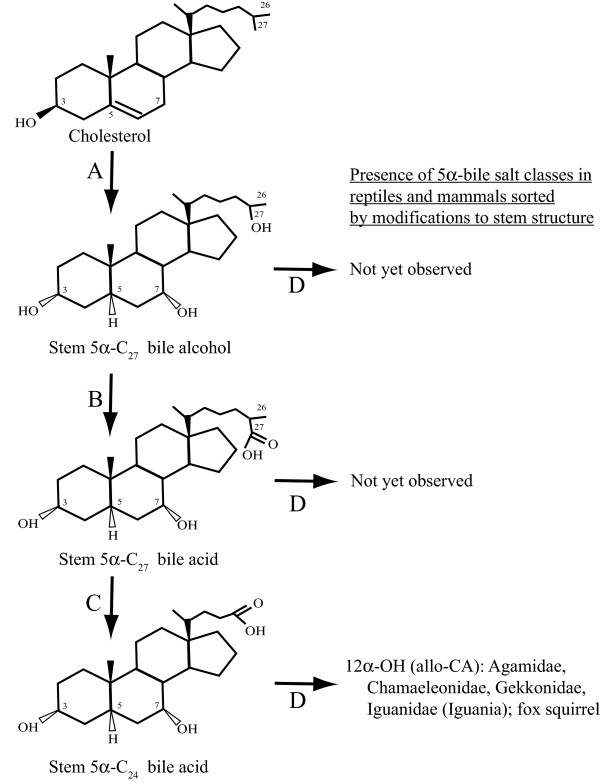
**Illustration of 5α bile salts**. The 5α series of C_27 _bile alcohol, C_27 _bile acid, and C_24 _bile acid structures are illustrated with examples of animals (if any) whose major bile salts are in these classes. The structures are the stem structures for each of the three major classes of bile salts. Multiple enzymes are involved in the transition indicated by arrow A. The enzymes responsible for 5α-reduction of bile salt precursors are currently unknown. If 5α-bile salt pathways are homologous to 5β pathways, the reaction indicated by arrow B would be carried out by CYP27A1. Side-chain cleavage (arrow C) is likely a peroxisomal reaction in most or all animals. Arrow D covers all the possible additional modifications to stem bile salt structures (e.g., additional hydroxylation of the nucleus or side-chain, introduction of oxo groups, unsaturation of the side-chain, etc.).

**Figure 9:**
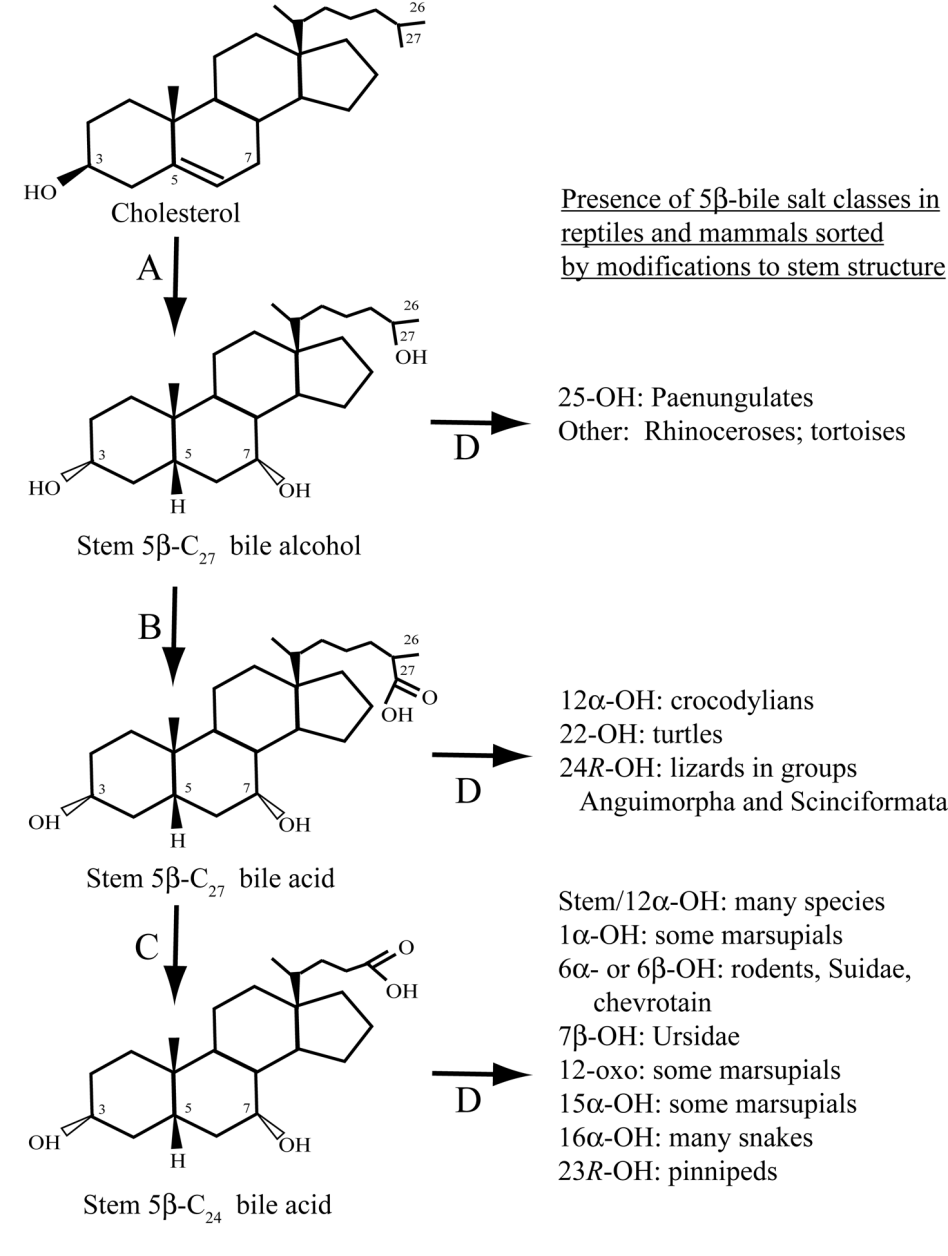
**Illustration of 5β bile salts**. The 5β series of C_27 _bile alcohol, C_27 _bile acid, and C_24 _bile acid structures are illustrated with examples of animals (if any) whose major bile salts are in these classes. The structures are the stem structures for each of the three major classes of bile salts. Multiple enzymes are involved in the transition indicated by arrow A. Arrow B is carried out by CYP27A1 in humans and rodents. Side-chain cleavage (arrow C) is likely a peroxisomal reaction in most or all animals. Arrow D covers all the possible additional modifications to stem bile salt structures (e.g., additional hydroxylation of the nucleus or side-chain, introduction of oxo groups, unsaturation of the side-chain, etc.).

Figure 7 depicts the variation of bile salts among different groups of vertebrates using a generic vertebrate phylogeny (with unresolved relationships depicted as polyotomies) [37]. Ray-finned fish share the common trait of using C_24 _bile acids with snakes, many lizards, most birds, and most mammals [9]. The inferred ancestral bile salts (5α-bile alcohols) are essentially not found in extant species outside Agnatha (hagfish, lampreys), lobe-finned fish, some teleost fish (e.g., Cypriniformes), and some amphibians (Figure 8). However, other than fish and amphibians, 5β-bile alcohols are found in some reptiles, birds, and mammals (Figure 9), either as minor but significant (> 10%) fraction of total biliary bile salts (e.g., tortoises, paleognath birds) or as the major primary bile salts (e.g., Paenungulates, rhinoceroses). C_27 _bile acids are common in reptiles (Crocodylia, varanid lizards, turtles), amphibians, and paleognath birds but uncommon in fish and mammals.

### Analysis of human and extinct ground sloth coprolites

To determine whether bile salts could be recovered from paleontological specimens older than 5 000 years, we analyzed coprolite samples from ancient humans and the extinct Shasta ground sloth *Nothrotherium shastense*. The human coprolite samples had been collected from the Danger Cave Archaeological Site, Utah, USA, and dated as approximately 8,000 years old by radiocarbon analysis (see Methods). Bile acids were readily recovered from the human coprolite samples including the primary bile acid CA as well as the secondary bile acids lithocholic acid (3α-hydroxy-5β-cholan-24-oic acid; LCA), 12-oxo-LCA (3α-hydroxy-12-oxo-5β-cholan-24-oic acid), DCA, and 7-oxo-DCA (3α,12α-dihydroxy-7-oxo-5β-cholan-24-oic acid) (Additional File 3, Figure S3BB), a profile similar to bile acids in human cecal contents obtained from autopsy specimens [38].

The Shasta ground sloth coprolite was collected from Rampart Cave in Arizona (see Methods). The bile acid concentrations in the Shasta ground sloth coprolite samples were lower than the human coprolite specimen. To enable sensitivity at the low concentrations of bile acids present, the samples were analyzed by ESI/MS/MS. This analysis revealed peaks with *m/z *ratios consistent with the presence of glycine-conjugated mono-, di-, tri-, and tetra-hydroxylated C_24 _bile acids, along with additional peaks with *m/z *ratios missing two or four protons, consistent with either conversion of hydroxyl groups into oxo groups, or the presence of singly- and doubly-unsaturated C_24 _bile acids, or a mixture of both (Additional File 3, Figure S3CC). Taurine-conjugated bile acids were not detected. A complex mixture of plant sterols and stanols were also present, consistent with an herbivorous diet. (Additional File 3, Figure S3DD). Our samples of living mammals included two sloth species: *Bradypus tridactylus *(white-headed sloth) and *Bradypus variegatus *(brown-throated three-toed sloth). Both sloth species had ~100% glycine-conjugated bile acids in their bile, including the unusual bile acid 7-oxo-DCA (Additional file 2). We also analyzed the fecal bile salts from Hoffmann's two-toed sloth (*Choloepus hoffmanni*; Supplemental Figures S3Y, Z, AA). The fecal bile salts from this sloth showed a mixture of glycine-conjugated and unconjugated bile acids, similar to the ground sloth coprolites. In our mammalian biliary bile survey, only 11 of 326 species surveyed had ~100% glycine conjugation of bile acids, supporting the conclusion that the bile acids identified are indeed from a sloth.

### Comparison of bile salt enzymes across species

As discussed in the Introduction, there are at least 16 enzymes involved in bile salt synthetic pathways in humans and rodents, with two main pathways (neutral and acidic) identified so far [6,7]. Figure 2 shows a simplified version of the neutral bile salt synthetic pathway. The minimal synthetic pathway for a functional bile salt would involve five enzymes in the production of a stem C_27 _bile alcohol with three hydroxyl groups, a bile salt actually found in a small number of animals surveyed so far (e.g., ratite birds) (Figures 8 and 9).

The first enzyme is cytochrome P450 (CYP) 7A1 which converts cholesterol to 7α-hydroxycholesterol. The next enzyme in the neutral enzyme, 3β-hydroxysteroid-Δ^5^-C_27 _steroid oxidoreductase (HSD3B7), catalyzes the simultaneous isomerization of the C-5 - C-6 double bond to C-4 - C-5 and oxidation of the 3β hydroxyl group to a 3-oxo group. The third enzyme, aldo-keto reductase 1D1 (AKR1D1), mediates stereospecific reduction of the Δ^4 ^double bond to form a 5β (A/B *cis*) steroid. The fourth enzyme, AKR1C4, reduces the 3-oxo group to a 3α-hydroxy group [39]. The fifth and final enzyme is the mitochondrial C-27 hydroxylase (CYP27A1) that results in the formation of a C_27 _bile alcohol. The multi-functional CYP27A1 also has the ability to catalyze the conversion of a C_27 _bile alcohol to a C_27 _bile acid by oxidation of the hydroxyl group on the terminal carbon atom of the side-chain to a carboxylic acid group. These five enzymes (CYP7A1, HSD3B7, AKR1D1, AKR1C4, and CYP27A1) dictate three key properties of bile salts: (1) orientation of the C-3 hydroxyl group (3β in hagfish bile alcohols and 3α in all other known primary bile salts; rarely, there can be a 3-oxo instead of a hydroxyl group), (2) orientation of the hydrogen atom at C-5 (5α or 5β), which influences the overall configuration (bent or planar) of the steroid rings, and (3) synthesis of C_27 _bile alcohol versus further oxidation to C_27 _bile acid. The remaining 11 enzymes identified so far in the bile salt synthetic pathway catalyze a number of reactions including additional hydroxylation (e.g., 12α-hydroxylation by CYP8B1), side-chain shortening and oxidation (5 peroxisomal enzymes), or alternative reactions such as found in the acidic pathway of bile acid synthesis [6,7].

We utilized BLAST searches to try to identify putative orthologs of the human enzymes involved in bile salt biosynthesis [6,7]. While assignment of true orthology depends on both sequence comparison and biochemical verification, tentative orthology can be assigned by reciprocal BLAST searching. Caution needs to be especially applied to comparisons of enzymes that are part of large superfamilies (e.g., aldo-keto reductases, CYPs). Indeed, we were not able to clearly identify putative orthologs in non-mammalian species to the gene for human AKR1C4.

Currently, of all the ongoing vertebrate genome sequencing projects, there are many mammalian species but only one reptile, the green anole lizard (*Anolis caroliensis*). The green anole lizard is of interest with respect to bile salt variation in that this species has nearly 100% 5α (allo) bile acids. Indeed, of the 8 species within Iguanidae analyzed, most had greater than 85% 5α bile acids and all had at least 50% 5α bile acids (Additional File 1). The unusual bile salt profile of the anole lizard thus raises the question of how this animal synthesizes a bile salt pool of mainly 5α bile acids. Of the model mammalian species in genome sequencing projects, two are Paenungulates (African elephant, *Loxodonta africana*; rock hyrax, *Procavia capensis*) that have bile salt profiles consisting of nearly 100% bile alcohols, a phenotype also found in jawless fish, cartilaginous fish, lobe-finned fish, and some amphibians, but not yet observed in any reptile or bird species. The elephant and rock hyrax can serve as examples to address how an animal maintains a bile salt pool of C_27 _bile alcohols without producing detectable amounts of bile acids.

Focusing on AKR1D1, the enzyme that catalyzes 5β-reduction of the steroid nucleus, we found that putative orthologs to the human enzyme could be found in mammals, birds, green anole lizard, *Xenopus *frogs, and multiple teleost fish (Additional File 4). Interestingly, an AKR1D1 ortholog could not be located for zebrafish [9], the only model teleost fish for genome sequencing studies that exclusively produces 5α bile salts. In contrast, an AKR1D1 ortholog was readily identified for the anole lizard, with an amino acid sequence identity of 78.2% to human AKR1D1, similar to the sequence identity of 81.6% between human and chicken AKR1D1. Although an AKR1D1 ortholog could not be detected in the zebrafish genome, we did find putative zebrafish orthologs to the mammalian genes for 5α-reductase 1 (SRD5A1) and 2 (SRD5A2): Genbank: NM_001076653 and Genbank: NM_001017703, respectively.

Focusing next on CYP27A1, the enzyme that in some species catalyzes 27-hydroxyation of bile salt intermediates to form C_27 _bile alcohols and in other species, continues the oxidation of the side-chain to form C_27 _bile acids, we found putative orthologs to the human enzyme throughout vertebrates, including African elephant, rock hyrax, anole lizard, and zebrafish. The elephant, hyrax, and zebrafish all have bile salt profiles consisting of C_27 _bile alcohols and thus would be predicted to have CYP27A1 enzymes unable to further catalyze the oxidation of the side-chain from a C_27 _bile alcohol to C_27 _bile acid. As mentioned above, the anole lizard and zebrafish synthesize 5α bile salts; consequently, CYP27A1 enzymes in these species would need to accommodate bile salt precursors with 5α orientation.

We also identified putative orthologs in many species, including African elephant and zebrafish, to the four peroxisomal enzymes (2-methylacyl-CoA-racemase, branched-chain acyl-CoA oxidase, D-bifunctional protein, and peroxisomal thiolase 2) involved in shortening of the side-chain to produce C_24 _bile acids. In fact, candidates for orthologs to all four peroxisomal enzymes were identified in the genome of the zebrafish, an animal that does not shorten the side-chain of its bile salts (Additional File 3). This is not necessarily surprising in that these enzymes also fulfil other cellular functions such as β-oxidation of fatty acids [6,7]. However, it does raise the question of how animals that produce only C_27 _bile salts (e.g., Paenungulates, zebrafish) retain the C_8 _side-chain of cholesterol.

Lastly, for four of the bile salt synthetic enzymes (CYP46A1, AMACR, ACOX2, HSD17B4), we identified putative orthologs in the genomes of model invertebrates such sea squirt (*Ciona intestinalis*), Pacific sea squirt (*Ciona savignyi*), and purple sea urchin (*Strongylocentrotus purpuratus*). Invertebrates are not known to produce bile salts, so these enzymes likely serve other functions. These enzymes are thus candidates for the study of how protein functions changed during the evolution of invertebrates to vertebrates.

## Discussion

We have greatly expanded previous surveys of bile salt structural diversity in reptiles and mammals. The variation of bile salts is consistent with current molecular-based phylogenies of reptiles and mammals, including the recent reclassification of squamate groups based on DNA sequencing data and studies showing that pinnipeds form a monophyletic group with Carnivora [35]. Perhaps the most unusual finding in our study was that the Paenungulates (elephants, manatees, and the rock hyrax) have a very different bile salt profile from the Rufous sengi and South American aardvark, two other mammals classified with Paenungulates in the cohort Afrotheria by molecular phylogenetic analysis. It will be of interest to determine the molecular basis for these differences. As we discuss below, this finding could be accounted for by differences in a single enzyme, CYP27A1.

Our analysis did not reveal any obvious relationship between diet and bile salt profiles, although a major caveat to this analysis is the non-random nature of the animals found in our survey of reptiles and mammals. In reptiles, the analysis is further limited by the preponderance of carnivores in the surveyed sample (e.g. snakes, varanid lizards, crocodilians), with far fewer herbivores or omnivores. We also did not find any evidence that bile salt complexity is associated with a more complex omnivorous diet in either mammals or reptiles. The lack of association of bile salt profile and diet is also suggested by groups of animals that have very similar bile salt profiles but different diets. Examples include primates, Ursidae (pandas as herbivores, other bears as carnivores or omnivores), other animals in Carnivora excluding pinnipeds, and Testudines. Overall, common bile acids such as CA and CDCA, or other C_24 _bile acids such as ursodeoxycholic acid, are found in species with varied diets. With uncommon bile acids, there is insufficient data to make generalizations. For example, pythocholic acid (Boidae and Pythonidae) or varanic acid (Varanidae) could theoretically be advantageous to a carnivorous diet, pending more extensive surveys of reptiles and mammals.

Our analysis of ancient human and extinct giant ground sloth coprolites demonstrates the stability of bile acids in biological specimens preserved in arid climates. Previous studies have analyzed bile acids and sterols from human coprolites or internal organs preserved within mummies, with ages ranging from approximately 500 to 3,200 years old [40-42]. Our results suggest that bile salts may be useful markers in select paleontological studies.

Our use of multiple analytical techniques (e.g., HPLC, ESI/MS/MS, HPLC, NMR) allowed us to precisely resolve complicated bile salt profiles. We speculate on what these findings mean for the understanding of the evolution of bile salt synthesis, a complex and key biochemical pathway that permits cholesterol excretion to be regulated and at the same time generates amphipathic molecules with multiple physiological functions. In analyzing the patterns of bile salt variation across vertebrates, there appears to be at least two major pathways in the evolutionary transition from C_27 _bile alcohols (ancestral) to C_24 _bile acids (derived): (a) a 'direct' pathway and (b) an 'indirect' pathway that uses C_27 _bile acids as an 'intermediate' step. Evidence to support pathway (a) comes mainly from teleost fish families (e.g., Perciformes) where either type III (C_27 _bile alcohols and C_24 _bile acids but no C_27 _bile acids) or type VI (C_24 _bile acids only) are found, but not fish with any appreciable amount of C_27 _bile acids [9]. This suggests that the ancestors of these fish followed an evolutionary transition from C_27 _bile alcohols directly to C_24 _bile acids. This differs from reptiles (e.g., Crocodylia, Testudines, varanid lizards), some mammals, and amphibians, where C_27 _bile acids are common [2,3,5].

One of the major goals of our study is to provide testable hypotheses for determining what bile salt enzyme differences underlie cross-species differences in bile salt profiles. Cross-species comparisons have already yielded some insights into different in bile salt profiles. An example is the discovery in pigs of CYP4A21, an enzyme that catalyzes 6α-hydroxylation of bile acids [43], a bile acid modification not found in humans. One unanswered question is how a bile salt synthetic pathway 'stops' at C_27 _bile alcohols, as must occur in animals such as Paenungulates, rhinoceroses, and zebrafish whose bile salt profiles do not show C_27 _or C_24 _bile acids. One possible explanation would involve the multi-functional capabilities of CYP27A1. Animals synthesizing only C_27 _bile alcohols would have CYP27A1 enzymes that can 27-hydroxylate but not oxidize the side-chain of the resulting C_27 _bile alcohols. It would be of interest to test the substrate specificities of CYP27A1 from species that synthesize mainly bile alcohols. No high-resolution crystal structure of CYP27A1 is currently available, but a homology model of human CYP27A1 has been developed [44] which could be used for comparative purposes to rationalize cross-species differences in substrate and catalytic specificity.

There is the additional question of when did the fatty acid and bile salt β-oxidation enzymes develop in peroxisomes. Three of these enzymes have strong candidates for invertebrate orthologs, suggesting a long evolutionary history, but the parallel ability for bile salt β-oxidation must have been a strictly vertebrate innovation given the occurrence of bile salts only in vertebrates. The zebrafish is an example of an animal that produces only C_27 _bile salts, yet possesses putative orthologs to genes for all four peroxisomal enzymes involved in shortening of the bile acid side-chain in mammals [9]. Evolutionary changes of peroxisomal transporters such as ATP-binding cassette transporter D3 (ABCD3) [45,46] may have been important in this regard by facilitating substrate entry into the peroxisomes (beyond passive diffusion), possibly explaining how an animal can maintain a bile salt profile of mainly C_27 _bile acids while having all the enzymes capable of C_27 _bile salt side-chain cleavage to form a C_24 _bile acid. There have been limited studies of peroxisomes in non-mammalian species, and we are not aware of any published studies of this organelle in reptiles. Detailed studies of peroxisomes in reptile groups that produce only C_27 _bile acids (e.g., turtles, varanid lizards, crocodilians) could be especially helpful in understanding cross-species differences in bile salt biosynthesis, particularly modification of the side-chain.

Genomic comparisons may also provide some potential insight into the synthesis of 5α-bile salts. The sporadic appearance of 5α-bile salts in otherwise evolutionarily recent animals suggests that it is the result of mutation in a biosynthetic enzyme (a likely candidate is AKR1D1). Trace levels of 5α-bile acids are ubiquitous in bile, but the origins of these small concentrations are likely produced by microbial flora and not from biosynthesis [47]. The presence of 5α-bile acids in the bile of germ-free rabbits indicates that at least some mammals are capable of synthesizing 5α-bile acids [48]. The hepatic enzymes(s) (if present) that perform 5α-reduction of bile salt intermediates like 4-cholesten-7α,12α-diol-3-one are as yet uncharacterized [7].

Model animals such as the anole lizard and zebrafish may be helpful in determining the enzymatic pathways for generating 5α bile salts. For example, we did not find a putative ortholog to the gene for AKR1D1 in the zebrafish genome. We did, however, find likely orthologs to the mammalian genes for 5α-reductase 1 (SRD5A1) and 2 (SRD5A2) in the zebrafish genome. It is possible that either or both of these enzymes, or another enzyme altogether, catalyzes 5α-reduction of the steroid nucleus in zebrafish bile salt biosynthesis. In contrast, a putative ortholog for AKR1D1 was identified in the genome of the anole lizard. Studies of anole lizard AKR1D1 are required to see if this enzyme catalyzes 5α-reduction of bile acid intermediates (unlike human and rodent AKR1D1) or 5β-reduction for the trace amounts 5β-bile acids present in anole bile. There are also ongoing sequencing projects for sea lamprey (*Petromyzon marinus*) and coelacanth (*Latimeria calumnae*), two animals that exclusively produce 5α bile alcohols [9,12,49]. Comparison of the genomes of anole lizard, zebrafish, lamprey, and coelacanth may also provide insight into how they achieve a bile salt pool of 5α-bile salts. Additional File 5 includes a summary of model animals whose study may provide insight into the mechanisms underlying cross-species differences in bile salt profiles.

Our study does not shed light on how animals regulate their bile salt profiles. In Additional File 3 we include some spectra focusing on the minor bile salts of various species revealing there can be a complex mixture of bile alcohols at low concentration even in species whose biliary bile salts are comprised of greater than 95% bile acids. Whether these minor bile salts perform physiological functions or are simply vestigial is currently unknown. It is logical to assume to that both bile acid biosynthesis and intestinal conservation are mainly controlled by transcriptional regulators, although this is not well-understood even in mammals.

There is still little understanding of what drives variation in bile salt chemical diversity. As our analysis of reptiles and mammals shows, there is essentially no apparent link between diet and bile salt structure. Typical 5β-C_24 _bile acids are found in reptiles and mammals with a wide variety of diets. It should be noted that all described hydroxylation sites occur on the hydrophilic face of the bile salt molecule, keeping the amphipathicity intact [1]. It is also unclear what benefit may be conferred to those reptiles (e.g., in the group Iguania) that use 5α bile salts. Studies in mammals have shown that 5α bile acids can be toxic. One striking example of this occurs in the rabbit. Rabbits fed 5α-cholestanol (the saturated homolog of cholesterol) form 5α-cholic acid, which is 7-dehydroxylated by gut bacteria to form 5α-deoxycholic acid and subsequently conjugated with glycine in the liver. However, the glycine conjugate of 5α-deoxycholic acid acid precipitates from solution in the gallbladder, forming gallstones [50,51]. Animals with a bile salt pool of mostly 5α-bile acids evidently have mechanisms to avoid toxicity.

The bile salt biosynthetic pathway has apparently evolved in vertebrates in a considerably different fashion from the adrenocortical and sex steroid hormones, another set of compounds derived from cholesterol. Estrogens are the terminal products of the steroid hormone pathway whereas other steroids such as glucocorticoids or mineralocorticoids are intermediates. The actions of the steroid hormones are mediated by nuclear hormone receptors. Thornton and colleagues have used a variety of techniques, including phylogenetic reconstruction and expression of ancestral proteins, to provide evidence that estrogen receptors have a longer evolutionary history than receptors for intermediate products (e.g., glucocorticoids) in the steroid hormone pathways [52-54] (although their ancestral reconstruction in invertebrates have been challenged by other research [55]). In the 'molecular exploitation' model of a biochemical pathway, the terminal products of a synthetic pathway mediate the more ancestral activities. During evolution, functions (and the receptors that mediate these functions) develop for the intermediate products of the pathway, and structural diversity involving intermediates in the pathway can increase over time. In the case of the steroid hormones, the major evolutionary changes probably occurred in early vertebrate evolution or even earlier.

The diversity of bile salts, starting in fish and amphibians [9] and combined with the data for reptiles and mammals in the present study, suggests a different model of evolution of bile salts from steroid hormones. In the case of bile salts, the terminal products are increasing in diversity throughout all vertebrate classes. A parallel to the bile salt synthetic pathway may be found in the enzymatic pathways involved in the formation of triterpenoids, isoprene-derived 30-carbon molecules that serve as a building block for many other compounds. Studies of bacteria, plants, and animals demonstrate at least two independent pathways for the formation of isoprene units, each involving multiple organelles [56]. Similar to bile salts, the synthetic intermediates in the triterpinoid pathways are low in diversity but the end-products have impressive biochemical diversity and mediate an array of different physiological functions. Like triterpinoids, the end-products of bile salt synthesis have substantial structural diversity by variations in hydroxylation patterns, unsaturation, and side-chain length. Snakes and marsupials are two groups that demonstrate ongoing 'innovations' in bile acids not seen in fish and amphibians (e.g., 1α- and 1β-hydroxyation in the spotted cuscus and feathered glider, respectively; and Δ22 and C_23 _bile acids in true viper snakes).

Bile acid synthesis is now known to be catalyzed in humans and rodents by at least two independent pathways (acidic and neutral), with tight regulation keeping intermediate products at very low concentrations in the hepatocytes and biliary bile [6,7]. In some animals such as the crotaline snakes, the result is a bile salt pool consistently of almost 100% of a single bile salt molecule such as CA. The bile salt synthetic pathway has evolved from a relatively simple pathway that produces C_27 _bile alcohols (by simple saturation and polyhydroxylation of cholesterol as in hagfish) [9] to more complex pathways that utilize multiple organelles (cytoplasm, endoplasmic reticulum, mitochondria, and peroxisomes) to produce C_24 _bile acids. In the process, many ray-finned fish and most land animals have bile containing almost exclusively C_24 _bile acids and very low amounts of the synthetic precursors including C_27 _bile alcohols, a process achieved by the evolution of regulatory control and enzyme modifications.

Although there is extensive data on the physicochemical and physiological properties of common bile acids such as CA and CDCA (and their conjugated derivatives), there has been much less study of other classes of bile salts. In part, this relates to the difficulty of synthesizing or isolating sufficient material to perform detailed studies. The complicated variation of bile salts may also relate to other functions of bile salts that are not well understood, including communication, influencing of the microbial environment in the gut, and regulation of hepatobiliary development and regeneration [1]. It has been proposed that bile acid structural variation may be driven by attempts to prevent gut microbial 'damage' of bile acids that can lead to toxic secondary bile acids such as DCA and LCA [1,8,57]. Novel modifications to the stem bile salt structures throughout vertebrate evolution make it difficult for bacteria to alter the bile salt structure into molecules that are toxic or have poor aqueous solubility or both.

Finally, the extensive variation of bile salt structures across vertebrate species predicts that protein receptors that bind bile salts will also show variation in structure and ligand-binding specificity. Indeed, research has already shown distinct patterns of ligand-receptor co-evolution for three nuclear hormone receptors (farnesoid X receptor, FXR; pregnane X receptor, PXR; and vitamin D receptor, VDR) that regulate various aspects of bile salt synthesis, transport, or metabolism [10,14,58-60]. Model species that have bile salt profiles different from humans, such as anole lizard, rock hyrax, and African elephant, may be particularly interesting to study, as their bile salt-regulating nuclear hormone receptors would have to bind different ligands (e.g., bile alcohols or 5α-bile salts) than their human or rodent orthologs which need to recognize C_24 _5β-bile acids. Ultimately, we aim to build up a complete multi-dimensional picture of ligand, protein, commensal microbial, and species evolution involving bile salts.

## Conclusions

Bile salts are a biochemical product of varying structures that tend to be conserved within families or orders of vertebrates. Variations in bile salts do not correlate with diet. Analysis of the evolution of bile salt synthetic pathways provides a rich model system for the evolution of a complex biochemical pathway in vertebrates. Our results also demonstrate the stability of bile salts in coprolites preserved in arid climates suggesting that bile salt analysis may have utility in selected paleontological research.

## Methods

### Bile samples

Bile samples were obtained from a number of sources (see Acknowledgements) over the course of two decades of analysis. A major portion of the samples analyzed were obtained during necropsy of animals that died in captivity at the San Diego Zoo. An additional set of samples analyzed were from an extensive collection of bile samples of the late G.A.D. Haslewood, donated to the University of California - San Diego. Many samples were taken from newly dead animals, but a few were obtained from preserved museum specimens. No animals were specifically sacrificed for the purposes of this study. All animal studies were performed in conformity with the Public Health Service Policy on Humane Care and Use of Laboratory Animals, incorporated in the Institute for Laboratory Animal Research Guide for Care and use of Laboratory Animals. All vertebrate animal studies were approved by the Committee on Animal Studies of the University of California, San Diego. Fecal samples from the Hoffmann's two-toed sloth (*Choloepus hoffmanni*) were generously collected by staff at the Pittsburgh Zoo.

### Coprolite samples

Coprolites of the Shasta ground sloth were collected in 1971 by Dr. P.S. Martin from Rampart Cave, Arizona, USA, located in the Grand Canyon of the Colorado River. Two publications detail the analyses of Shasta ground sloth coprolites from Rampart Cave [61,62]. Radiocarbon dating revealed ages ranging from approximately 10,000 years old for dung on the cave surface to 35,000 years old for dung located 54 inches below the surface [62]. The Shasta ground sloth is thought to have gone extinct approximately 11,000 years ago [16]. The specimen analyzed consisted of loose, coarse, light-colored fragments. The coprolite samples were acquired and archived by the San Diego Zoo. The human coprolite samples were from Danger Cave Archaeological Site (Hind Cave #3), Utah, USA, and collected in 1986-1987 by Dr. R.Z. Jones.

### Analysis of bile salts

A number of different analytical methods were used to analyze the bile salts found in animal bile. Thin-layer chromatography (TLC) was helpful in identifying which classes of bile salts (bile alcohols, C_27 _bile acids, C_24 _bile acids) were present. Depending on the results of TLC analysis, the samples were subjected to further analyses optimized for the bile salt classes present. For C_24 _bile acids and some C_27 _bile acids, high-performance liquid chromatography (HPLC) analysis with suitable reference standards was sufficient to resolve the bile salt profiles. When possible, samples were further subjected to electrospray ionization mass spectrometry/mass spectrometry (ESI/MS/MS) analysis. Gas chromatography/mass spectrometry (GC/MS) was helpful when the orientations of hydroxyl or hydrogen groups were not certain from other analyses. However, GC/MS requires relatively large amounts of sample and could not be applied if limited bile was available. For previously unknown bile salts, nuclear magnetic resonance (NMR) was needed to provide definitive structural identification.

#### Thin-layer chromatography (TLC)

Whole bile was separated on silica gel G (E. Merck, Darmstadt, Germany) using two solvent systems: (1) isoamyl acetate: propionic acid: 1-propanol: water 4: 3: 2: 1 (v/v) [63]; and (2) a double development system for the resolution of conjugated bile acids, in which the plates were developed first in chloroform: methanol: water: acetone: propionic acid, 10: 2: 1: 4: 1 (v/v), and after drying overnight, followed by 1-butanol: propionic acid: water, 10: 1: 1 (v/v) [64]. Bile acids were visualized by spray reagents for hydroxyl groups (phosphomolybdic acid, 10% w/v in ethanol), oxo groups (2,4-dinitrophenyl-hydrazine), sugars (naphtholresorcinol), or vicinal hydroxyl group (lead tetra-acetate).

#### High-performance liquid chromatography (HPLC)

Conjugated bile acids were analyzed by HPLC using a modification of a previously reported technique [65]. An octadecylsilane column (RP C-18, Beckman Instruments, Fullerton, CA, USA) was used with isocratic elution at 0.75 mL/min. The eluting solution was composed of a mixture of methanol and 0.01 M KH_2_PO_4 _(67.4% v/v), adjusted to an apparent pH of 5.35 with H_3_PO_4_. Conjugated bile acids were quantified by measuring the absorbance of their amide bond at 205 nm. Unconjugated bile acids and bile alcohol sulfates are not detected by this method. Bile acids were tentatively identified by matching their relative retention times with those of known standards.

### Mass spectrometry

#### Electrospray ionization mass spectrometry/mass spectrometry (ESI/MS/MS)

Biliary contents were dissolved and diluted in methanol (Burdick & Jackson, Muskegon, MI, USA) and analyzed using ESI/MS/MS on a Hewlett-Packard HP 1100 MSD operated in the negative mode. The high-performance liquid chromatography (HPLC) column was removed, and the injector output coupled directly to the ESI inlet. Samples (2 μL) were injected in methanol: water, 90:10 (v/v) mobile phase running at a flow rate of 0.35 mL/min. The fragmenter was set to 200 V and the capillary voltage set to 5000 V.

#### Liquid secondary ion mass spectrometry (LSI-MS)

LSI-MS was done at the Bio-organic Biomedical Mass Spectrometry Resource (University of California - San Francisco, San Franciso, CA, USA) using a Kratos VG 70-SE mass spectrometer (Manchester, UK). This instrument used a cesium ion source and operated at approximately 100 μA/cm^2 ^beam flux in the negative ion mode. The instrument was operated at an accelerating voltage of 8 kV and a mass resolution of 1000 (10% valley definition). The liquid matrix was glycerol on a copper-tip probe.

#### Gas chromatography/mass spectrometry (GC/MS)

Glycine and taurine conjugates of bile acids were deconjugated chemically using 1.0 *N *NaOH at 130°C for 4 hours. Bile alcohol sulfates were deconjugated enzymatically. Unconjugated bile acids were isolated by acidification and extraction into ethyl acetate. They were then analyzed by capillary GC/MS as methyl ester acetates (prepared using acetic anhydride in acetic acid with perchloric acid catalyst) or as methyl ester trimethylsilyl derivatives (prepared using Tri-Sil, Pierce Chemicals, Rockford, IL). GC was performed using a Hewlett-Packard 5890 Gas Chromatograph-5970 MSD, controlled by HP/UX Chem Station software. The column was a 30 m 0.25 mm ID intermediate polarity SPB-35 of 35% phenyl methyl silicone (Supelco Co., Bellefonte, PA) operated at 277°C (isothermal). A splitless injection was used with an injection temperature of 290°C. Helium was used as the carrier gas with a 7 psi column head pressure. Relative retention times and fragmentation spectra of peaks obtained by GC/MS were compared with those of known standards for identification.

#### Nuclear magnetic resonance (NMR)

Carbon-13 NMR (^13^C-NMR) spectra of deconjugated methyl ester per acetyl derivatives of bile acids were also recorded. Multiplicities were determined with the APT sequence. Chemical shifts were recorded in ppm relative to tetramethylsilan. The central peak of the signal of Cl_3_CD was used as a reference (δ77.0 ppm).

## Authors' contributions

LRH performed the analysis of bile salts in vertebrate species, with many species analyzed while working in the laboratory of AFH. NV prepared the phylogenies for reptiles, collating data from multiple studies. All authors helped with the analysis of the data and comparison with other studies on the evolution of vertebrates. MDK performed the comparative analysis of bile salt synthetic enzyme gene sequences and drafted the manuscript. All authors contributed to, read, and approved the final manuscript.

## Supplementary Material

Additional file 1 **Bile salts of reptiles**. Table contains data on the bile salts of all reptiles analyzed, with bile salt profiles color-coded by bile salt class. Click here for file

Additional file 2 **Bile salts of mammals**. Table contains data on the bile salts of all mammals analyzed, with bile salt profiles color-coded by bile salt class. Click here for file

Additional file 3 **Annotated bile salt profiles of reptiles and mammals**. Examples of bile salt profiles (annotated ESI/MS/MS and GC/MS spectra) for reptiles and mammals are included. Click here for file

Additional file 4 **Bile salt synthetic enzymes**. Excel spreadsheet contains the accession numbers for putative orthologs to the enzymes involved in bile acid biosynthesis in humans. For genes where reliable full-length sequences are available, the percent identity of the amino acid. Click here for file

Additional file 5 **Model animals that may provide key insight into bile salt biosynthesis**. Table compares and contrasts differences of bile salt enzymes between 5 model species that have interesting bile salt profiles. Click here for file
