# Supplementary material for: Evolutionary diversity of bile salts in reptiles and mammals, including analysis of ancient human and extinct giant ground sloth coprolites
Source: BMC Evol Biol. 2010 May 6;10:133. doi: 10.1186/1471-2148-10-133 (PMC2886068; doi:10.1186/1471-2148-10-133)
Supplement: Additional file 1 — Bile salts of reptiles. Table contains data on the bile salts of all reptiles analyzed, with bile salt profiles color-coded by bile salt class. [file 1471-2148-10-133-S1.PDF]

Additional file 1: Bile salts of reptiles

Summary of detailed categorization by bile salt class

Class	Description	Number of reptile species
I	**Only C_{27} bile alcohols**	0
II	**Mixture of C_{27} bile alcohols and C_{27} bile acids**	2 (Siebenrock's snake-neck turtle and California desert tortoise)
III	**Mixture of C_{27} bile alcohols and C_{24} bile acids**	0
IV	**Only C_{27} bile acids**	67
V	**Mixture of C_{27} bile acids and C_{24} bile acids**	5
VI	**All C_{24} bile acids**	146
	**Total**	**219**
	**Mostly 5α (A/B *trans*, 'allo')**	35

Summary of diet class sorted by primary bile salt

Diet class	Total #	CA	CDCA	Other C_{24} bile acid	C_{27} bile acid	C_{27} bile alcohol	Complex profile*
**Carnivores**	183	61.7%	0%	24.6%	24.6%	0%	9.8%
**Herbivores**	15	33.3%	0%	0%	66.7%	0%	6.7%
**Omnivores**	17	11.8%	0%	0%	88.2%	0%	5.9%

*Complex profile defined as: (1) bile salt class II, III, or V; and/or (2) presence of 3 or more bile salts in biliary bile, each at 10% or more

Abbreviations and bile salt names used:

CA (cholic acid; 3α,7α,12α-trihydroxy-5β-cholan-24-oic acid) – common C_{24} bile acid

CDCA (chenodeoxycholic acid; 3α,7α-dihydroxy-5β-cholan-24-oic acid) – common stem C_{24} bile acid

DCA (deoxycholic acid; 3α,12α-dihydroxy-5β-cholan-24-oic acid) – common secondary C_{24} bile acid

Allo- - 5α-bile salt (e.g., alloCA or alloCDCA)

LCA (lithocholic acid; 3α-hydroxy-5β-cholan-24-oic acid) - common (often toxic) secondary C_{24} bile acid

PythoCA (pythocholic acid; 3α,12α,16α-5β-cholan-24-oic acid) – unusual C_{24} bile acid lacking 7α-OH; found in some snakes

SteroCA (sterocholic acid; 3α,7α,12α,22-tetrahydroxy-5β-cholestan-27-oic acid) – 22-hydroxylated C_{27} bile acid found in turtles

SteroCDCA (sterochenodeoxycholic acid; 3α,7α,22-trihydroxy-5β-cholestan-27-oic acid) –C_{27} bile acid found in turtles

Varanic acid (3α,7α,12α,24*R*-tetrahydroxy-5β-cholestan-27-oic acid) – 24*R*-hydroxylated C_{27} bile acid typical of varanid lizards

**Species**		**Bile salt class**	**5H**	**C_{27} alcohols**	**C_{27} acids**	**C_{24} acids**	**Conjugation**	**Major bile salts**	**Methodology**	**Comments**
**REPTILES**										
**TESTUDINES**										All turtles analyzed use C_{27} bile acids conjugated with taurine and use rather unique 22-hydroxylation (sterochenodeoxycholic and sterocholic acids)
**Carettochelyididae**										
Carettochelys insculpta	Plateless turtle	IV	5β		X		Taurine	Very complex mixture of C_{27} bile acids	HPLC	Very low levels of 12α-hydroxylation
**Chelidae**										
Chelodina novaeguineae	New Guinea snake-neck turtle	IV	5β		X		Taurine	SteroCA, steroCDCA	HPLC	
Chelodina siebenrocki	Siebenrock's snake-neck turtle	II	5β	X	X	X	Taurine	Complex mixture of C_{27} bile acids (70%), C_{27} bile alcohols (22%), and C_{24} bile acids (8%)	ESI/MS/MS	
Chelus fimbriatus	Mata Mata turtle	IV	5β		X		Taurine	SteroCA, steroCDCA	HPLC	
**Cheloniidae**										
Caretta caretta	Loggerhead turtle	IV	5β		X		Taurine	SteroCA, steroCDCA, steroDCA	HPLC	Approximately 10% unidentified bile acids likely representing complex stereoisomers
Chelonia midas	Green turtle	IV	5β		X		Taurine	SteroCA, steroCDCA, steroDCA	HPLC	Approximately 10% unidentified bile acids likely representing complex stereoisomers
Eretmochelys imbricata	Hawks bill turtle	IV	5β		X		Taurine	SteroCA, steroCDCA, steroDCA	HPLC	Approximately 6% unidentified bile acids likely representing complex stereoisomers
Lepidochelys olivacea	Olive Ridley turtle	IV	5β		X		Taurine	SteroCA, steroCDCA, steroDCA	HPLC	Approximately 20% unidentified bile acids likely representing complex stereoisomers

Additional file 1 (cont.), p. 3

Species		Bile salt class	5H	C_{27} alcohols	C_{27} acids	C_{24} acids	Conjugation	Major bile salts	Methodology	Comments
**Chelydridae**										
Macrochelys temminckii	Alligator snapping turtle	IV	5β		X		Taurine	SteroCA, steroCDCA, steroDCA	HPLC	SteroDCA may result from enterohepatic circulation
**Dermochelyidae**										
Dermochelys coriacea	Leatherback turtle	IV	5β		X		Taurine	SteroCDCA, steroCA	HPLC	Approximately 40% unidentified complex mixture of C_{27} bile acids
**Emydidae**										15α-Hydroxylated C_{27} bile acids also found in this family.
Chrysemys picta	Painted turtle	IV	5β		X		Taurine	SteroCA, steroCDCA	HPLC	
Glyptemys insculpta	Wood turtle	IV	5β		X		Taurine	SteroCA, steroCDCA	HPLC	
Graptemys geographica	Map turtle	IV	5β		X		Taurine	SteroCA, steroCDCA	HPLC	
Malaclemys terrapin	Diamondback terrapin	IV	5β		X		Taurine	SteroCA, steroCDCA	HPLC	
Pseudemys concinna	Common cooter	IV	5β		X		Taurine	SteroCA, steroCDCA	HPLC	
Trachemys scripta	Red-eared turtle	IV	5β		X		Taurine	SteroCA, steroCDCA	HPLC	
**Geoemydidae**										
Chinemys reevesii	Reeves turtle	IV	5β		X		Taurine	SteroCA, steroCDCA	HPLC	
Cuoro galbinifrons	Indochinese box turtle	IV	5β		X		Taurine	SteroCA, steroCDCA	HPLC	
Cuora trifasciata	Chinese three-striped turtle	IV	5β		X		Taurine	SteroCA, steroCDCA	HPLC	
Cyclemys tcheponensis	Stripe-necked leaf turtle	IV	5β		X		Taurine	SteroCA, steroCDCA	HPLC	
**Podocnemidae**										
Podocnemis expansa	South American river turtle	IV	5β		X		Taurine	SteroCA, steroCDCA	HPLC	

Species		Bile salt class	5H	C_{27} alcohols	C_{27} acids	C_{24} acids	Conjugation	Major bile salts	Methodology	Comments
**Testudinidae**										
Chelonoidis denticulata	Yellow-footed tortoise	IV	5β		X		Taurine	25*R* and 25*S* substituted steroCA and steroCDCA	HPLC	
Geochelone nigra	Galapagas tortoise	IV	5β		X		Taurine	25*R* and 25*S* substituted steroCA and steroCDCA	HPLC	
Geochelone sulcata	African spurred tortoise	IV	5β		X		Taurine	25*R* and 25*S* substituted steroCA and steroCDCA	HPLC	
Gopherus agassizii	California desert tortoise	II	5β	X	X		Taurine, sulfate	C_{27} bile acids; C_{26}, C_{27}, and C_{28} bile alcohol sulfates (30% of total bile salts)	ESI/MS/MS	Highest bile alcohol sulfate content in biliary bile for any reptile examined. C_{26} and C_{28} bile alcohol sulfates are very rare in vertebrates other than amphibians.
Manouria emys	Asian brown tortoise	IV	5β		X		Taurine	25*R* and 25*S* substituted steroCA and steroCDCA	HPLC	
Manouria impressa	Impressed tortoise	IV	5β		X		Taurine	25*R* and 25*S* substituted steroCA and steroCDCA	HPLC	
Psammobates pardalis	Leopard tortoise	V	5β		X	X	Taurine	CA, CDCA, 25*R* and 25*S* substituted steroCA and steroCDCA	LC/MS	
Pyxis arachnoides	Madagascar spider tortoise	V	5β		X	X	Taurine	CA, CDCA, C_{27} bile acids	LC/MS	
Testudo horsfieldii	Central Asian tortoise	IV	5β		X		Taurine	25*R* and 25*S* substituted steroCA and steroCDCA	HPLC	
**Trionychidae**										
Chitra indica	Narrow-head soft shell turtle	IV	5β		X		Taurine	SteroCA	HPLC	
Pelochelys bibronii	Bibron's soft shell turtle	IV	5β		X		Taurine	SteroCA	HPLC	
Pelodiscus sinensis	Chinese soft shell turtle	IV	5β		X		Taurine	SteroCA	HPLC	

Species		Bile salt class	5H	C_{27} alcohols	C_{27} acids	C_{24} acids	Conjugation	Major bile salts	Methodology	Comments
**SPHENODONTIA**										
Sphenodon punctatus	Tuatara	IV	5β		X		Taurine	24*R*-hydroxylated C_{27} bile acids	HPLC	
**LIZARDS, GECKOES, CHAMELEONS**										
**Agamidae**										
Agama agama	Red-headed rock agama	VI	5α			X	Taurine	AlloCA	HPLC	
Chlamydosaurus kingii	Frilled lizard	VI	5α / 5β			X	Taurine	AlloCA, 25% CA	HPLC	
Laudakia stellio	Starred agama	VI	5α			X	Taurine	AlloCA	HPLC	
Physignathus cocincinus	Chinese water dragon	VI	5α			X	Taurine	AlloCA, CA	HPLC	
Physignathus lesueurii	Leseur's water dragon	VI	5α			X	Taurine	AlloCA, CA	HPLC	
Pogona barbata	Eastern bearded dragon	VI	5α			X	Taurine	AlloCA, CA	HPLC	
Pogona vitticeps	Inland bearded dragon	VI	5α			X	Taurine	AlloCA, CA	HPLC, GC/MS	
Uromastyx acanthinura	North African spiny-tailed lizard	VI	5α			X	Taurine	Mostly alloCA, also alloDCA	HPLC	
Uromastyx aegyptia microlepis	Egyptian spiny-tailed lizard	VI	5α			X	Taurine	Mostly alloCA, also alloDCA	HPLC	
Uromastyx benti	Benti's spiny-tailed lizard	VI	5α			X	Taurine	Mostly alloCA, also alloDCA	HPLC	
Uromastyx hardwicki	Indian spiny-tailed lizard	VI	5α			X	Taurine	Mostly alloCA, also alloDCA	HPLC	
Uromastyx ocellata	Ornate spiny-tailed lizard	VI	5α			X	Taurine	Mostly alloCA, also alloDCA	HPLC	
Uromastyx thomasi	Thomas' spiny-tailed lizard	VI	5α			X	Taurine	Mostly alloCA, also alloDCA	HPLC	

Species		Bile salt class	5H	C_{27} alcohols	C_{27} acids	C_{24} acids	Conjugation	Major bile salts	Methodology	Comments
**Anguidae**										
Elgaria multicarinata	California alligator lizard	V	5β		X	X	Taurine	Varanic acid, 6% C_{24} bile acids	HPLC	
Pseudopus apodus	Scheltopusik glass lizard	V	5β		X	X	Taurine	Varanic acid, 8% C_{24} bile acids	HPLC	
**Chamaeleonidae**										
Brookesia perarmata	Armored chameleon	VI	5α			X	Taurine	Mostly alloCA	HPLC	
Calumma parsonii	Parson's chameleon	VI	5α			X	Taurine	Mostly alloCA	HPLC	
Chamaeleo calyptratus	Veiled chameleon	VI	5α			X	Taurine	Mostly alloCA	HPLC	
Chamaeleo gracilis	Graceful chameleon	VI	5α			X	Taurine	Mostly alloCA	HPLC	
Chamaeleo jacksonii	Jackson's chameleon	VI	5α			X	Taurine	Mostly alloCA	HPLC	
Chamaeleo melleri	Meller's chameleon	VI	5α			X	Taurine	Mostly alloCA	HPLC	
Chamaeleo montium	Sailfin chameleon	VI	5α			X	Taurine	Mostly alloCA	HPLC	
Chamaeleo quadricornis	Four-horned chameleon	VI	5α			X	Taurine	Mostly alloCA	HPLC	
Furcifer oustaleti	Oustalet's chameleon	VI	5α			X	Taurine	Mostly alloCA	HPLC	
Furcifer pardalis	Panther chameleon	VI	5α			X	Taurine	Mostly alloCA	HPLC	
Rampholeon spectrum	Leaf chameleon	VI	5α			X	Taurine	Mostly alloCA	HPLC	
Rieppeleon brevicaudatus	Stump-tail chameleon	VI	5α			X	Taurine	Mostly alloCA	HPLC	
**Cordylidae**										
Cordylus warreni	Warren's girdle-tail	VI	5α			X	Taurine	alloCA + complex mixture of other C_{24} bile acids	HPLC	

Species		Bile salt class	5H	C_{27} alcohols	C_{27} acids	C_{24} acids	Conjugation	Major bile salts	Methodology	Comments
**Gekkonidae**										
Cyrtodactylus pulchellus	Malayan bent-toed gecko	VI	5β			X	Taurine	CA, CDCA	HPLC	
Gekko gecko	Tokay gecko	VI	5α			X	Taurine	alloCA, alloCDCA	HPLC	
Hemitheconyx caudicinctus	Fat-tailed gecko	VI	5β / 5α			X	Taurine	CA, CDCA	HPLC	
Phelsuma flavigularis	Yellow-throated day gecko	VI	5β			X	Taurine	CA, CDCA	HPLC	
Ptychozoon kuhli	Gliding gecko	VI	5β / 5α			X	Taurine	CA, CDCA, alloCA	HPLC	
**Helodermatidae**										
Heloderma suspectum	Gila monster	IV	5β		X		Taurine	Mostly varanic acid	HPLC	
**Iguanidae**										
Anolis caroliensis	Green anole	VI	5α / 5β			X	Taurine	Mostly alloCA	HPLC	
Anolis equestris	Knight anole	VI	5α / 5β			X	Taurine	Equal mixture of alloCA and CA	HPLC	
Anolis grahami	Jamaican anole	VI	5α / 5β			X	Taurine	Mostly alloCA, only 6% 5β bile acids	HPLC	
Brachylophus fasciatus	Hybrid Fiji Island green iguana	VI	5α / 5β			X	Taurine	Mostly alloCA	HPLC	
Corytophanes cristatus	Casque-headed iguana	VI	5α / 5β			X	Taurine	AlloCA, alloCDCA, only 15% 5β bile acids	HPLC	
Cyclura carinata	Turks and Caicos iguana	VI	5α / 5β			X	Taurine	Mostly alloCA, only 8% 5β bile acids	HPLC	
Cyclura cornuta cornuta	Rhinoceros iguana	VI	5α / 5β			X	Taurine	Mostly alloCA	HPLC	
Sauromalus ater	Chuckwalla	VI	5α / 5β			X	Taurine	Mostly alloCA, only 8% 5β bile acids	HPLC	
**Lacertidae**										

Species		Bile salt class	5H	C_{27} alcohols	C_{27} acids	C_{24} acids	Conjugation	Major bile salts	Methodology	Comments
Lacerta viridis	Emeralda lacerta	VI	5β / 5α			X	Taurine	Mostly CA, some alloCA	HPLC	
**Scincidae**										
Egernia depressa	Pygmy spiny-tailed skink	IV	5β		X		Taurine	>97% C_{27} bile acids	HPLC	
Trachylepis affinis	Blotched blue-tailed skink	V	5β		X	X	Taurine	C_{27} bile acids, ~35% C_{24} bile acids	ESI/MS/MS	Also traces of unusual C_{27} bile alcohol sulfates
**Teiidae**										
Ameiva ameiva	Blue ameiva	VI	5β			X	Taurine	Mostly CA	HPLC	
Cnemidophorus vanzoi	St. Lucia whiptail	VI	5β			X	Taurine	Mostly CA	HPLC	
Dracaena guianensis	Caiman lizard	VI	5β			X	Taurine	Mostly CA	HPLC	
Tupinambis sp.	Tegu	VI	5β			X	Taurine	Mostly CA	HPLC	
**Varanidae**										
Varanus albigularis	White-throated monitor	IV	5β		X		Taurine	Mostly varanic acid	HPLC	
Varanus beccarii	Black-tree monitor	IV	5β		X		Taurine	Mostly varanic acid	HPLC	
Varanus bengalensis	Bengal monitor	IV	5β		X		Taurine	Mostly varanic acid	HPLC, ESI/MS/MS	
Varanus exanthematicus	Savannah monitor	IV	5β		X		Taurine	Mostly varanic acid	HPLC	
Varanus exanthematicus	Savannah monitor	IV	5β		X		Taurine	Mostly varanic acid	HPLC	
Varanus gouldi	Gould's monitor	IV	5β		X		Taurine	Mostly varanic acid	HPLC	
Varanus indicus	Mangroe monitor	IV	5β		X		Taurine	Mostly varanic acid	HPLC	
Varanus jobiensis	Peach-throated monitor	IV	5β		X		Taurine	Mostly varanic acid	HPLC	
Varanus komodoensis	Komodo monitor	IV	5β		X		Taurine	Mostly varanic acid	HPLC	
Varanus	Emerald	IV	5β		X		Taurine	Mostly varanic acid	HPLC	

Additional file 1 (cont.), p. 9

Species		Bile salt class	5H	C_{27} alcohols	C_{27} acids	C_{24} acids	Conjugation	Major bile salts	Methodology	Comments
kordensis	monitor									
Varanus melinus	Quince monitor	IV	5β		X		Taurine	Mostly varanic acid	HPLC	
Varanus niloticus	Nile monitor	IV	5β		X		Taurine	Mostly varanic acid	HPLC	
Varanus olivaceus	Gray's monitor	IV	5β		X		Taurine	Mostly varanic acid	HPLC	
Varanus panoptes	New Guinea monitor	IV	5β		X		Taurine	Mostly varanic acid	HPLC	
Varanus rudicollis	Rough-neck monitor	IV	5β		X		Taurine	Mostly varanic acid	HPLC	
Varanus salvator	Two-banded monitor	IV	5β		X		Taurine	Mostly varanic acid	HPLC	
Varanus timorensis	Timor monitor	IV	5β		X		Taurine	Mostly varanic acid	HPLC	
Varanus varius	Lace monitor	IV	5β		X		Taurine	Mostly varanic acid	HPLC	
**SNAKES**										
**Acrochordidae**										
Acrocordus javanicus	Elephant trunk snake	VI	5β			X	Taurine	CA	HPLC	Acrochordids no longer show 16α-hydroxylation
Acrocordus granulates	File snake	VI	5β / 5α			X	Taurine	CA, DCA, alloCA	HPLC	
**Boidae**										
Acrantophis madagascariensis	Madagascar ground boa	VI	5β			X	Taurine	Mostly pythoCA	HPLC	
Boa constrictor imperator	Red-tail boa	VI	5β			X	Taurine	Mostly pythoCA	HPLC	
Boa constrictor occidentalis	Argentine boa	VI	5β			X	Taurine	Mostly pythoCA	HPLC	
Boa constrictor ortonii	Red-tailed boa	VI	5β			X	Taurine	Mostly pythoCA	HPLC	
Calabaria reinhardtii	Calabar ground python	VI	5β			X	Taurine	Mostly pythoCA	HPLC	
Candoia bibroni australis	Solomon Islands boa	VI	5β			X	Taurine	Mostly pythoCA	HPLC	

Additional file 1 (cont.), p. 10

Species		Bile salt class	5H	C_{27} alcohols	C_{27} acids	C_{24} acids	Conjugation	Major bile salts	Methodology	Comments
Corallus caninus	Emerald tree boa	VI	5β			X	Taurine	Mostly pythoCA	HPLC	
Eryx johnii	Brown sand boa	VI	5β			X	Taurine	Mostly pythoCA	HPLC	
Lichanura australis roseofusca	Coastal rosy boa	VI	5β			X	Taurine	Mostly pythoCA	HPLC	
**Colubridae**										
Ahaetulla nasuta	Green tree snake	VI	5β / 5α			X	Taurine	Mostly CA, 25% alloCA	HPLC	AlloCA may be from diet
Dispholidus typhus	Boomslang	VI	5β			X	Taurine	Mostly CA	HPLC	
Drymarchon corais	Indigo snake	VI	5β			X	Taurine	Mostly CA	HPLC	
Elaphe quatuorlineata	Four-lined ratsnake	VI	5β			X	Taurine	Mostly CA	HPLC	
Euprepiophis mandarina	Mandarin ratsnake	VI	5β			X	Taurine	Mostly CA	HPLC	
Gongylosoma longicauda	Striped gopher snake	VI	5β			X	Taurine	Mostly CA, minor fraction of Δ22 and 23*R*-OH bile acids	HPLC	
Lampropeltis getulus	Speckled kingsnake	VI	5β			X	Taurine	Mostly CA, minor fraction of Δ22 bile acids	HPLC	
Lampropeltis triangulum	Milksnake	VI	5β			X	Taurine	Mostly CA, minor fraction of Δ22 bile acids	HPLC	
Macrocalamus lateralis	Malayan mountain reed snake	VI	5β			X	Taurine	Mostly CA	HPLC	Minor fractions of C_{27} bile alcohol sulfates identified
Masticophis flagellum testaceus	Western coachwhip	VI	5β			X	Taurine	Mostly CA	HPLC	
Masticophis taeniatus	Striped whipsnake	VI	5β			X	Taurine	Mostly CA	HPLC	
Orthriophis moellendorfi	Red-head ratsnake	VI	5β			X	Taurine	Mostly CA	HPLC	

Species		Bile salt class	5H	C_{27} alcohols	C_{27} acids	C_{24} acids	Conjugation	Major bile salts	Methodology	Comments
Pituophis deppei jani	Queretaro gopher snake	VI	5β			X	Taurine	Mostly CA	HPLC	
Ptyas mucosus	Dhaman	VI	5β			X	Taurine	Mostly CA	HPLC	
Stegonotus modestus	Northern ground snake	VI	5β			X	Taurine	Mostly CA	HPLC	
Telescopus semiannulatus	African tiger snake	VI	5β			X	Taurine	Mostly CA	HPLC	
Thelotornis kirtlandii	Twig snake	VI	5β			X	Taurine	Mostly CA	HPLC	
Zamenis longissimus	Aesculapian snake	VI	5β			X	Taurine	Mostly CA	HPLC	
**Cylindrophiidae**										
Cylindrophis maculatus	Blotched pipe snake	VI	5β			X	Taurine	PythoCA, 7α-OH-pythoCA	HPLC	
Cylindrophis rufus	Pipe snake	VI	5β			X	Taurine	PythoCA, 7α-OH-pythoCA	HPLC	
**Dipsadidae**										
Farancia abacura	Mudsnake	VI	5β			X	Taurine	23*R*-OH-CA, CA, and DCA	HPLC	
**Elapidae**										
Acanthophis antarcticus	Death adder	VI	5β			X	Taurine	Mostly CA	HPLC	
Bungarus fasciatus	Banded krait	VI	5β / 5α			X	Taurine	CA, minor fraction of alloCA	HPLC	
Bungarus flaviceps	Red-head trait	VI	5β / 5α			X	Taurine	CA, minor fraction of alloCA	HPLC	
Calliophis bivirgata	Blue Malaysian coral snake	VI	5β / 5α			X	Taurine	CA, minor fraction of alloCA	HPLC	
Dendroaspis polylepis	South African mamba	VI	5β			X	Taurine	Mixture of C_{24} and C_{23} bile acids, some with 23*R*-OH	HPLC	
Laticauda colubrine	Yellow-lipped sea snake	VI	5β			X	Taurine	Mostly CA	HPLC	

**Species**		**Bile salt class**	**5H**	**C_{27} alcohols**	**C_{27} acids**	**C_{24} acids**	**Conjugation**	**Major bile salts**	**Methodology**	**Comments**
Laticauda semifasciata	Erabu sea snake	VI	5β			X	Taurine	Mostly CA	HPLC	
Naja haje	Egyptian banded cobra	VI	5β / 5α			X	Taurine	Mostly CA	HPLC, LC/MS/MS	
Naja pallida	Red-spitting cobra	VI	5β / 5α			X	Taurine	CA, minor fraction of alloCA	HPLC	
Notechis scutatus	Australian tiger snake	VI	5β			X	Taurine	Mostly CA	HPLC	
Ophiophagus hannah	King cobra	VI	5β / 5α			X	Taurine	CA, minor fraction of alloCA	HPLC	
Pelamis platurus	Yellow-bellied sea snake	VI	5β			X	Taurine	Mostly CA	HPLC	
Pseudechis colletti	Collett's black snake	VI	5β			X	Taurine	Mostly CA	HPLC	
**Homalopsidae**										
Enhydris plumbea	Plumbeous watersnake	VI	5β			X	Taurine	23*R*-OH-CA, CA	HPLC	
Homalopsis buccata	Puff-faced watersnake	VI	5β			X	Taurine	23*R*-OH-CA, CA	HPLC	
**Lamprophiidae**										
Lycodonomorphus rufulus	Red wolf snake	VI	5β			X	Taurine	Mostly CA	HPLC	
Psammophis condanarus	Indo-Chinese sand snake	VI	5β			X	Taurine	Mostly CA	HPLC	
Pseudaspis cana	Mole snake	VI	5β / 5α			X	Taurine	CA, minor fraction of alloCA	HPLC	
**Natricidae**										
Nerodia erythrogaster	Plain-bellied water snake	VI	5β			X	Taurine	Mostly CA	HPLC	
Nerodia fasciata fasciata	Banded water snake	VI	5β			X	Taurine	Mostly CA	HPLC	
Opisthotropis lateralis	Bicolored stream snake	VI	5β			X	Taurine	Mostly CA	HPLC	
Storeria occipitomaculata	Red-bellied	VI	5β			X	Taurine	Mostly CA	HPLC	

Species		Bile salt class	5H	C_{27} alcohols	C_{27} acids	C_{24} acids	Conjugation	Major bile salts	Methodology	Comments
	snake									
**Pareatidae**										
Pareas margaritophorus	White-spotted slug snake	VI	5β			X	Taurine	Mostly CA	HPLC	
Asthenodipsas vertebralis	Mountain slug snake	VI	5β			X	Taurine	Mostly CA	HPLC	
**Pythonidae**										
Aspidites melanocephalus	Black-headed python	VI	5β			X	Taurine	Mostly pythoCA	HPLC	
Aspidites ramsayi	Woma python	VI	5β			X	Taurine	PythoCA, CA	HPLC	Shows pythoCA to CA transition
Liasis albertsii	D'Albert's python	VI	5β			X	Taurine	Mostly pythoCA	HPLC	
Morelia boeleni	Boehlin's python	VI	5β			X	Taurine	Mostly pythoCA	HPLC	
Morelia spilota variegata	Carpet python	VI	5β			X	Taurine	Mostly pythoCA	HPLC	
Morelia spilata	Diamond python	VI	5β			X	Taurine	Mostly pythoCA	HPLC	
Morelia viridis	Green tree python	VI	5β			X	Taurine	Mostly pythoCA	HPLC	
Python bivittatus	Burmese python	VI	5β			X	Taurine	Mostly pythoCA	HPLC	
Python curtus	Blood python	VI	5β			X	Taurine	Mostly pythoCA	HPLC	
Python molurus molurus	Indian rock python	VI	5β			X	Taurine	Mostly pythoCA	HPLC	
Python regius	Ball python	VI	5β			X	Taurine	Mostly pythoCA	HPLC	
Pytho sebae	African rock python	VI	5β			X	Taurine	Mostly pythoCA	HPLC	
**Uropeltidae**										
Rhinophis sp.	Shield-tail snake	VI	5β			X	Taurine	PythoCA, CA	HPLC	
**Viperidae**										

Additional file 1 (cont.), p. 14

Species		Bile salt class	5H	C_{27} alcohols	C_{27} acids	C_{24} acids	Conjugation	Major bile salts	Methodology	Comments
Agkistrodon contortix contortix	Copperhead	VI	5β			X	Taurine	CA	HPLC	
Agkistrodon contortix pictigaster	Trans-Peco copperhead	VI	5β			X	Taurine	CA	HPLC	
Azemiops feae	White-headed viper	VI	5β			X	Taurine	CA	HPLC	
Bitis arietans	Puff adder	VI	5β			X	Taurine	CA, CDCA	HPLC	
Bitis atropos	Mountain adder	VI	5β			X	Taurine	Mixture of C_{24} and C_{23} bile acids, some with 23*R*-OH	HPLC	
Bitis cornuta	Eastern homsman adder	VI	5β			X	Taurine	Mixture of C_{24} and C_{23} bile acids, some with 23*R*-OH	HPLC	
Bitis gabonica	Gaboon viper	VI	5β			X	Taurine	Mixture of C_{24} and C_{23} bile acids, some with 23*R*-OH	HPLC	
Bitis nasicornis	Rhinoceros viper	VI	5β			X	Taurine	Mixture of C_{24} and C_{23} bile acids, some with 23*R*-OH	HPLC	
Bothriechis schlegelii	Eyelash viper	VI	5β			X	Taurine	CA	HPLC	
Bothrops alternates	Urutu	VI	5β			X	Taurine	CA	HPLC	
Bothrops jaracara	Jaracara	VI	5β			X	Taurine	CA	HPLC	
Bothrops neuwiedi	Neuwied's lancehead	VI	5β			X	Taurine	CA	HPLC	
Cerastes cerastes	Homed sand viper	VI	5β			X	Taurine	Mixture of C_{24} and C_{23} bile acids, some with 23*R*-OH	HPLC	
Crotalus adamanteus	Eastern diamondback	VI	5β			X	Taurine	Mostly CA	HPLC	Small amounts of 23*R*-hydroxylated and Δ22 bile acids
Crotalus catalinensis	Santa Cantalina	VI	5β			X	Taurine	Mostly CA	HPLC	Small amounts of 23*R*-hydroxylated and Δ22 bile acids
Crotalus cerastes	Sidewinder	VI	5β			X	Taurine	Mostly CA	HPLC	Small amounts of 23*R*-hydroxylated and Δ22 bile acids

Additional file 1 (cont.), p. 15

Species		Bile salt class	5H	C_{27} alcohols	C_{27} acids	C_{24} acids	Conjugation	Major bile salts	Methodology	Comments
Crotalus durissus	South American rattlesnake	VI	5β			X	Taurine	Mostly CA	HPLC	Small amounts of 23*R*-hydroxylated and Δ22 bile acids
Crotalus helleri	South Pacific rattlesnake	VI	5β			X	Taurine	Mostly CA	HPLC	Small amounts of 23*R*-hydroxylated and Δ22 bile acids
Crotalus lepidus klauberi	Banded rock rattlesnake	VI	5β			X	Taurine	Mostly CA	HPLC	Small amounts of 23*R*-hydroxylated and Δ22 bile acids
Crotalus ruber	Red diamond rattlesnake	VI	5β			X	Taurine	Mostly CA	HPLC	Small amounts of 23*R*-hydroxylated and Δ22 bile acids
Crotalus scutulatus	Mojave green rattlesnake	VI	5β			X	Taurine	Mostly CA	HPLC	Small amounts of 23*R*-hydroxylated and Δ22 bile acids
Crotalus unicolor	Arubu Island rattlesnake	VI	5β			X	Taurine	Mostly CA	HPLC	Small amounts of 23*R*-hydroxylated and Δ22 bile acids
Crotalus vegrandis	Uracoan rattlesnake	VI	5β			X	Taurine	Mostly CA	HPLC	Small amounts of 23*R*-hydroxylated and Δ22 bile acids
Daboia russelli	Russell's viper	VI	5β			X	Taurine	Mixture of C_{24} and C_{23} bile acids, some with 23*R*-OH	HPLC	
Deinagkistrodon acutus	Snub-nosed pit viper	VI	5β			X	Taurine	Mostly CA	HPLC	Small amounts of 23*R*-hydroxylated and Δ22 bile acids
Eristicophis macmahoni	Leaf-nosed viper	VI	5β			X	Taurine	Mixture of C_{24} and C_{23} bile acids, some with 23*R*-OH	HPLC	
Lachesis muta	Bushmaster	VI	5β			X	Taurine	Mostly CA	HPLC	Small amounts of 23*R*-hydroxylated and Δ22 bile acids
Macrovipera lebetina	Turan blunt-nosed viper	VI	5β			X	Taurine	Mixture of C_{24} and C_{23} bile acids, some with 23*R*-OH	HPLC	
Parias flavomaculatus	Philippine viper	VI	5β			X	Taurine	Mostly CA	HPLC	Small amounts of 23*R*-hydroxylated and Δ22 bile acids
Parias mcgregori	McGregor's tree viper	VI	5β			X	Taurine	Mostly CA	HPLC	Small amounts of 23*R*-hydroxylated and Δ22 bile acids
Trimeresurus trigonocephalus	Sri Lanka viper	VI	5β			X	Taurine	Mostly CA	HPLC	Small amounts of 23*R*-hydroxylated and Δ22 bile acids
Tropidolaemus wagleri	Wagler's viper	VI	5β			X	Taurine	Mostly CA	HPLC	Small amounts of 23*R*-hydroxylated and Δ22 bile acids
**CROCODYLIA**										

Species		Bile salt class	5H	C_{27} alcohols	C_{27} acids	C_{24} acids	Conjugation	Major bile salts	Methodology	Comments
**Alligatoridae**										
Alligator mississipiensis	American alligator	IV	5β		X		Taurine	Mostly 3α,7α,12α-trihydroxy-5β-cholestan-27-oic acid	HPLC	
Alligator sinensis	Chinese alligator	IV	5β		X		Taurine	Mostly 3α,7α,12α-trihydroxy-5β-cholestan-27-oic acid	HPLC	
Caiman crocodilus	Spectacled caiman	IV	5β		X		Taurine	Mostly 3α,7α,12α-trihydroxy-5β-cholestan-27-oic acid	HPLC	
Caiman latirostris	Broad-nosed caiman	IV	5β		X		Taurine	Mostly 3α,7α,12α-trihydroxy-5β-cholestan-27-oic acid	HPLC	
Melanosuchus niger	Black caiman	IV	5β		X		Taurine	Mostly 3α,7α,12α-trihydroxy-5β-cholestan-27-oic acid	HPLC	
Paleosuchus palpebrosus	Dwarf caiman	IV	5β		X		Taurine	Mostly 3α,7α,12α-trihydroxy-5β-cholestan-27-oic acid	HPLC	
**Crocodylidae**										
Crocodylus acutus	American crocodile	IV	5β		X		Taurine	Mostly 3α,7α,12α-trihydroxy-5β-cholestan-27-oic acid	HPLC	
Crocodylus intermedius	Orinoco crocodile	IV	5β		X		Taurine	Mostly 3α,7α,12α-trihydroxy-5β-cholestan-27-oic acid	HPLC	
Crocodylus johnsoni	Johnson's crocodile	IV	5β		X		Taurine	Mostly 3α,7α,12α-trihydroxy-5β-cholestan-27-oic acid	HPLC	
Crocodylus niloticus	Nile crocodile	IV	5β		X		Taurine	Mostly 3α,7α,12α-trihydroxy-5β-cholestan-27-oic acid	HPLC	
Crocodylus novaeguineae	New Guinea crocodile	IV	5β		X		Taurine	Mostly 3α,7α,12α-trihydroxy-5β-cholestan-27-oic acid	HPLC	
Crocodylus palustris	Mugger	IV	5β		X		Taurine	Mostly 3α,7α,12α-trihydroxy-5β-	HPLC	

Species		Bile salt class	5H	C_{27} alcohols	C_{27} acids	C_{24} acids	Conjugation	Major bile salts	Methodology	Comments
								cholestan-27-oic acid		
Crocodylus porosus	Australian saltwater crocodile	IV	5β		X		Taurine	Mostly 3α,7α,12α-trihydroxy-5β-cholestan-27-oic acid	HPLC	
Crocodylus siamensis	Siamese crocodile	IV	5β		X		Taurine	Mostly 3α,7α,12α-trihydroxy-5β-cholestan-27-oic acid	HPLC	
Osteolaemus tetraspis	Dwarf crocodile	IV	5β		X		Taurine	Mostly 3α,7α,12α-trihydroxy-5β-cholestan-27-oic acid	HPLC	
**Gavialidae**										
Gavialis gangeticus	Indian gavial	IV	5β		X		Taurine	Mostly 3α,7α,12α-trihydroxy-5β-cholestan-27-oic acid	HPLC	
Tomistoma schlegelii	False gavial	IV	5β		X		Taurine	Mostly 3α,7α,12α-trihydroxy-5β-cholestan-27-oic acid	HPLC	
