# Supplementary material for: Evolutionary diversity of bile salts in reptiles and mammals, including analysis of ancient human and extinct giant ground sloth coprolites
Source: BMC Evol Biol. 2010 May 6;10:133. doi: 10.1186/1471-2148-10-133 (PMC2886068; doi:10.1186/1471-2148-10-133)
Supplement: Additional file 2 — Bile salts of mammals. Table contains data on the bile salts of all mammals analyzed, with bile salt profiles color-coded by bile salt class. [file 1471-2148-10-133-S2.PDF]

Additional file 2: Bile salts of mammals

Summary of detailed categorization by bile salt class

Class	Description	Number of mammalian species
I	**Only C_{27} bile alcohols**	10 (elephants, rock hyrax, manatees, rhinoceroses)
II	**Mixture of C_{27} bile alcohols and C_{27} bile acids**	0
III	**Mixture of C_{27} bile alcohols and C_{24} bile acids**	5 (hutias, Cervidae)
IV	**Only C_{27} bile acids**	0
V	**Mixture of C_{27} bile acids and C_{24} bile acids**	11
VI	**All C_{24} bile acids**	300
	**Total**	**326**
	**Presence of glycine-conjugated bile acids at 5% or greater**	116
	**Mostly 5α (A/B *trans*, 'allo')**	1 (fox squirrel)

Summary of diet class sorted by primary bile salt

Diet class	Total #	CA	CDCA	Other C_{24} bile acid	C_{27} bile acid	C_{27} bile alcohol	Complex profile*
**Carnivores**	103	80.6%	4.9%	13.6%	0%	0%	39.8%
**Herbivores**	149	77.2%	9.4%	7.4%	0%	6.0%	70.5%
**Omnivores**	74	74.3%	12.2%	12.2%	0%	1.4%	50.0%

*Complex profile defined as: (1) bile salt class II, III, or V; and/or (2) presence of 3 or more bile salts in biliary bile, each at 10% or more

Abbreviations and bile salt names used:

CA (cholic acid; 3α,7α,12α-trihydroxy-5β-cholan-24-oic acid) – common C_{24} bile acid
CDCA (chenodeoxycholic acid; 3α,7α-dihydroxy-5β-cholan-24-oic acid) – common stem C_{24} bile acid
Allo- - 5α-bile salt (e.g., alloCA or alloCDCA)
DCA (deoxycholic acid; 3α,12α-dihydroxy-5β-cholan-24-oic acid) – common secondary C_{24} bile acid
HCA (hyocholic acid; 3α,6α,7α-trihydroxy-5β-cholan-24-oic acid) – C_{24} bile acid found in Suidae
HDCA (hyodeoxycholic acid; 3α,6α-dihydroxy-5β-cholan-24-oic acid) – C_{24} bile acid found in Suidae
LCA (lithocholic acid; 3α-hydroxy-5β-cholan-24-oic acid) - common (often toxic) secondary C_{24} bile acid
α-MCA (α-muricholic acid; 3α,6β,7α-trihydroxy-5β-cholan-24-oic acid) – C_{24} bile acid found in Rodentia
β-MCA (β-muricholic acid; 3α,6β,7β-trihydroxy-5β-cholan-24-oic acid) – C_{24} bile acid found in Rodentia
UDCA (ursodeoxycholic acid; 3α,7β-dihydroxy-5β-cholan-24-oic acid) – C_{24} bile acid found in Ursidae

Additional file 2, p. 2

Species		Bile salt class	5H	C_{27} alcohols	C_{27} acids	C_{24} acids	Conjugation	Major bile salts	Methodology	Comments
**MAMMALIA**										
**PROTOTHERIA**										
Ornithorhynchus anatinus	Duck-billed platypus	VI	5β			X	Taurine	Mostly CA	HPLC	Also approximately 2% C_{27} bile acids. Very unusual to have C_{27} bile acids in any detectable amount in mammalian bile. C_{27} bile acids present are those dominant in Crocodylia (3α,7α,12α-trihydroxy)
Tachyglossus aculeatus	Short-nosed echidna	VI	5β			X	Taurine	Mostly CA	HPLC	Same observation as in platypus (see above).
**MARSUPALIA**										
Acrobates pygmaeus	Feather-tailed glider	VI	5β			X	Taurine	CDCA, CA, 1β-CDCA	HPLC	Unusual 1β-hydroxylation
Dactylopsila trivirgata	Australian striped opossum	VI	5β			X	Taurine	CA, CDCA, DCA	HPLC	
Dasyuroides byrnei	Kowari	VI	5β			X	Taurine	Mostly CA, minor amount of 7-oxoDCA	HPLC	
Dasyurus maculatus maculatus	Spotted-tailed quoll	VI	5β			X	Taurine	Mostly CA, minor amount of 7-oxoDCA	HPLC	
Dendrolagus doriana notatus	Doria's tree kangaroo	VI	5β			X	Taurine	CA, DCA, CDCA	HPLC	
Didelphis virginiana	North American opossum	VI	5β			X	Taurine	CA, CDCA, DCA	HPLC	
Lagostrophus fasciatus	Banded hare wallaby	VI	5β			X	Taurine	CA, DCA, CDCA	HPLC	Minor amount of 12-oxo bile acids
Macropus giganteus	Grey kangaroo	VI	5β			X	Taurine	CA, DCA, CDCA	HPLC	
Macropus parma	Parma wallaby	VI	5β			X	Taurine	CA, DCA, CDCA	HPLC	
Macropus rufus fruticus	Bennett's wallaby	VI	5β			X	Taurine	CA, DCA, CDCA	HPLC	Minor amount of 12-oxo bile acids
Macropus rufus rufus	Eastern red kangaroo	VI	5β			X	Taurine	CA, DCA, CDCA	HPLC	Minor amount of 12-oxo bile acids

Additional file 2, p. 3

Species		Bile salt class	5H	C_{27} alcohols	C_{27} acids	C_{24} acids	Conjugation	Major bile salts	Methodology	Comments
Petrogale xanthopus	Yellow-foot wallaby	VI	5β			X	Taurine	CA, DCA, CDCA	HPLC	
Phalanger maculatus	Spotted cuscus	VI	5β			X	Taurine	1α-OH CDCA, alloCDCA	HPLC	Major excreted bile acid is unconjugated 1α-OH CDCA. High amount of alloCDCA is very unusual for mammals (also seen in Anseriformes birds)
Phascolarctos adjustus	Queensland koala	VI	5β			X	Taurine	Almost entirely 7-oxoLCA	HPLC	
Sminthopsis crassicaudata	Fat-tailed dunnart	VI	5β			X	Taurine	Mostly CA	HPLC	
Thylogale brunii	Dusky pademelon	VI	5β			X	Taurine	CA, DCA, CDCA	HPLC	Minor amount of 12-oxo bile acids
Vombatus ursinus	Common wombat	VI	5β			X	Taurine	CDCA, 15α-OH bile acids	HPLC	Unusual 15α-OH bile acids.
Wallabia bicolor	Swamp wallaby	VI	5β			X	Taurine	CA, DCA, CDCA	HPLC	
**AFROTHERIA**										
**PROBOSCIDEA (order) Elephantidae (family)**										
Elephas maximus	Asian elephant	I	5β	X			Sulfate	Mixture of C_{27} bile alcohol sulfates (mostly 3α,7α,25,26-tetrahydroxy-5β-cholestan)	HPLC	Complete absence of bile acids in elephants and other Paenungulates; 25-hydroxylation of bile salts is unusual (also occurs in bile salts of lobe-fin fish)
Loxodonta africana	African elephant	I	5β	X			Sulfate	Mixture of C_{27} bile alcohol sulfates (mostly 3α,7α,25,26-tetrahydroxy-5β-cholestan)	HPLC, GC/MS, ESI/MS/MS	See comment for *Elephas maximus*
**HYRACOIDEA (order) Procaviidae (family)**										
Procavia capensis	Rock hyrax	I	5β	X			Sulfate	Mixture of C_{27} bile alcohol sulfates	HPLC	See comment for *Elephas maximus*
**SIRENIA (order)**										

Additional file 2, p. 4

Species		Bile salt class	5H	C_{27} alcohols	C_{27} acids	C_{24} acids	Conjugation	Major bile salts	Methodology	Comments
**Tricechidae (family)**										
Trichechus inunguis	Amazon manatee	I	5β	X			Sulfate	Mixture of trichechols and other C_{27} bile alcohol sulfates	HPLC	See comment for *Elephas maximus*
Trichechus manatus	West Indian manatee	I	5β	X			Sulfate	Mixture of trichechols and other C_{27} bile alcohol sulfates	HPLC, GC/MS	See comment for *Elephas maximus*
**TUBULIDENTATA (order) Orycteropodidae (family)**										
Orycteropus afer	South African aardvark	VI	5β			X	Taurine	CA, CDCA, DCA	HPLC	No C_{27} bile alcohols; complete switch to taurine conjugation
**MACROSCELIDEA (order) Macroscelididae (family)**										
Elephantulus rufescens	Rufous Sengi	VI	5β			X	Taurine	CA	HPLC	Very different bile salts than elephants, manatees, and hyraxes
**XENARTHRA**										
**CINGULATA (order) Dasypodidae (family)**										
Dasypus novemcinctus	Nine-banded armadillo	VI	5β			X	Taurine	CA, CDCA	HPLC	
**PILOSA (order)**										
**Myrmecophagidae (family)**										
Myrmecophaga tridactyla	Giant anteater	VI	5β			X	Taurine	CA, CDCA, DCA	HPLC	
Tamandua tetradactyla	Guyann tamandua	VI	5β			X	Taurine	CA, CDCA, DCA	HPLC	
**Bradypodidae (family)**										
Bradypus tridactylus	White-headed sloth	VI	5β			X	Glycine	CA, 7-oxoDCA	HPLC	Complete use of glycine conjugation
Bradypus variegatus	Brown-throated three-toed	VI	5β			X	Glycine	CA, 7-oxoDCA	HPLC	Complete use of glycine conjugation

Species		Bile salt class	5H	C_{27} alcohols	C_{27} acids	C_{24} acids	Conjugation	Major bile salts	Methodology	Comments
	sloth									
**LAURASIATHERIA**										
**EULIPOTYPHLA (order)**										
**Solenodontidae (family)**										
Solenodon paradoxus	Haitian solenodon	V	5β		X	X	Taurine	Mostly CA; some C_{27} bile acids	HPLC	Minor amounts of C_{27} bile alcohols
**Tenrecidae (family)**										
Echinops telfairi	Small Madagascar tenrec	VI	5β			X	Taurine	CA, CDCA	HPLC	
Hemicentetes semispinosus	Streaked tenrec	VI	5β			X	Taurine	CA, CDCA	HPLC	
**Erinaceidae (family)**										
Atelerix albiventris	Central African hedgehog	VI	5β			X	Taurine	CA, CDCA	HPLC, ESI/MS/MS	Traces of C_{27} bile alcohols also detected
Erinaceus europaeus	European hedgehog	VI	5β			X	Taurine	CA, CDCA	HPLC	
**CHIROPTERA (order)**										
**Desmodontidae (family)**										
Desmodus rotundus	Common vampire bat	VI	5β			X	Taurine	CA, CDCA	HPLC	
**Vespertilionidae (family)**										
Myotis lucifugus	Little brown myotis bat	VI	5β			X	Taurine	CA	HPLC	
**CARNIVORA (order)**										
**Ursidae (family)**										UDCA is found at >5% only in Ursidae and caviomorph rodents
Ailuropoda melanoleuca	Giant panda	VI	5β			X	Taurine	CA, CDCA, UDCA	HPLC	
Helarctos malayanus	Sun bear	VI	5β			X	Taurine	CDCA, CA, UDCA	HPLC	

Species		Bile salt class	5H	C_{27} alcohols	C_{27} acids	C_{24} acids	Conjugation	Major bile salts	Methodology	Comments
Melursus ursinus	Sloth bear	VI	5β			X	Taurine	CA, CDCA, UDCA	HPLC	
Selenarctos thibetanus	Asiatic black bear	VI	5β			X	Taurine	CDCA, UDCA	HPLC	
Thalarctos maritimus	Polar bear	VI	5β			X	Taurine	CDCA, UDCA, CA	HPLC	
Tremarctos ornatus	Spectacled bear	VI	5β			X	Taurine	CA, CDCA, UDCA	HPLC	
Ursus americanus	Black bear	VI	5β			X	Taurine	UDCA, CA, CDCA	HPLC	
Ursus arctos	Brown bear	VI	5β			X	Taurine	CA, CDCA, UDCA	HPLC	
**Canidae (family)**										
Alopex lagopus	Arctic fox	VI	5β			X	Taurine	CA, CDCA, DCA	HPLC	
Canis familiaris	Domestic dog	VI	5β			X	Taurine	CA, CDCA, DCA	HPLC	
Canis latrans	Coyote	VI	5β			X	Taurine	CA, CDCA, DCA	HPLC	
Canis lupus	Timber wolf	VI	5β			X	Taurine	CA, CDCA, DCA	HPLC	
Cerdocyon thous	Crab-eating fox	VI	5β			X	Taurine	CA, CDCA, DCA	HPLC	
Chrysocyon brachyurus	Maned wolf	VI	5β			X	Taurine	CA, CDCA, DCA	HPLC	
Dusicyon sechurae	Peruvian fox	VI	5β			X	Taurine	CA, CDCA, DCA	HPLC	
Fennecus zerda	Fennec fox	VI	5β			X	Taurine	CA, CDCA, DCA	HPLC	
Nyctereutes procyonoides	Raccoon dog	VI	5β			X	Taurine	CA, CDCA, DCA	HPLC	
Otocyon megalotis	Bat-eared fox	VI	5β			X	Taurine	CA, CDCA, DCA	HPLC	
Speothos venaticus	Guiana bush dog	VI	5β			X	Taurine	CA, CDCA	HPLC	
Urocyon cinereoargenteus	California grey fox	VI	5β			X	Taurine	CA, CDCA, DCA	HPLC	

Additional file 2, p. 7

Species		Bile salt class	5H	C_{27} alcohols	C_{27} acids	C_{24} acids	Conjugation	Major bile salts	Methodology	Comments
Vulpes corsac	Corsac fox	VI	5β			X	Taurine	CA, CDCA, DCA	HPLC	
**Viverridae (family)**										
Civettictis civetta	African civet	VI	5β			X	Taurine	Mostly CA; also CDCA	HPLC	
Genetta tigrina	Large spotted genet	VI	5β			X	Taurine	Mostly CA; also CDCA	HPLC	
Prionodon linsang	Banded linsang	VI	5β			X	Taurine	Mostly CA; also CDCA	HPLC	
Suricata suricatta	Meercat	VI	5β			X	Taurine	Mostly CA; also CDCA, DCA	HPLC	
**Hyaenidae (family)**										
Hyaena hyaena hyaena	Indian striped hyaena	VI	5β			X	Taurine	CA, DCA, CDCA	HPLC	
Parahyaena brunnea	Brown hyaena	VI	5β			X	Taurine	CA, DCA, CDCA	HPLC	
Protelese cristalus	Cape aardwolf	VI	5β			X	Taurine	CA, DCA, CDCA	HPLC	
**Herpestidae (family)**										
Helogale parvula	Dwarf mongoose	VI	5β			X	Taurine	Mostly CA; also CDCA, DCA	HPLC	
Mungos mungo	Banded mongoose	VI	5β			X	Taurine	Mostly CA; also CDCA	HPLC	
**Felidae (family)**										
Acinonyx jubatus	Cheetah	VI	5β			X	Taurine	Mostly CA, CDCA	HPLC	
Felis catus	Domestic cat	VI	5β			X	Taurine	Mostly CA, CDCA	HPLC	
Felis yagouaroundi	Panamanian jaguarondi	VI	5β			X	Taurine	Mostly CA, CDCA	HPLC	
Felis serval	Kenya serval	VI	5β			X	Taurine	Mostly CA, CDCA	HPLC	

Additional file 2, p. 8

Species		Bile salt class	5H	C_{27} alcohols	C_{27} acids	C_{24} acids	Conjugation	Major bile salts	Methodology	Comments
Felis silvestris tristami	Arabian wildcat	VI	5β			X	Taurine	Mostly CA, CDCA	HPLC	
Lynx rufus	Bobcat	VI	5β			X	Taurine	Mostly CA, CDCA	HPLC	
Neofelis nebulosa	Clouded leopard	VI	5β			X	Taurine	Mostly CA, CDCA	HPLC	
Panthera leo	African lion	VI	5β			X	Taurine	Mostly CA, CDCA	HPLC	
Panthera onca onca	Brazilian jaguar	VI	5β			X	Taurine	Mostly CA, CDCA	HPLC	
Panthera leo persica	Asian lion	VI	5β			X	Taurine	Mostly CA, CDCA	HPLC	
Panthera pardus japonensis	North Chinese leopard	VI	5β			X	Taurine	Mostly CA, CDCA	HPLC	
Panthera pardus saxicolor	Persian leopard	VI	5β			X	Taurine	Mostly CA, CDCA	HPLC	
Panthera tigris altaica	Siberian tiger	VI	5β			X	Taurine	Mostly CA, CDCA	HPLC	
Panthera tigris corbetti	Corbett's tiger	VI	5β			X	Taurine	Mostly CA, CDCA	HPLC	
Panthera tigris	Sumatran tiger	VI	5β			X	Taurine	Mostly CA, CDCA	HPLC	
Panthera uncia	Snow leopard	VI	5β			X	Taurine	Mostly CA, CDCA	HPLC	
Prionailurus bengalensis euptilura	Amur leopard cat	VI	5β			X	Taurine	Mostly CA, CDCA	HPLC	
Prionailurus viverrinus	Fishing cat	VI	5β			X	Taurine	Mostly CA, CDCA	HPLC	
Puma concolor	Mountain lion	VI	5β			X	Taurine	Mostly CA, CDCA	HPLC	
**Phocidae (family)**										Unusual 23*R*-hydroxylation found in Phocidae, Otariidae, and Odobenidae but not Mustelidae
Phoca hispida	Ringed seal	VI	5β			X	Taurine	23*R*-OH-CDCA, CA, DCA	HPLC	

Species		Bile salt class	5H	C_{27} alcohols	C_{27} acids	C_{24} acids	Conjugation	Major bile salts	Methodology	Comments
Phoca sibirica	Baikal seal	VI	5β			X	Taurine	23*R*-OH-CDCA, CA, DCA	HPLC	
Phoca vitulina	Harbor seal	VI	5β			X	Taurine	23*R*-OH-CDCA, CA, DCA	HPLC	
**Otariidae (family)**										Unusual 23*R*-hydroxylation found in Phocidae, Otariidae, and Odobenidae but not Mustelidae
Arctophalus pusillus	Australian fur seal	VI	5β			X	Taurine	23*R*-OH-CDCA, CA, DCA	HPLC	
Callorhinus ursinus	Northern fur seal	VI	5β			X	Taurine	23*R*-OH-CDCA, CA, DCA	HPLC	
Eumetopias jubatus	Northern sea lion	VI	5β			X	Taurine	CA, 23*R*-OH-CDCA, DCA	HPLC	
Mirounga angustrirostris	Northern elephant seal	VI	5β			X	Taurine	23*R*-OH-CDCA, 23-*R*-OH-DCA, 23*R*-CA	HPLC	
Otaria flavescens	Southern sea lion	VI	5β			X	Taurine	23*R*-OH-CDCA, CA, DCA	HPLC	
Zalophus californianus	California sea lion	VI	5β			X	Taurine	CA, 23*R*-OH-CDCA, DCA	HPLC	
**Odobenidae (family)**										Unusual 23*R*-hydroxylation found in Phocidae, Otariidae, and Odobenidae but not Mustelidae
Obodenus rosmarus	Atlantic walrus	VI	5β			X	Taurine	23*R*-OH-CDCA, CA, DCA	HPLC	
Obodenus rosmarus divergens	Pacific walrus	VI	5β			X	Taurine	23*R*-OH-CDCA, CA, DCA	HPLC	
**Procyonidae (family)**										
Bassariscus astulus arizonensis	Arizona cacomistle	VI	5β			X	Taurine	CA, CDCA	HPLC	
Nasua narica yucatanica	Yucatan white-nosed coatimundi	VI	5β			X	Taurine	CA, CDCA	HPLC	
Nasua nasua	Ring-tailed	VI	5β			X	Taurine	CA, CDCA	HPLC	

Species		Bile salt class	5H	C_{27} alcohols	C_{27} acids	C_{24} acids	Conjugation	Major bile salts	Methodology	Comments
	coati									
Potos flavus	Kinkajou	VI	5β			X	Taurine	CDCA, CA	HPLC	Similar to giant panda bear but has high amount of CDCA and absence of secondary bile acids
Procyon lotor	Raccoon	VI	5β			X	Taurine	CA, CDCA	HPLC	
**Mephitidae (family)**										
Mephitis mephitis	Striped skunk	VI	5β			X	Taurine	CA, CDCA	HPLC	
**Ailuridae (family)**										
Ailurus fulgens	Red panda	VI	5β			X	Taurine	CA, CDCA	HPLC	
**Mustelidae (family)**										
Amblonyx cinerea	Small-claw otter	VI	5β			X	Taurine	CA, CDCA, DCA	HPLC	
Eira barbata	Grey-headed tayra	VI	5β			X	Taurine	Mostly CA; some CDCA	HPLC	
Enhydra lutra	Sea otter	VI	5β			X	Taurine	CA, CDCA	HPLC	
Lutra canadensis	Canadian otter	VI	5β			X	Taurine	Mostly CA	HPLC	
Lutra lutra	Eurasian otter	VI	5β			X	Taurine	CA, CDCA	HPLC	
Lutra maculicollis	Spotted-neck otter	VI	5β			X	Taurine	CA, CDCA, DCA	HPLC	
Lutra platensis	South American otter	VI	5β			X	Taurine	CA, CDCA	HPLC	
Lutra sumatrana	Hairy-nosed otter	VI	5β			X	Taurine	CA, CDCA	HPLC	
Martes flavigula	Siberian yellow-throated marten	VI	5β			X	Taurine	CA, CDCA	HPLC	
Martes pennanti	Fisher	VI	5β			X	Taurine	Mostly CA	HPLC	
Mellivora capensis vernayi	Vernay's ratel	VI	5β			X	Taurine	Mostly CA; some CDCA	HPLC	
Mustela lutreola	Northern	VI	5β			X	Taurine	CA	HPLC	

Species		Bile salt class	5H	C_{27} alcohols	C_{27} acids	C_{24} acids	Conjugation	Major bile salts	Methodology	Comments
	European mink									
Mustela putorius	Steppe polecat	VI	5β			X	Taurine	Mostly CA	HPLC	
Mustela putorius furo	European ferret	VI	5β			X	Taurine	Mostly CA	HPLC	
Mustela sibirica	Siberian weasel	VI	5β			X	Taurine	Mostly CA	HPLC	
Neovison vison	American mink	VI	5β			X	Taurine	Mostly CA	HPLC	
**PERISSODACTYLA (order)**										
**Rhinocerotidae (family)**										
Ceratotherium simum cottoni	Northern white rhinoceros	I	5β	X		X	Sulfate, taurine	Mostly C_{27} bile alcohol sulfates	HPLC, GC/MS	
Ceratotherium simum simum	Southern white rhinoceros	I	5β	X		X	Sulfate, taurine	Mostly C_{27} bile alcohol sulfates	HPLC, GC/MS	
Dicerorhinus sumatrensis	Sumatran rhinoceros	I	5β	X		X	Sulfate, taurine	Mostly 3α,7α,12α,27-tetrahydroxy-5β-cholestan	HPLC, GC/MS	
Diceros bicornis	Black rhinoceros	I	5β	X		X	Sulfate, taurine	Mostly 3α,7α,12α,27-tetrahydroxy-5β-cholestan	HPLC, GC/MS	
Rhinoceros unicornis	Indian rhinoceros	I	5β	X		X	Sulfate, taurine	Mostly C_{27} bile alcohol sulfates	HPLC, GC/MS	
**Tapiridae (family)**										
Tapirus bairdii	Bairds tapir	VI	5β			X	Glycine	CDCA, CA, DCA	HPLC	
**ARTIODACTYLA (order)**										
**Equidae (family)**										
Equus burchelli antiquarum	Damara zebra	VI	5β		X	X	Glycine, taurine	C_{27} bile acids, CA	HPLC	
**Suidae (family)**										6α-hydroxylation unusual in mammals (also found in

Species		Bile salt class	5H	C_{27} alcohols	C_{27} acids	C_{24} acids	Conjugation	Major bile salts	Methodology	Comments
										chevrotains and mice). Only mammal other than members of Suidae to have HCA as a primary bile acid is the lesser Malay chevrotain.
Sus scrofa domestica	Domestic pig	VI	5β			X	Glycine, taurine	HCA, HDCA, CDCA, CA	HPLC	
Sus scrofa scrofa	Central European wild boar	VI	5β			X	Glycine, taurine	HCA, HDCA, CDCA, CA	HPLC	
Phacochoerus africanus sundevalli	Southern warthog	VI	5β			X	Glycine, taurine	HCA, HDCA, CDCA, CA	HPLC	
Phacochoerus arthiopicus	East African warthog	VI	5β			X	Glycine, taurine	HCA, HDCA, CDCA, CA	HPLC	
Potanochoerus porcus	Red River hog	VI	5β			X	Glycine, taurine	HCA, HDCA, CDCA, CA	HPLC	
**Hippopotamidae (family)**										
Choeropsis liberiensis	Pygmy hippopotamus	VI	5β			X	Glycine, taurine	CDCA, CA, DCA	HPLC	High proportion of glycine conjugation.
Hippopotamus amphibius	River hippopotamus	VI	5β			X	Glycine, taurine	CDCA, CA, DCA	HPLC	Bile acid profile very similar to that of humans
**Physeteridae (family)**										Very high proportion of DCA in biliary bile.
Kogia breviceps	Pygmy sperm whale	VI	5β			X	Taurine	DCA, CDCA, CA	HPLC, GC/MS	LCA and UDCA also found
Kogia simus	Dwarf sperm whale	VI	5β			X	Taurine	DCA, CDCA, CA	HPLC, GC/MS	LCA and UDCA also found
Physeter catodon	Sperm whale	VI	5β			X	Taurine	DCA, CDCA, CA	HPLC, GC/MS, ESI/MS/MS	LCA and UDCA also found; trace levels (< 5%) of glycine conjugation (evolutionary remnant not found in other whales?)
**Delphinidae (family)**										
Cephalorhynchus commersoni	Commerson's dolphin	VI	5β			X	Taurine	Mostly CA; small amounts of CDCA	HPLC	

Species		Bile salt class	5H	C_{27} alcohols	C_{27} acids	C_{24} acids	Conjugation	Major bile salts	Methodology	Comments
Delphinus delphis	Saddleback dolphin	VI	5β			X	Taurine	Mostly CA; small amounts of CDCA	HPLC	
Feresa attenuata	Pygmy killer whale	VI	5β			X	Taurine	Mostly CA; small amounts of CDCA	HPLC	
Lagenorhynchus albirostris	White dolphin	VI	5β			X	Taurine	Mostly CA; small amounts of CDCA	HPLC	
Lissodelphis borealis	Right whale dolphin	VI	5β			X	Taurine	Mostly CA; small amounts of CDCA	HPLC	
Orcinus orca	Killer whale	VI	5β			X	Taurine	Mostly CA; small amounts of CDCA	HPLC	
Pseudorca crassidens	False killer whale	VI	5β			X	Taurine	Mostly CA; small amounts of CDCA	HPLC	
Stenella attenuata	Spotted dolphin	VI	5β			X	Taurine	Mostly CA; small amounts of CDCA	HPLC	
Steno bredanensis	Rough-tooth dolphin	VI	5β			X	Taurine	Mostly CA; small amounts of CDCA	HPLC	
Tursiops truncatus	Bottlenose dolphin	VI	5β			X	Taurine	Mostly CA; small amounts of CDCA	HPLC	
**Tragulidae (family)**										6α-hydroxylation uncommon in mammals (also found in Suidae and mice)
Tragulus javanicus	Lesser Malay chevrotain	VI	5β			X	Glycine, taurine	CA, HCA, CDCA	HPLC	
**Giraffidae (family)**										All have 100% or nearly 100% glycine conjugation
Giraffa camelopardalis	Baringo giraffe	VI	5β			X	Glycine	CA, DCA	HPLC	
Giraffa rothschildi	Uganda giraffe	VI	5β			X	Glycine	CA, DCA	HPLC	
Giraffa tippelskirchi	Masai giraffe	VI	5β			X	Glycine	CA, DCA	HPLC	
Okapia johnstoni	Okapi	VI	5β			X	Glycine	CA, DCA	HPLC	
**Moschidae (family)**										

Species		Bile salt class	5H	C_{27} alcohols	C_{27} acids	C_{24} acids	Conjugation	Major bile salts	Methodology	Comments
Moschus moschiferus moschiferus	Siberian musk deer	VI	5β			X	Glycine, taurine	CA, CDCA	HPLC, ESI/MS/MS	
**Cervidae (family)**										Significant presence of C_{27} bile alcohols consistent with ungulate ancestry.
Cervus elaphus scoticus	British red deer	III	5β	X		X	Glycine, taurine, sulfate	CA, CDCA; C_{27} bile alcohol sulfates	HPLC, ESI/MS/MS	
Cervus elaphus sibiricus	Altai wapiti	III	5β	X		X	Glycine, taurine, sulfate	CA, CDCA; C_{27} bile alcohol sulfates	HPLC, ESI/MS/MS	
Mazama americana	Mexican red brocket deer	III	5β	X		X	Glycine, taurine, sulfate	CA, CDCA; C_{27} bile alcohol sulfates	HPLC, ESI/MS/MS	
**Bovidae (family)**										
Addax nasomaculatus	Addax	VI	5β			X	Taurine, glycine	CDCA, CA, DCA	HPLC	
Aepyceros melampus melampus	South African impala	VI	5β			X	Taurine	CA, DCA, CDCA	HPLC	
Aepyceros melampus petersi	Black-faced impala	VI	5β			X	Taurine	CA, DCA, CDCA	HPLC	
Aepyceros melampus rendilis	Kenya impala	VI	5β			X	Taurine	CA, DCA, CDCA	HPLC	
Alcelaphus buselaphus caama	Cape hartebeest	VI	5β			X	Glycine, taurine	CA, CDCA, DCA	HPLC	
Alcelaphus buselaphus jacksoni	Jackson's hartebeest	VI	5β			X	Glycine, taurine	CA, CDCA, DCA	HPLC	
Antidorcas marsupialis angolensis	Angolan springbok	VI	5β			X	Taurine	CA, CDCA, DCA	HPLC	
Antilope	Blackbuck	VI	5β			X	Taurine,	CA, CDCA, DCA	HPLC	Only member of Antilopini with

Species		Bile salt class	5H	C_{27} alcohols	C_{27} acids	C_{24} acids	Conjugation	Major bile salts	Methodology	Comments
cervicapra							glycine			glycine conjugation
Bison athabascae	Wood bison	VI	5β			X	Glycine, taurine	CA, CDCA, DCA	HPLC	
Bison bonasus	Lowland wisent	VI	5β			X	Taurine, glycine	CA, CDCA, DCA	HPLC	
Bos gaurus	Indian gaur	VI	5β			X	Glycine, taurine	CA, CDCA, DCA	HPLC	
Bos javanicus javanicus	Javan banteng	VI	5β			X	Glycine, taurine	CA, CDCA, DCA	HPLC	
Bos taurus	Domestic cow	VI	5β			X	Glycine, taurine	CA, CDCA, DCA	HPLC	
Boselaphis tragocamelus	Indian nilgai	VI	5β			X	Glycine, taurine	CA, CDCA, DCA	HPLC	Nearly total glycine conjugation; most primitive member of family?
Bubalus bubalus	Water buffalo	VI	5β			X	Glycine, taurine	CA, CDCA, DCA	HPLC	
Bubalus depressicornis	Lowland anoa	VI	5β			X	Glycine, taurine	CA, CDCA, DCA	HPLC	
Budorcas taxicolor taxicolor	Mishimi takin	VI	5β			X	Glycine, taurine	CA, DCA, CDCA	HPLC	
Capra cylindricornis	Eastern Caucasian tur	VI	5β			X	Taurine	CA, CDCA, DCA	HPLC	
Capra domesticus	Domestic saanen goat	VI	5β			X	Taurine	CA, CDCA, DCA	HPLC	
Capra falconeri	Turkomen markhor	VI	5β			X	Taurine	CA, CDCA, DCA	HPLC	
Capra ibex ibex	Alpine ibex	VI	5β			X	Taurine	CA, CDCA, DCA	HPLC	
Capra ibex sibirica	Siberian ibex	VI	5β			X	Taurine	CA, CDCA, DCA	HPLC	
Capra nubiana	Nubian ibex	VI	5β			X	Taurine	CA, CDCA, DCA	HPLC	
Cephalophus sylvicultor	Yellow-backed duiker	VI	5β			X	Glycine	CA, CDCA, DCA	HPLC	Total conjugation with glycine
Connochaetes albojubatus	White-bearded gnu	VI	5β			X	Glycine, taurine	CA, CDCA, DCA	HPLC	
Connachaetes gnoli	White-tailed gnu	VI	5β			X	Glycine, taurine	CA, CDCA, DCA	HPLC	

Species		Bile salt class	5H	C_{27} alcohols	C_{27} acids	C_{24} acids	Conjugation	Major bile salts	Methodology	Comments
Connochartes taurinus	Brindled gnu	VI	5β			X	Glycine, taurine	CA, CDCA, DCA	HPLC	
Damaliscus hunteri	Hunter's hartebeest	VI	5β			X	Glycine, taurine	CA, CDCA, DCA	HPLC	
Damaliscus lunatus jimela	Jimela topi	VI	5β			X	Glycine, taurine	CA, CDCA, DCA	HPLC	
Damaliscus phillipi	Blesbok	VI	5β			X	Glycine, taurine	CA, CDCA, DCA	HPLC	
Damaliscus pygargus pygargus	Bontebok	VI	5β			X	Glycine, taurine	CA, CDCA, DCA	HPLC	
Gazella cuvieri	Cuvier's gazelle	VI	5β			X	Taurine	CA, CDCA, DCA	HPLC	
Gazella dama	Dama gazelle	VI	5β			X	Taurine	CA, CDCA, DCA	HPLC	
Gazella dorcas	Dorcas gazelle	VI	5β			X	Taurine	CA, CDCA, DCA	HPLC	
Gazella leptoceros	Slender-horn gazelle	VI	5β			X	Taurine	CA, CDCA, DCA	HPLC	
Gazella rufifrons laevipes	Sudan red-fronted gazelle	VI	5β			X	Taurine	CA, CDCA, DCA	HPLC	
Gazella soemmerringi	Soemerring's gazelle	VI	5β			X	Taurine	CA, CDCA, DCA	HPLC	
Gazella subgutturosa	Persian goitered gazelle	VI	5β			X	Taurine	CA, CDCA, DCA	HPLC	
Hemitragus hylocrius	Nilgiri tahr	VI	5β			X	Taurine	CA, CDCA, DCA	HPLC	
Hemitragus jernlahicus	Himalayan tahr	VI	5β			X	Taurine	CA, CDCA, DCA	HPLC	
Hippotraeus niger niger	South African sable antelope	VI	5β			X	Taurine, glycine	CDCA, CA, DCA	HPLC	
Kobus defassa	Defassa waterbuck	VI	5β			X	Glycine, taurine	CA, CDCA, DCA	HPLC	
Kobus ellipsiprymnus	Ellipsen waterbuck	VI	5β			X	Glycine, taurine	CA, CDCA, DCA	HPLC	

Species		Bile salt class	5H	C_{27} alcohols	C_{27} acids	C_{24} acids	Conjugation	Major bile salts	Methodology	Comments
Kobus leche leche	Red lechwe	VI	5β			X	Glycine, taurine	CA, CDCA, DCA	HPLC	
Kobus leche robertsi	Kafue lechwe	VI	5β			X	Glycine, taurine	CA, CDCA, DCA	HPLC	
Kobus megaceros	Nile lechwe	VI	5β			X	Glycine, taurine	CA, CDCA, DCA	HPLC	
Litocranius walleri walleri	Southern gerenuk	VI	5β			X	Taurine	CA, CDCA, DCA	HPLC	
Madoqua guentheri	Guenther's dik dik	VI	5β			X	Taurine, glycine	CA, CDCA, DCA	HPLC, ESI/MS/MS	
Madoqua kirkii cavendishi	Cavendish's dik dik	VI	5β			X	Taurine, glycine	CA, CDCA, DCA	HPLC	
Madoqua kirkii kirkii	Kirk's dik dik	VI	5β			X	Taurine, glycine	CA, CDCA, DCA	HPLC	
Nemorhaedus goral	Goral	VI	5β			X	Taurine, glycine	CA, CDCA, DCA	HPLC	
Nesotragus moschatus	Zulu suni	VI	5β			X	Taurine, glycine	CA, CDCA, DCA	HPLC	
Nesotragus moschatus akeleyi	Kenya suni	VI	5β			X	Taurine, glycine	CA, CDCA, DCA	HPLC	
Oreamnos americanus	Rocky Mountain goat	VI	5β			X	Taurine, glycine	CA, CDCA, DCA	HPLC	
Oreotragus oreotragus schillingsi	Masai klipspringer	VI	5β			X	Glycine, taurine	CA, CDCA, DCA	HPLC	
Oryx dammah	Scimitar-horn oryx	VI	5β			X	Taurine, glycine	CDCA, CA, DCA	HPLC	
Oryx gazella beisa	Beisa oryx	VI	5β			X	Taurine, glycine	CDCA, CA, DCA	HPLC	
Oryx leucoryx	Arabian oryx	VI	5β			X	Taurine, glycine	CDCA, CA, DCA	HPLC	
Ourebia ourebi cottoni	Cotton's oribi	VI	5β			X	Taurine, glycine	CA, CDCA, DCA	HPLC	
Ovibos crispus	Japanese serow	VI	5β			X	Taurine, glycine	CA, DCA, CDCA	HPLC	
Ovibos	Musk ox	VI	5β			X	Taurine,	CA, CDCA, DCA	HPLC	

Species		Bile salt class	5H	C_{27} alcohols	C_{27} acids	C_{24} acids	Conjugation	Major bile salts	Methodology	Comments
moschatus							glycine			
Ovis aries	Domestic sheep	VI	5β			X	Taurine	CA, CDCA, DCA	HPLC	
Ovis canadensis nelsoni	Peninsula desert bighorn sheep	VI	5β			X	Taurine	CA, CDCA, DCA	HPLC	
Ovis cycloceros	Afghan urial	VI	5β			X	Taurine	CA, CDCA, DCA	HPLC	
Ovis musimon	European mouflon	VI	5β			X	Taurine	CA, CDCA, DCA	HPLC	
Ovis nivicola nivicola	Kamchatan snow sheep	VI	5β			X	Taurine, glycine	CA, DCA, CDCA	HPLC	
Ovis orientalis	Armenian mouflon	VI	5β			X	Taurine	CA, CDCA, DCA	HPLC	
Ovis vignei arkal	Transcaspian urial	VI	5β			X	Taurine	CA, CDCA, DCA	HPLC	
Pseudois nayaur szechuanensis	Chinese bharal	VI	5β			X	Taurine	CA, CDCA, DCA	HPLC	
Rubicapra rubicapra	Chamois	VI	5β			X	Taurine, glycine	CA, CDCA, DCA	HPLC	
Saiga tartarica	Russian saiga	VI	5β			X	Taurine	CA, CDCA, DCA	HPLC	
Syncerus caffer	Cape buffalo	VI	5β			X	Glycine, taurine	CA, DCA, CDCA	HPLC	
Syncerus nanus	Forest buffalo	VI	5β			X	Glycine, taurine	CA, CDCA, DCA	HPLC	
Taurotragus derbianus gigas	East giant eland	VI	5β			X	Glycine, taurine	CA, CDCA, DCA	HPLC	
Taurotragus pattersonianus	Patterson's eland	VI	5β			X	Glycine, taurine	CA, CDCA, DCA	HPLC	
Taurotragus spekki	Speke's sitatunga	VI	5β			X	Glycine, taurine	CA, CDCA, DCA	HPLC	
Tragelaphus angasi	Lowland nyala	VI	5β			X	Taurine, glycine	CA, CDCA, DCA	HPLC	
Tragelaphus euryceros	East African bongo	VI	5β			X	Taurine, glycine	CA, CDCA, DCA	HPLC	
Tragelaphus imberbis	Lesser kudu	VI	5β			X	Taurine	CA, DCA, CDCA	HPLC	Complete transition to taurine conjugation

Additional file 2, p. 19

Species		Bile salt class	5H	C_{27} alcohols	C_{27} acids	C_{24} acids	Conjugation	Major bile salts	Methodology	Comments
Tragelaphus strepsiceros	Greater kudu	VI	5β			X	Taurine, glycine	CA, CDCA, DCA	HPLC	
**SCANDENTIA (order) Tupaiide (family)**										
Tupaia glis	Tree shrew	VI	5β			X	Taurine	CA, CDCA	HPLC	
Tupaia minor	Lesser tree shrew	VI	5β			X	Taurine	CA, CDCA	HPLC	
Tupaia tana	Long-nosed tree shrew	VI	5β / 5α			X	Taurine	CA, CDCA, alloCA	HPLC	
**PRIMATES (order)**										
**Lemuridae (family)**										Unusual to have nearly 100% CDCA
Eulemur coronatus	Crowned lemur	VI	5β			X	Taurine	Mostly CDCA	HPLC	
Eulemur fulvus collaris	Collared lemur	VI	5β			X	Taurine	Mostly CDCA	HPLC	
Eulemur fulvus fulvus	Brown lemur	VI	5β			X	Taurine	Mostly CDCA	HPLC	
Eulemur macaco macaco	Black lemur	VI	5β			X	Taurine	Mostly CDCA	HPLC	
Eulemur mongoz	Mongoose lemur	VI	5β			X	Taurine	Mostly CDCA	HPLC	
Hapalemur griseus griseus	Gentle gray lemur	VI	5β			X	Taurine	Mostly CDCA	HPLC	
Lemur catta	Ring-tail lemur	VI	5β			X	Taurine	Mostly CDCA	HPLC	
Varecia varecia rubra	Black and White ruffed lemur	VI	5β			X	Taurine	Mostly CDCA	HPLC	
Varecia varecia variegatus	Red-ruffed lemur	VI	5β			X	Taurine	Mostly CDCA	HPLC	
**Cheirogaleidae (family)**										
Cheirogaleus medius	Fat-tailed dwarf lemur	V	5β		X	X	Taurine	Mixture of C_{24} and C_{27} bile acids	HPLC	
Mirza coquereli	Greater	V	5β		X	X	Taurine	Mixture of C_{24} and C_{27}	HPLC, ESI/MS/MS	

Species		Bile salt class	5H	C_{27} alcohols	C_{27} acids	C_{24} acids	Conjugation	Major bile salts	Methodology	Comments
	mouse lemur							bile acids		
**Indridae (family)**										
Propithecus verreauxi coquereli	Coquerel's sifaka	VI	5β			X	Glycine, taurine	Unusual mix of 7-oxo and 7β-OH bile acids. Essentially no CA.	HPLC	
**Galagonidae (family)**										
Galago senegalensis	Lesser bushbaby	V	5β		X	X	Taurine	Mixture of C_{24} and C_{27} bile acids; mostly CA	HPLC, ESI/MS/MS	
Loris tardigradus	Slender loris	V	5β		X	X	Taurine	Mixture of C_{24} and C_{27} bile acids	HPLC	
Otolemur garnettii	Small-eared bushbaby	V	5β		X	X	Taurine	Mixture of C_{24} and C_{27} bile acids	HPLC, ESI/MS/MS	
Nycticebus coucang bengalensis	Bengal slow loris	V	5β		X	X	Taurine	Mixture of C_{24} and C_{27} bile acids	HPLC	
Nycticebus coucang coucang	Malayan slow loris	V	5β		X	X	Taurine	Mixture of C_{24} and C_{27} bile acids	HPLC	
Nycticebus pygmaeus	Pygmy loris	V	5β		X	X	Taurine	Mixture of C_{24} and C_{27} bile acids	HPLC	
**Tarsiidae (family)**										
Tarsius syrichta	Philippine tarsier	VI	5β			X	Taurine	CA, CDCA	ESI/MS/MS	
**Hominidae (family)**										
Gorilla gorilla gorilla	West lowland gorilla	VI	5β			X	Glycine, taurine	CA, CDCA	HPLC	
Homo sapiens	Humans	VI	5β			X	Glycine, taurine	CA, CDCA	HPLC	
Pan paniscus	Bonobo	VI	5β			X	Glycine, taurine	CA, CDCA	HPLC	
Pan troglodytes	Chimpanzee	VI	5β			X	Glycine, taurine	CA, CDCA	HPLC	
Pongo pongo	Sumatran	VI	5β			X	Glycine,	CA, CDCA	HPLC	

Species		Bile salt class	5H	C_{27} alcohols	C_{27} acids	C_{24} acids	Conjugation	Major bile salts	Methodology	Comments
abelii	orangutan						taurine			
Pongo pongo pymaeus	Bornean orangutan	VI	5β			X	Glycine, taurine	CA, CDCA	HPLC	
**Cebidae (family)**										
Aotus azarai	Red-neck douroucouli	VI	5β			X	Taurine	CA, CDCA	HPLC	
Aotus trivirgatus	Night monkey	VI	5β			X	Taurine	CA, CDCA	HPLC	
Callimico goeldii	Goeldi's marmoset	VI	5β			X	Taurine	CA, CDCA	HPLC	
Callithrix geoffroyi	Geoffroy's marmoset	VI	5β			X	Taurine	CA, CDCA	HPLC	
Cebuella pygmaea	Pygmy marmoset	VI	5β			X	Taurine	CA, CDCA	HPLC	
Leontopithecus rosalia rosalia	Golden-lion tamarin	V	5β		X	X	Taurine (94%), glycine (about 6%)	CA (50%), CDCA (37%), C_{27} bile acids (~10%)	HPLC	Unusual to detect C_{27} bile acids in mammals. C_{27} bile acids may reflect ancestral bile salt background
Saguinus oedipus	Cotton-top tamarin	VI	5β			X	Taurine	CA, CDCA	HPLC	
Saimiri sciureus	Squirrel monkey	VI	5β			X	Taurine	CA, CDCA	HPLC	
**Cercopithecidae (family)**										Glycine conjugation indicates recent transition away from the bile alcohol sulfates
Allenopithecus nigroviridis	Allen's swamp guenon	VI	5β			X	Glycine, taurine	CA, CDCA	HPLC	
Cercocebus galeritus	Agile mangabey	VI	5β			X	Glycine, taurine	CA, CDCA, DCA	HPLC	
Cercocebus torquatus	White mangabey	VI	5β			X	Glycine, taurine	CA, CDCA, DCA	HPLC	
Cercopithecus ascanius schmidti	Schmidt's spot-nosed guenon	VI	5β			X	Glycine, taurine	CA, CDCA	HPLC	
Cercopithecus	Campbell's	VI	5β			X	Glycine,	CA, CDCA, DCA	HPLC	

Species		Bile salt class	5H	C_{27} alcohols	C_{27} acids	C_{24} acids	Conjugation	Major bile salts	Methodology	Comments
campbelli	guenon						taurine			
Cercopithecus cephus	Moustached guenon	VI	5β			X	Glycine, taurine	CA, CDCA	HPLC	
Cercopithecus diana	Diana's guenon	VI	5β			X	Glycine, taurine	CA, CDCA, DCA	HPLC	
Cercopithecus hamlyni	Hamlyn's guenon	VI	5β			X	Glycine, taurine	CA, CDCA, DCA	HPLC	
Cercopithecus neglectus	Debrazza's guenon	VI	5β			X	Glycine, taurine	CA, CDCA	HPLC	
Cercopithecus schlateri	Schlater's guenon	VI	5β			X	Glycine, taurine	CA, CDCA, DCA	HPLC	
Cercopithecus schmidti	Red-tail guenon	VI	5β			X	Glycine, taurine	CA, CDCA, DCA	HPLC	
Colobus angolensis	Angolan colobus	VI	5β			X	Glycine, taurine	CA, CDCA	HPLC	
Colobus guereza kikuyuensis	Kikuyu colobus	VI	5β			X	Glycine, taurine	CA, CDCA	HPLC	
Macaca mulatta	Rhesus macaque	VI	5β			X	Glycine, taurine	CA, CDCA, DCA	HPLC	
Macaca nemestrina	Pig-tailed macaque	VI	5β			X	Glycine, taurine	CA, CDCA	HPLC	
Macaca nigra	Sulawesi crested macaque	VI	5β			X	Glycine, taurine	CA, CDCA	HPLC	
Macaca radiata	Bonnet macaque	VI	5β			X	Glycine, taurine	CA, CDCA, DCA	HPLC	
Macaca silenus	Lion-tailed macaque	VI	5β			X	Glycine, taurine	CA, CDCA, DCA	HPLC	
Papio cynocephalus anubis	Savannah baboon	VI	5β			X	Glycine, taurine	CA, CDCA	HPLC	
Papio leucophaeus	Mainland drill	VI	5β			X	Glycine, taurine	CA, CDCA, DCA	HPLC	
Pygathrix nemaeus	Douc langur	VI	5β			X	Glycine, taurine	CA, CDCA	HPLC	
Pygathrix roxellana	Golden snub-nosed monkey	VI	5β			X	Glycine, taurine	CA, CDCA	HPLC	

Species		Bile salt class	5H	C_{27} alcohols	C_{27} acids	C_{24} acids	Conjugation	Major bile salts	Methodology	Comments
Semnopithecus entellus	Hanuman langur	VI	5β			X	Glycine, taurine	CA, CDCA	HPLC	
Semnopithecus francoisi	Francois langur	VI	5β			X	Glycine, taurine	CA, CDCA	HPLC	
**Pitheciidae (family)**										
Callicebus donacophilus donacophilus	Bolivian grey titi	VI	5β			X	Taurine	CDCA, CA	HPLC	
Callicebus moloch	Dusky titi	VI	5β			X	Taurine	CDCA, CA	HPLC	
Pithecia pithecia)	White-faced saki	VI	5β			X	Taurine	CDCA, CA	HPLC	
**Atelidae (family)**										
Alouatta palliata	Mantled howler	VI	5β			X	Glycine, taurine	CA, CDCA, DCA	HPLC	
Alouatta seniculus	Red howler	VI	5β			X	Glycine, taurine	CA, CDCA, DCA	HPLC	
Alouatta villosa	Mexican black howler monkey	VI	5β			X	Glycine, taurine	CA, CDCA, DCA	HPLC	
Ateles beizebuth	Colombian brown spider monkey	VI	5β			X	Glycine, taurine	CDCA, DCA	HPLC	
Ateles geoffroyi	Black-handed spider monkey	VI	5β			X	Glycine, taurine	CA, CDCA, DCA	HPLC	
Lagothrix lagothricha	Humboldt's woolly monkey	VI	5β			X	Glycine, taurine	CA, CDCA, DCA	HPLC	
**LAGOMORPHA (order)**										
**Leporidae (family)**										
Oryctolagus cuniculus	Domestic rabbit	VI	5β			X	Glycine, taurine	DCA	HPLC, ESI/MS/MS	Very unusual in having DCA as major bile acid
Sylvilagus	Brush rabbit	V	5β			X	Glycine,	DCA		

Species		Bile salt class	5H	C_{27} alcohols	C_{27} acids	C_{24} acids	Conjugation	Major bile salts	Methodology	Comments
bachmani							taurine			
**RODENTIA (order)**										
**Sciuridae (family)**										
Callosciurus prevosti	Bornean prevost squirrel	VI	5β			X	Taurine	CDCA, CA, other C_{24} bile acids	HPLC	
Sciurus niger	Fox squirrel	VI	5α / 5β			X	Taurine	alloCA, other C_{24} bile acids	HPLC	High amount of alloCA for a mammal
Spermophilus tridecemlineatus	Thirteen-lined ground squirrel	VI	5β			X	Taurine	CA, CDCA	HPLC	
**Capromyidae (family)**										
Geocapromys browni	Jamaican hutia	III	5β	X		X	Glycine, sulfate, taurine	CA, C_{27} bile alcohol sulfates	HPLC	Unusual bile salt profile
Geocapromys ingrahami	Bahamian hutia	III	5β	X		X	Glycine, sulfate, taurine	CA, C_{27} bile alcohol sulfates	HPLC	Unusual C_{24} bile acids with oxo groups
**Cuniculae (family)**										
Cuniculus taczonowskii	Mountain paca	VI	5β			X	Glycine	Δ22-7-OxoLCA	HPLC	Unusual Δ22 and 7-oxo groups
**Dinomyidae (family)**										
Dinomys branickii	Pacharana	VI	5β			X	Glycine	Δ22-7-OxoLCA	HPLC	Unusual Δ22 and 7-oxo groups
**Muridae (family)**										6α-hydroxylation unusual in mammals (also found in chevrotains and Suidae)
Acomys cahirinus	Cairo spiny mouse	VI	5β			X	Taurine, glycine	CA, CDCA	HPLC	Different than *Mus* species
Mus minutoides	African pygmy mouse	VI	5β			X	Taurine	β-MCA, α-MCA, CDCA	HPLC	
Mus musculus	Southeastern	VI	5β			X	Taurine	β-MCA, CA, α-MCA	HPLC	

Species		Bile salt class	5H	C_{27} alcohols	C_{27} acids	C_{24} acids	Conjugation	Major bile salts	Methodology	Comments
castaneus	Asian house mouse									
**Castoridae (family)**										
Castor canadensis	North American beaver	VI	5β			X	Glycine, taurine	UDCA, CDCA	HPLC	
**Chinchillidae (family)**										
Chinchilla sp.	Chinchilla	VI	5β			X	Taurine	CA, DCA	HPLC	
**Dasyproctidae (family)**										
Dasyprocta punctata	Central American agouti	VI	5β			X	Glycine, taurine	UDCA, CDCA, also some Δ22 bile acids	HPLC	Unusual Δ22 Bile acids
**Heterocephalinae (family)**										
Heterocephalus glober	Naked mole rat	VI	5β			X	Glycine, taurine	CDCA	HPLC	
**Caviidae (family)**										
Hydrochorus hydrochaeris	Capybara	VI	5β			X	Glycine	CDCA, 7-OxoLCA	HPLC	
Kerodon rupestris	Rock cavy	VI	5β			X	Taurine, glycine	7-oxoLCA, CDCA, UDCA	HPLC	CA not detected
Myocaster coypus	Nutria	VI	5β			X	Glycine, taurine	UDCA, 7-oxoLCA, CDCA	HPLC	
