# Supplementary material for: Evolutionary diversity of bile salts in reptiles and mammals, including analysis of ancient human and extinct giant ground sloth coprolites
Source: BMC Evol Biol. 2010 May 6;10:133. doi: 10.1186/1471-2148-10-133 (PMC2886068; doi:10.1186/1471-2148-10-133)
Supplement: Additional file 3 — Annotated bile salt profiles of reptiles and mammals. Examples of bile salt profiles (annotated ESI/MS/MS and GC/MS spectra) for reptiles and mammals are included. [file 1471-2148-10-133-S3.PDF]

ADDITIONAL FILE 3

Annotated bile salt profiles

EVOLUTIONARY DIVERSITY OF BILE SALTS IN REPTILES AND MAMMALS, INCLUDING ANALYSIS OF HUMAN AND EXTINCT GIANT GROUND SLOTH COPROLITES

Lee R. Hagey, Nicolas Vidal, Alan F. Hofmann, and Matthew D. Krasowski

ESI/MS/MS and GC/MS Analyses of biliary bile salts from reptiles and mammals

ESI/MS/MS spectra are annotated with probable matches based on *m/z* ratios and corroborations with other analyses (e.g., HPLC, GC/MS, NMR, thin-layer chromatography). The major bile salt(s) of each species are highlighted in blue.

Abbreviations: CA, cholic acid (3α,7α,12α-trihydroxy-5β-cholan-24-oic acid); CDCA, chenodeoxycholic acid (3α,7α-dihydroxy-5β-cholan-24-oic acid); DCA, deoxycholic acid (3α,12α-dihydroxy-5β-cholan-24-oic acid); ESI/MS/MS, electrospray ionization-tandem mass spectrometry.

Supplemental Figures 3A-DD

Animals with type II bile salt profiles (presence of C_{27} bile alcohols and C_{27} bile acids, each comprising at least 10% of total biliary bile salts)

A) *Gopherus agassizii* (California desert tortoise) bile – ESI/MS/MS

The main biliary bile salts from this turtle are C_{27} bile acids (peaks F-I) but about 30% of total biliary bile salts are C_{27} bile alcohols (peaks A, D, and E).

B) *Gopherus agassizii* (California desert tortoise) bile – ESI/MS/MS, focus on bile alcohol fraction (parents of sulfates)

This analysis highlights the bile alcohol sulfates in the California desert tortoise. Some peaks consistent with singly- or doubly-unsaturated bile alcohol sulfates are also indicated.

Animals with type IV bile salt profiles (>90% C_{27} bile acids)

C) *Malaclemys terrapin* (diamondback terrapin) bile – ESI/MS/MS

The biliary bile salts from this turtle contains > 90% C_{27} acids. By separate HPLC analyses, these C_{27} bile acids are known to be 22-hydroxylated, a modification to C_{27} bile acids so far unique to species from Testudines.

D) *Malaclemys terrapin* (diamondback terrapin) bile – ESI/MS/MS, focus on bile alcohol fraction (parents of sulfates)

This analysis highlights the minor population of bile alcohol sulfates in the diamondback terrapin, revealing the presence of tetra-, penta-, and hexa-hydroxylated C_{27} bile alcohols that collectively account for less than 5% of total biliary bile salts.

E) *Varanus varius* (lace monitor) bile – ESI/MS/MS

The bile of the lace monitor, like all other species from Varanidae analyzed, is dominated by C_{27} bile acids, especially varanic acid ($3\alpha,7\alpha,12\alpha,24R$-tetrahydroxy-5β-cholestan-27-oic acid). A minor population of C_{26} and C_{27} bile alcohols is also evident.

F) *Varanus varius* (lace monitor) bile – ESI/MS/MS, focus on bile alcohol fraction (parents of sulfates)

This analysis highlights the minor population of C_{26} and C_{27} bile alcohol sulfates that accounts for less than 5% of total biliary bile salts in the lace monitor.

G) *Varanus melinus* (quince monitor) bile – ESI/MS/MS

Like the lace monitor, the main biliary bile salt of the quince monitor is the C_{27} bile acid varanic acid.

H) *Varanus melinus* (quince monitor) bile – ESI/MS/MS, focus on sulfated bile salt fraction (parents of sulfates)

This analysis highlights the complex mixture of sulfated bile acids and alcohols that accounts for less than 5% of total biliary bile salts in the quince monitor.

I) *Varanus bengalensis* (Bengal monitor) bile – ESI/MS/MS

Like the lace and quince monitors, the main biliary bile salt of the Bengal monitor is the C_{27} bile acid varanic acid.

J) *Varanus bengalensis* (Bengal monitor) bile – ESI/MS/MS, focus on C_{24} bile acids

This analysis shows the minor fraction of C_{24} acids present in the bile of the Bengal monitor in addition to C_{27} bile acids.

K) *Egernia depressa* (pygmy spiny-tailed skink) bile – ESI/MS/MS

The bile of the pygmy spiny-tailed skink is dominated by C_{27} bile acids, with a minor population of C_{26} and C_{27} bile alcohols.

L) *Egernia depressa* (pygmy spiny-tailed skink) bile – ESI/MS/MS, focus on sulfated bile salt fraction (parents of sulfates)

This analysis highlights the complex mixture of sulfated bile acids and alcohols that accounts for less than 5% of total biliary bile salts in the pygmy spiny-tailed skink.

M) *Caiman crocodilus* (spectacled caiman) bile – ESI/MS/MS

The bile of the spectacled caiman, like other species within Crocodylia, is mainly comprised of C_{27} bile acids (peaks F and G), with minor fractions of C_{24} bile acids, C_{26} bile alcohols, and C_{27} bile alcohols.

N) *Caiman crocodilus* (spectacled caiman) bile – ESI/MS/MS, focus on sulfated bile salt fraction (parents of sulfates)

This analysis highlights the complex mixture of sulfated bile acids and alcohols in the bile of the spectacled caiman, including unusual C_{29} bile acids.

<u>Animals with type V bile salt profiles (presence of C_{24} and C_{27} bile acids, each comprising at least 10% of total biliary bile salts)</u>

O) *Galago senegalensis* (lesser bushbaby) bile – ESI/MS/MS

This analysis focuses on the taurine-conjugated C_{24} bile acids found in the bile of the lesser bushbaby, including the common bile acid CDCA (peak A) and CA (peak B).

P) *Galago senegalensis* (lesser bushbaby) bile – ESI/MS/MS, focus on bile alcohols (parents of sulfates)

This analysis highlights the complex mixture of C_{26} and C_{27} bile alcohols in the bile of the lesser bushbaby, and also the presence of the plant sterol β-sitosterol.

<u>Animals with type VI bile salt profiles (>90% C_{24} bile acids)</u>

Q) *Uromastyx acanthinura* (North African spiny-tailed lizard) bile – ESI/MS/MS

The major bile acids of the North African spiny-tailed lizard are tauro-5α-CA (tauroallo-CA) and tauro-5α-DCA (tauroallo-DCA) as confirmed by separate HPLC analyses using reference standards.

R) *Uromastyx acanthinura* (North African spiny-tailed lizard) bile – ESI/MS/MS, focus on sulfated bile salt fraction (parents of sulfates)

This analysis highlights the mixture of sulfated C_{27} bile alcohols in the bile of the North African spiny-tailed lizard. These account for much less than 5% of the total biliary bile salts.

S) *Enhydris plumbea* (plumbeous water snake) bile – ESI/MS/MS

The major bile acids of the plumbeous water snake are tauro-23*R*-hydroxy-CA and tauro-CA as confirmed by separate HPLC analyses using reference standards. A small peak with an *m/z* ratio consistent with a penta-hydroxy taurine-conjugated C_{24} bile acids is evident. The exact identity of this compound (including the location of the hydroxyl groups) is unknown.

T) *Enhydris plumbea* (plumbeous water snake) bile – ESI/MS/MS, focus on sulfated bile salt fraction (parents of sulfates)

This analysis highlights the mixture of sulfated C_{27} bile alcohols in the bile of the plumbeous water snake. These account for much less than 5% of the total biliary bile salts.

U) *Naja haje* (Egyptian banded cobra) bile – ESI/MS/MS

The major primary bile acid of the Egyptian banded cobra plumbeous is tauro-CA, which comprises >98% of biliary bile salts.

V) *Tragulus javanicus* (lesser Malay chevrotain) bile – ESI/MS/MS

The major bile acids of the lesser Malay chevrotain are glycine-conjugated CA and hyocholic acid.

W) *Tragulus javanicus* (lesser Malay chevrotain) bile – ESI/MS/MS

This analysis highlights the taurine-conjugated bile acids of the lesser Malay chevrotain.

X) *Tragulus javanicus* (lesser Malay chevrotain) bile – ESI/MS/MS

This analysis highlights the mixture of sulfated C_{26} and C_{27} bile alcohols in the bile of the plumbeous water snake. These account for much less than 5% of the total biliary bile salts. The plant sterol β-sitosterol is also detected.

Y) *Choloepus hoffmanni* (Hoffmann's two-toed sloth) feces – ESI/MS/MS

This analysis focuses on the unconjugated bile acids in the feces of Hoffman's two-toed sloth. Lithocholic acid and CDCA are detected along with bile acids containing double bonds and/or oxo groups.

Z) *Choloepus hoffmanni* (Hoffmann's two-toed sloth) feces – ESI/MS/MS

This analysis focuses on the glycine-conjugated bile acids in the feces of Hoffman's two-toed sloth.

AA) *Choloepus hoffmanni* (Hoffmann's two-toed sloth) feces – ESI/MS/MS

This analysis highlights the complex mixture of plant sterols (campesterol and β-sitosterol) and sulfated C_{27} bile alcohols in the feces of the Hoffman's two-toed sloth. The presence of multiple plant sterols is consistent with an herbivorous diet.

BB) *Homo sapiens* (human) coprolite – GC/MS analysis, total ion chromatogram

This analysis is of a human coprolite sample (~8,000 year old) from Danger Cave Archaeological Site in Utah. Each labeled peak was verified by comparison with fragmentation spectrum of appropriate reference standard.

CC) *Nothrotherium shastense* (Shasta ground sloth) coprolite – ESI/MS/MS

Analysis of bile acids isolated from Shasta ground sloth coprolite sample (> 11,000 years old) from Rampart Cave in Arizona are shown. Spectra consistent with glycine-conjugated C_{24} bile acids are identified. Smaller peaks consistent with singly- and doubly-unsaturated analogs of the main glycine-conjugated bile acids are also present and labeled.

DD) *Nothrotherium shastense* (Shasta ground sloth) – ESI/MS/MS

This analysis highlights the complex mixture of plant sterols (campesterol, stigmastanol, stigmasterol, and β-sitosterol) and sulfated C_{27} bile alcohols in the Shasta ground sloth coprolite. The peaks labeled as "I" may correspond to trihydroxylated plant sterols. The presence of multiple plant sterols is consistent with an herbivorous diet.

Suppl. Fig. 3A
Gopherus agassizii
(California desert tortoise)

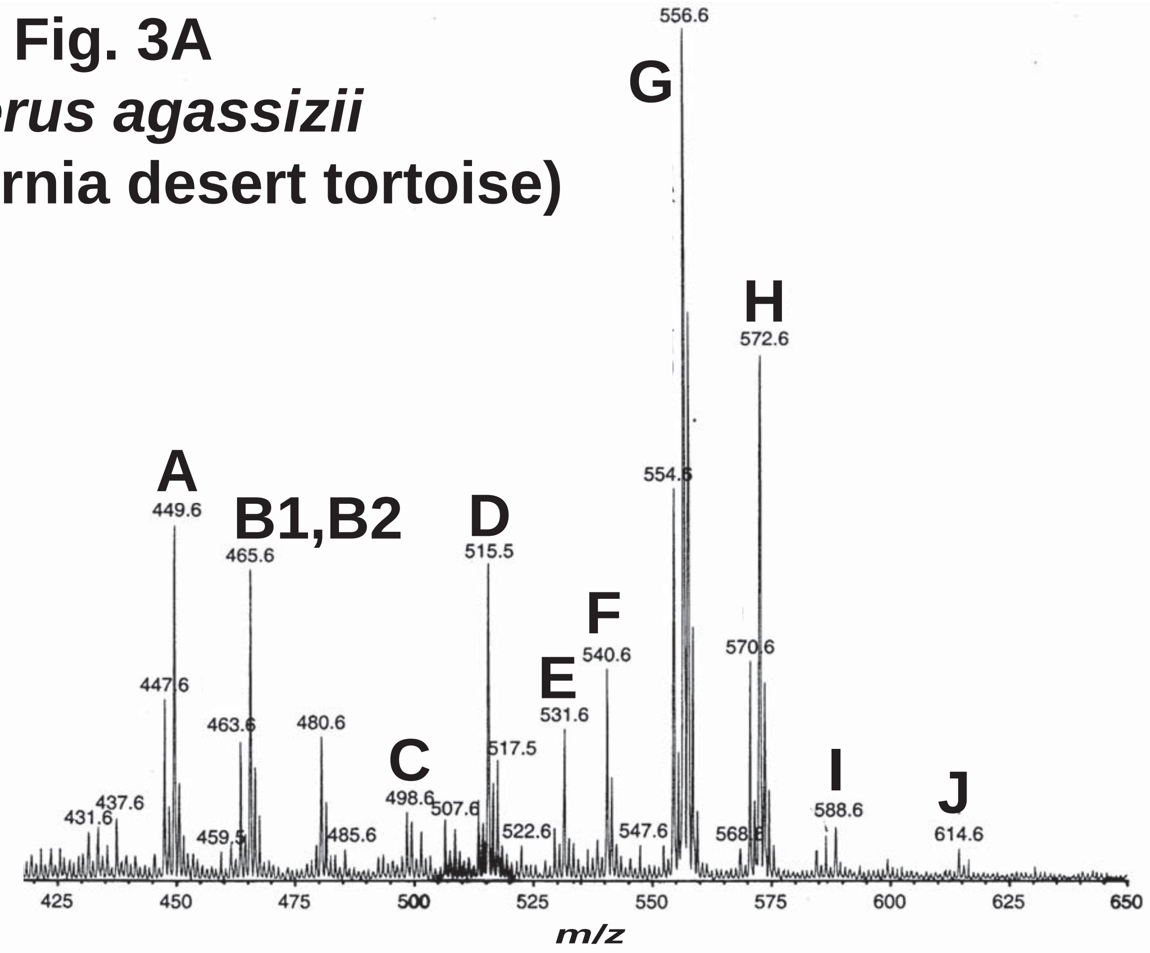

	Class	# of Hydroxyls	Conjugation	Double bonds (if any)	Adducts (if any)
A	C_{27} alcohol	3			
B1	C_{27} acid	4		1	
B2	C_{27} alcohol	1	Sulfate		
C	C_{24} acid	1	Taurine		
D*	C_{27} alcohol	4	Sulfate		
E	C_{27} alcohol	5	Sulfate		
F	C_{27} acid	2	Taurine		
G	C_{27} acid	3	Taurine		
H	C_{27} acid	4	Taurine		
I	C_{27} acid	5	Taurine		
J	C_{27} acid	3	Taurine		NaCl

* Note: The peak at *m/z*=465.6 is for both B1 and B2

Suppl. Fig. 3B
Gopherus agassizii
(California desert tortoise)

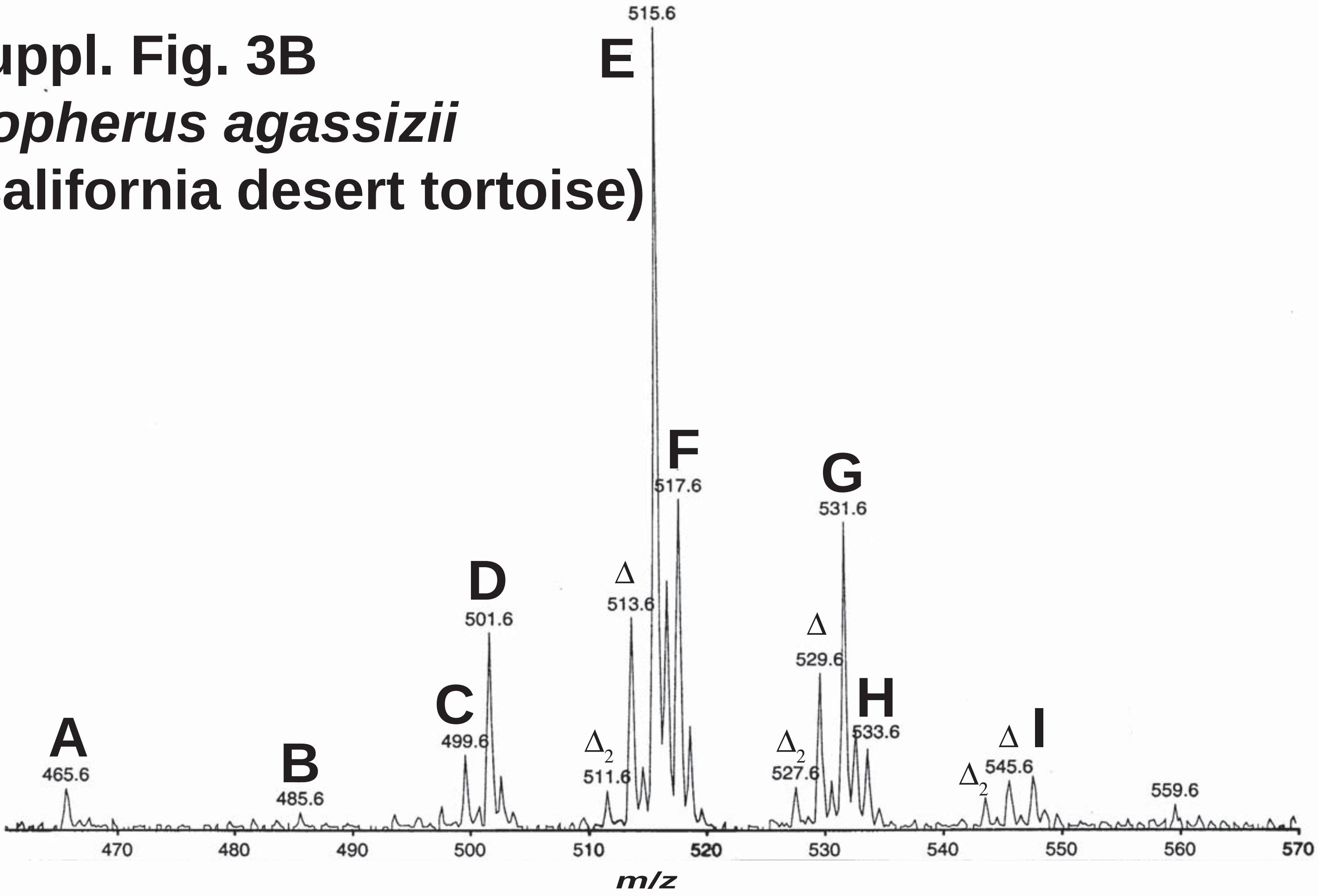

	Class	# of Hydroxyls	Double bonds (if any)	Conjugation
A	C_{27} alcohol	1	1	Sulfate
B	C_{26} alcohol	3		Sulfate
C	C_{27} alcohol	3		Sulfate
D	C_{26} alcohol	4		Sulfate
E	C_{27} alcohol	4		Sulfate
F*	C_{26} alcohol	5		Sulfate
G*	C_{27} alcohol	5		Sulfate
H	C_{26} alcohol	6		Sulfate
I*	C_{27} alcohol	6		Sulfate

*** Peaks with *m/z* ratios consistent with compounds F, G, and I having one (Δ) or two (Δ_2) double bonds are also indicated.**

Suppl. Fig. 3C
Malaclemys terrapin (diamondback terrapin)

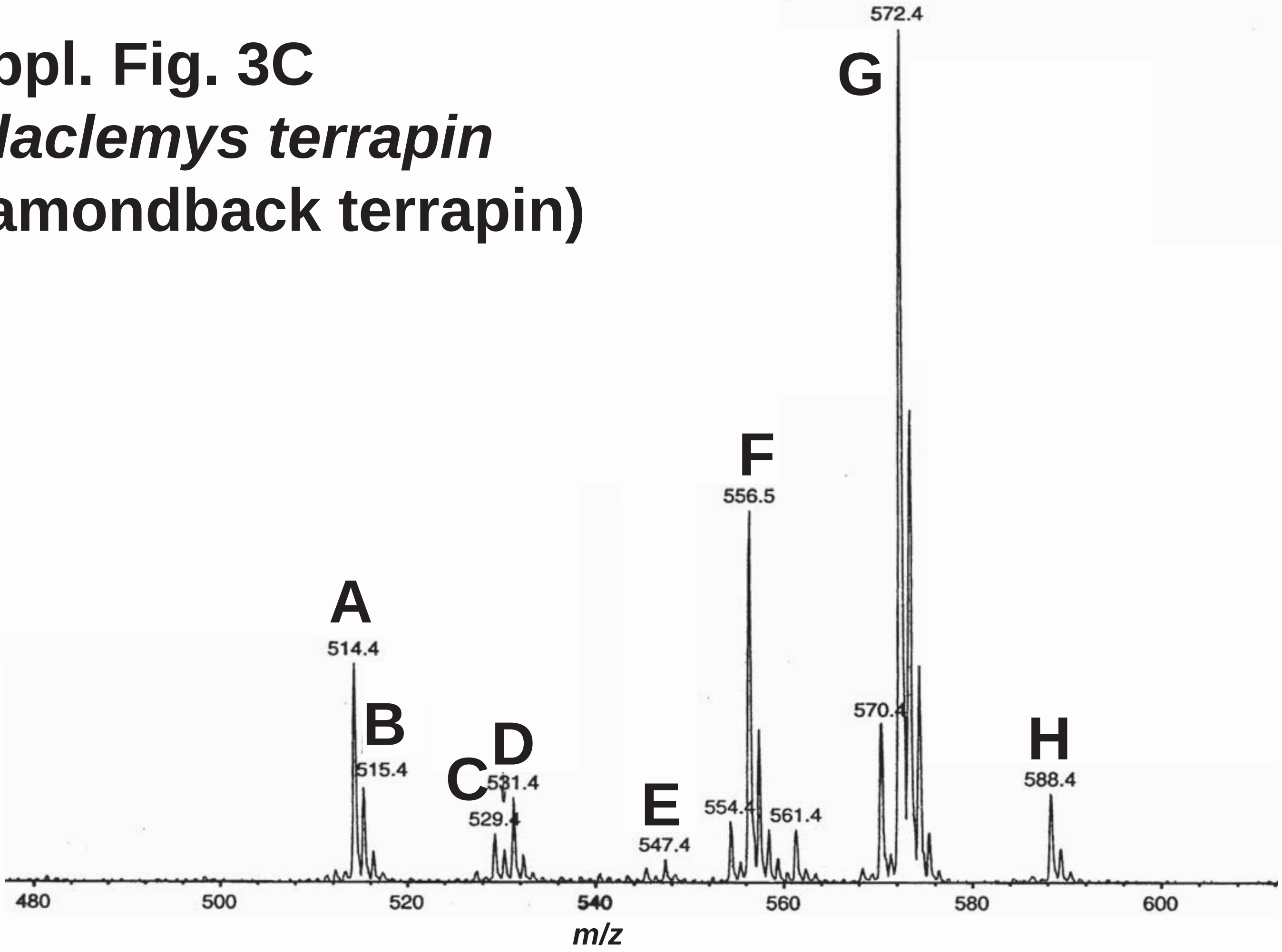

	Class	# of Hydroxyls	Conjugation
A[1]	C_{24} acid	3	Taurine
B	C_{27} alcohol	4	Sulfate
C	C_{24} acid	4	Taurine
D	C_{27} alcohol	5	Sulfate
E	C_{27} alcohol	6	Sulfate
F[2]	C_{27} acid	3	Taurine
G[2]	C_{27} acid	4	Taurine
H	C_{27} acid	5	Taurine

[1] Taurocholic acid
[2] 22-Hydroxylated C_{27} bile acids

Suppl. Fig. 3D
Malaclemys terrapin
(diamondback terrapin)

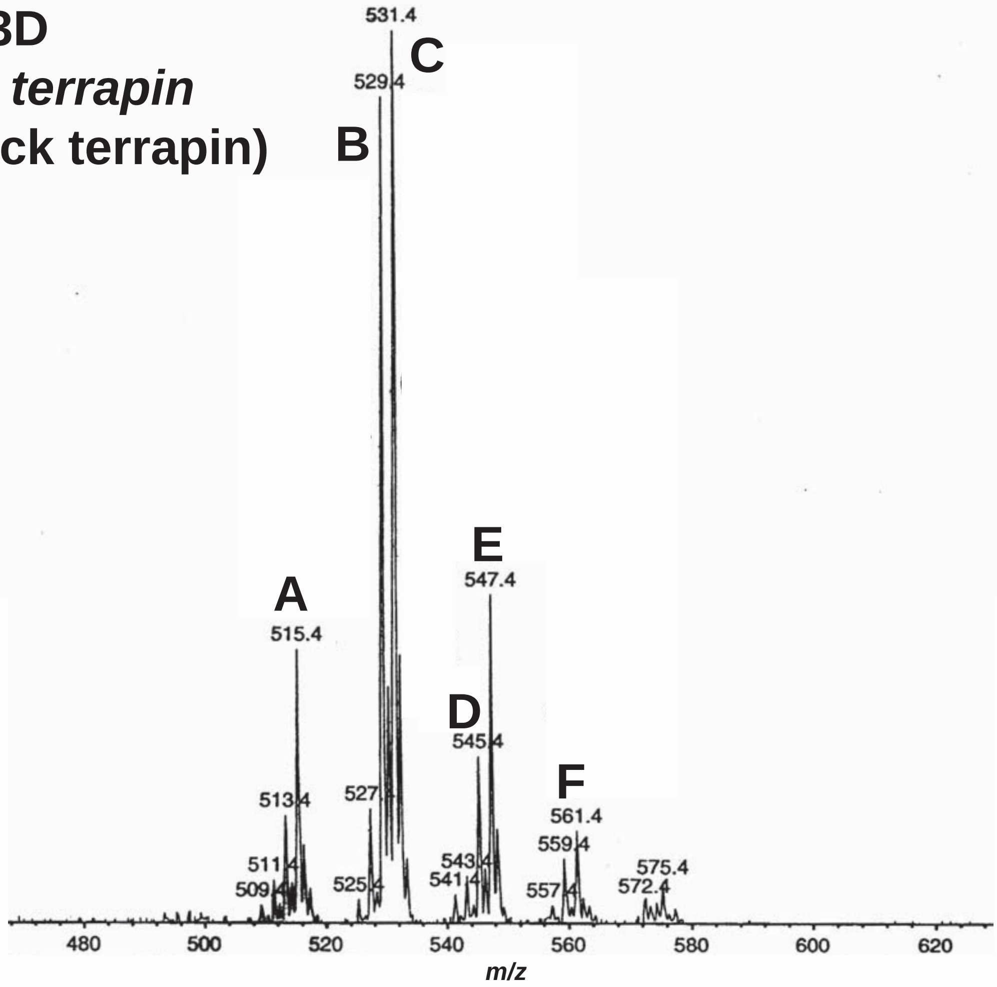

	Class	# of Hydroxyls	Conjugation
A	C_{27} alcohol	4	Sulfate
B	C_{27} acid	3	Sulfate
C	C_{27} alcohol	5	Sulfate
D	C_{27} acid	4	Sulfate
E	C_{27} alcohol	6	Sulfate
F	C_{27} acid	6	Sulfate

Suppl. Fig. 3E
Varanus varius
(lace monitor)

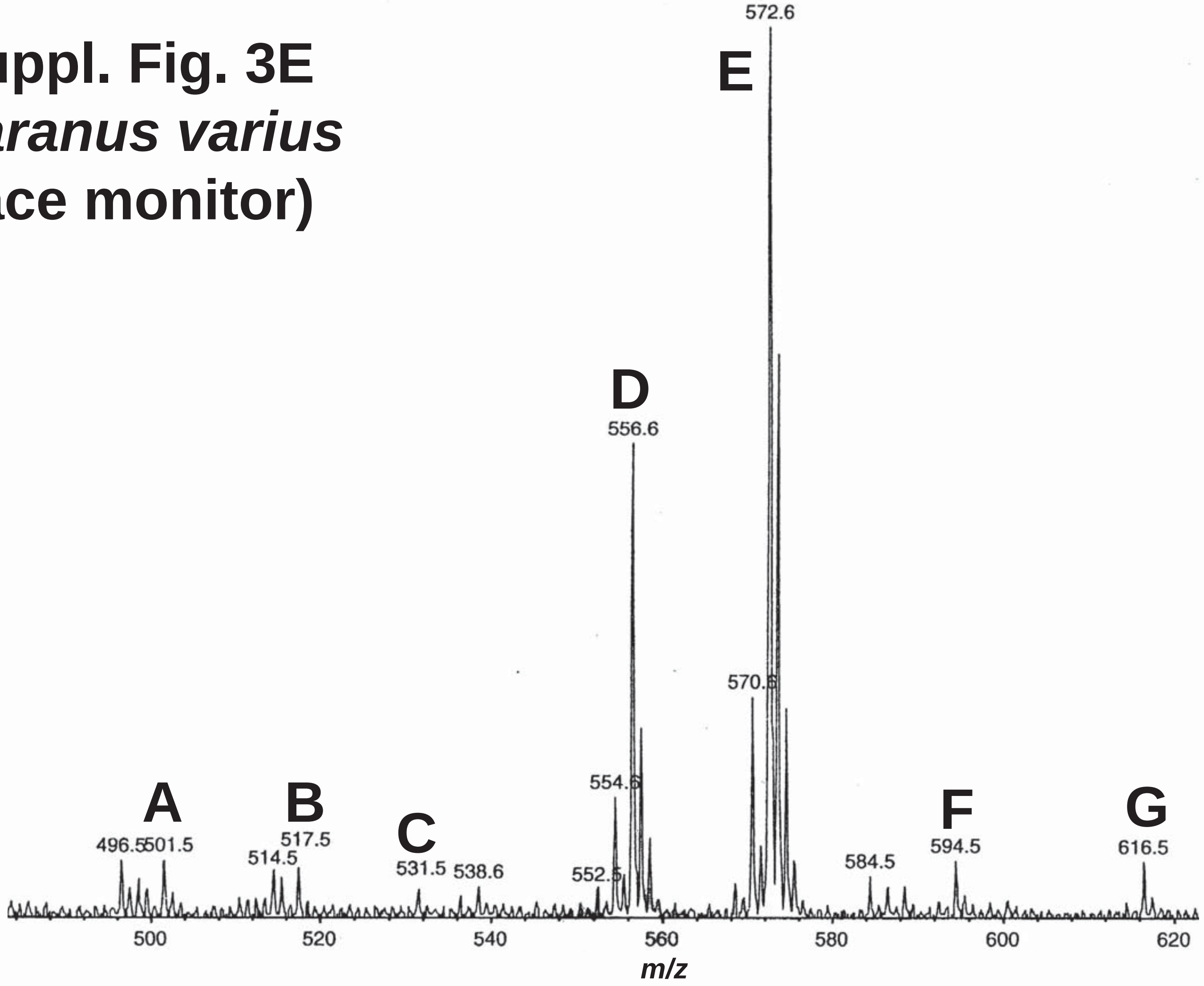

	Class	# of Hydroxyls	Conjugation	Adducts (if any)
A	C_{26} alcohol	4	Sulfate	
B	C_{26} alcohol	5	Sulfate	
C	C_{27} alcohol	5	Sulfate	
D	C_{27} acid	3	Taurine	
E*	C_{27} acid	4	Taurine	
F	C_{27} acid	4	Taurine	Na
G	C_{27} acid	4	Taurine	2Na

* Varanic acid

Suppl. Fig. 3F
Varanus varius
(lace monitor)

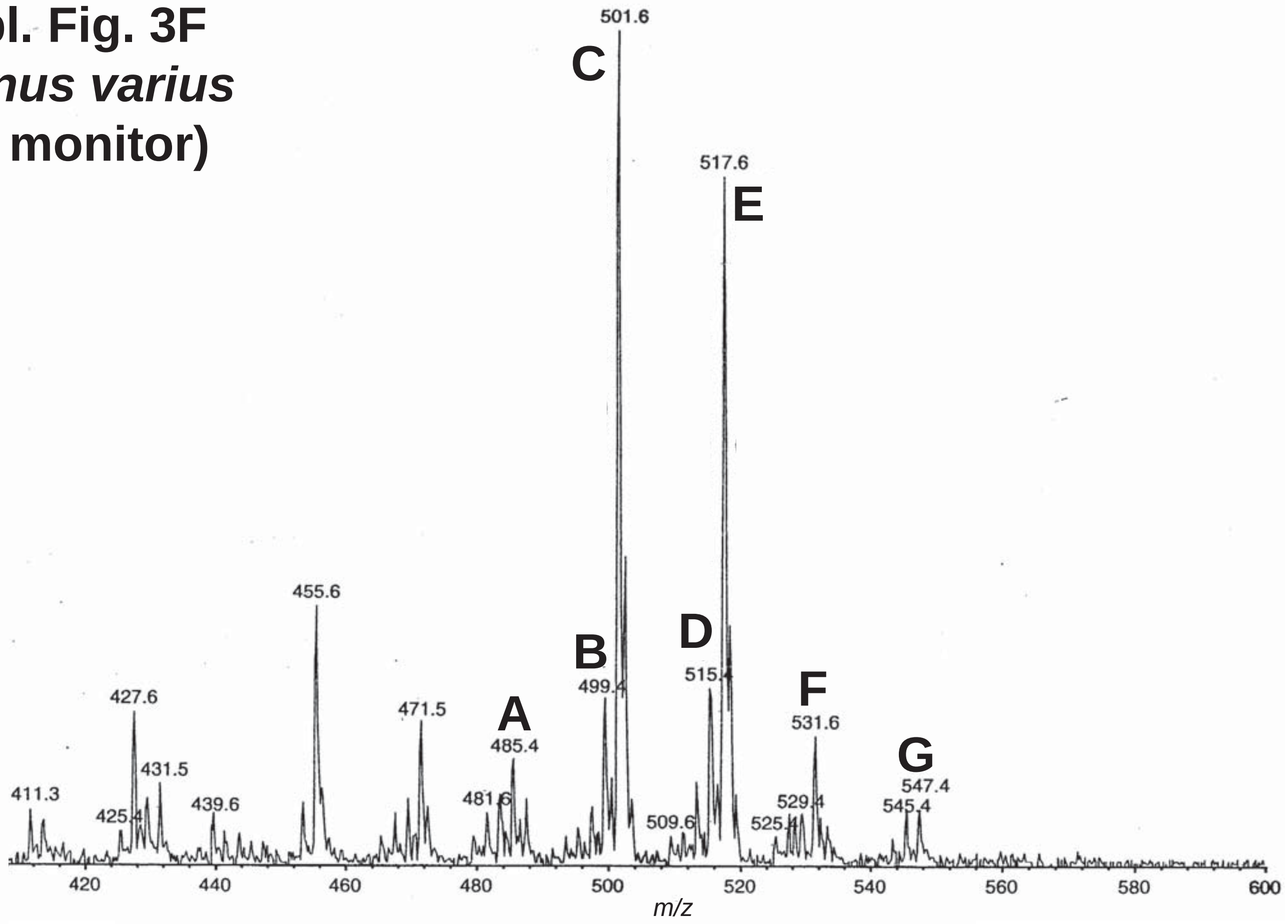

	Class	# of Hydroxyls	Conjugation
A	C_{26} alcohol	3	Sulfate
B	C_{27} alcohol	3	Sulfate
C	C_{26} alcohol	4	Sulfate
D	C_{27} alcohol	4	Sulfate
E	C_{26} alcohol	5	Sulfate
F	C_{27} alcohol	5	Sulfate
G	C_{27} alcohol	6	Sulfate

Suppl. Fig. 3G
Varanus melinus
(quince monitor)

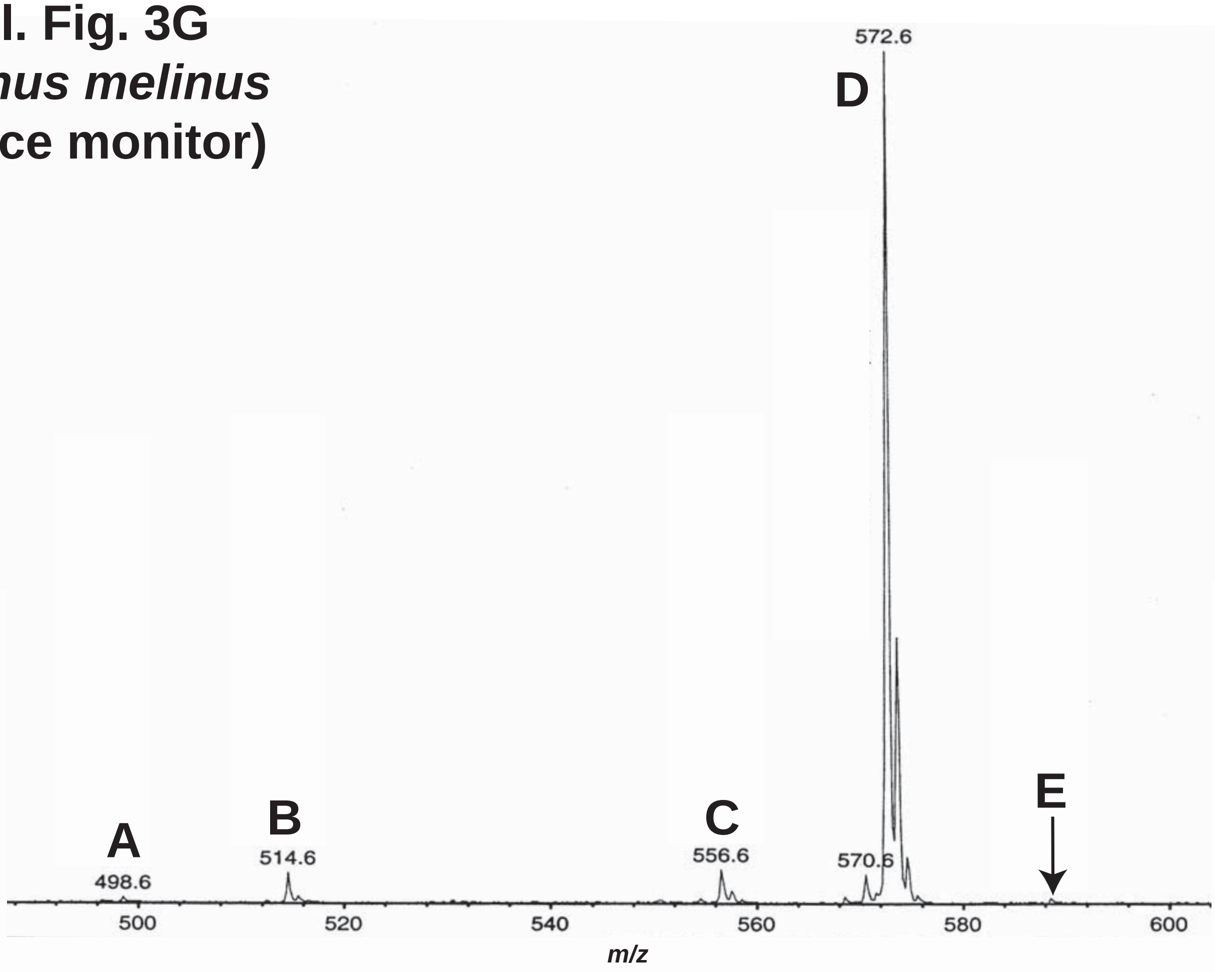

	Class	# of Hydroxyls	Conjugation
A	C_{24} acid	2	Taurine
B	C_{24} acid	3	Taurine
C	C_{27} acid	3	Taurine
D*	C_{27} acid	4	Taurine
E	C_{27} acid	5	Taurine

* Varanic acid

Suppl. Fig. 3H
Varanus melinus
(quince monitor)

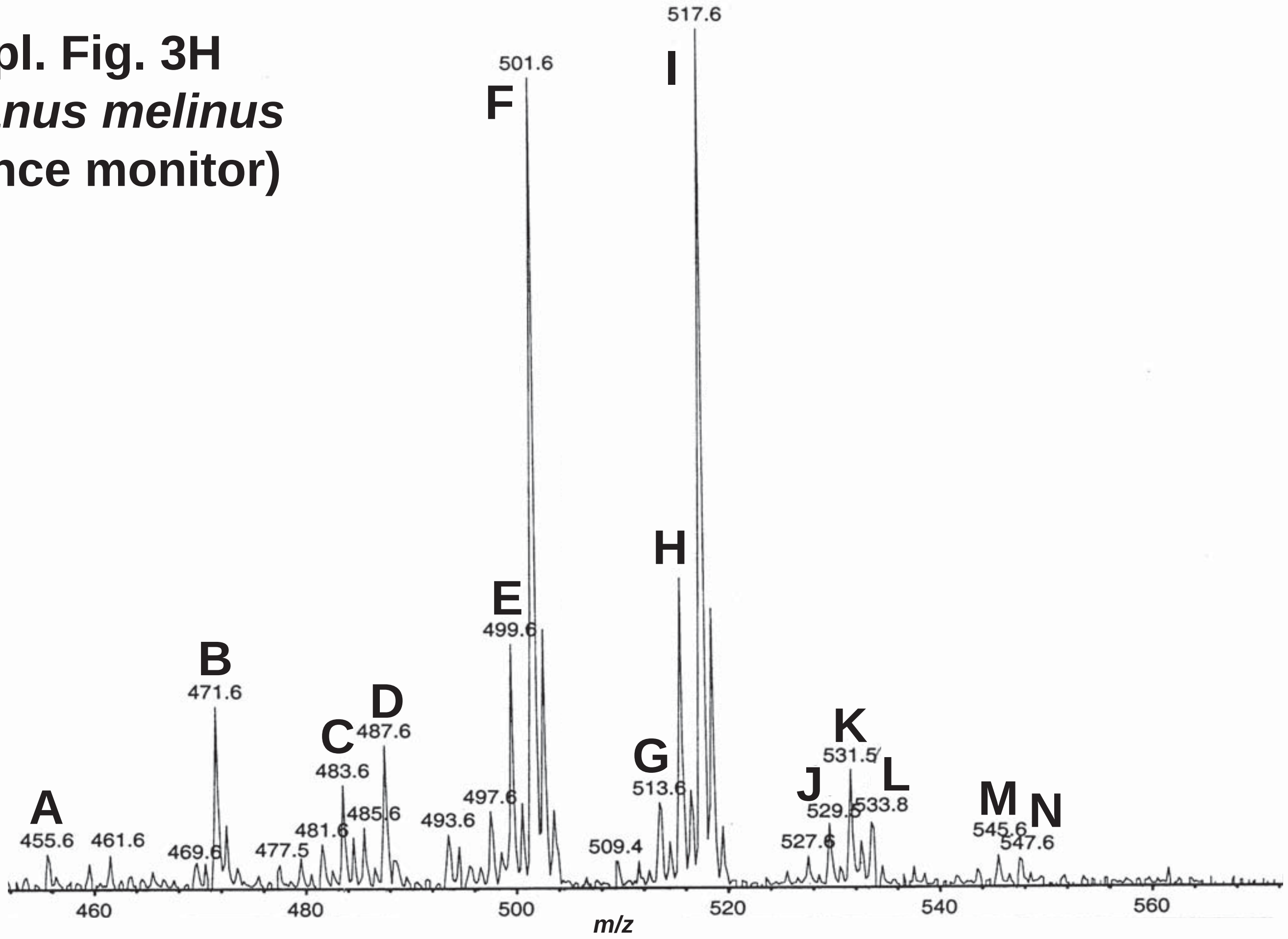

	Class	# of Hydroxyls	Conjugation
A	C_{24} acid	1	Sulfate
B	C_{24} acid	2	Sulfate
C	C_{27} alcohol	2	Sulfate
D	C_{24} acid	3	Sulfate
E	C_{27} alcohol	3	Sulfate
F	C_{26} alcohol	4	Sulfate
G	C_{27} acid	2	Sulfate
H	C_{27} alcohol	4	Sulfate
I	C_{26} alcohol	5	Sulfate
J	C_{27} acid	3	Sulfate
K	C_{27} alcohol	5	Sulfate
L	C_{26} alcohol	6	Sulfate
M	C_{27} acid	4	Sulfate
N	C_{27} alcohol	6	Sulfate

Suppl. Fig. 3I
Varanus bengalensis
(Bengal monitor)

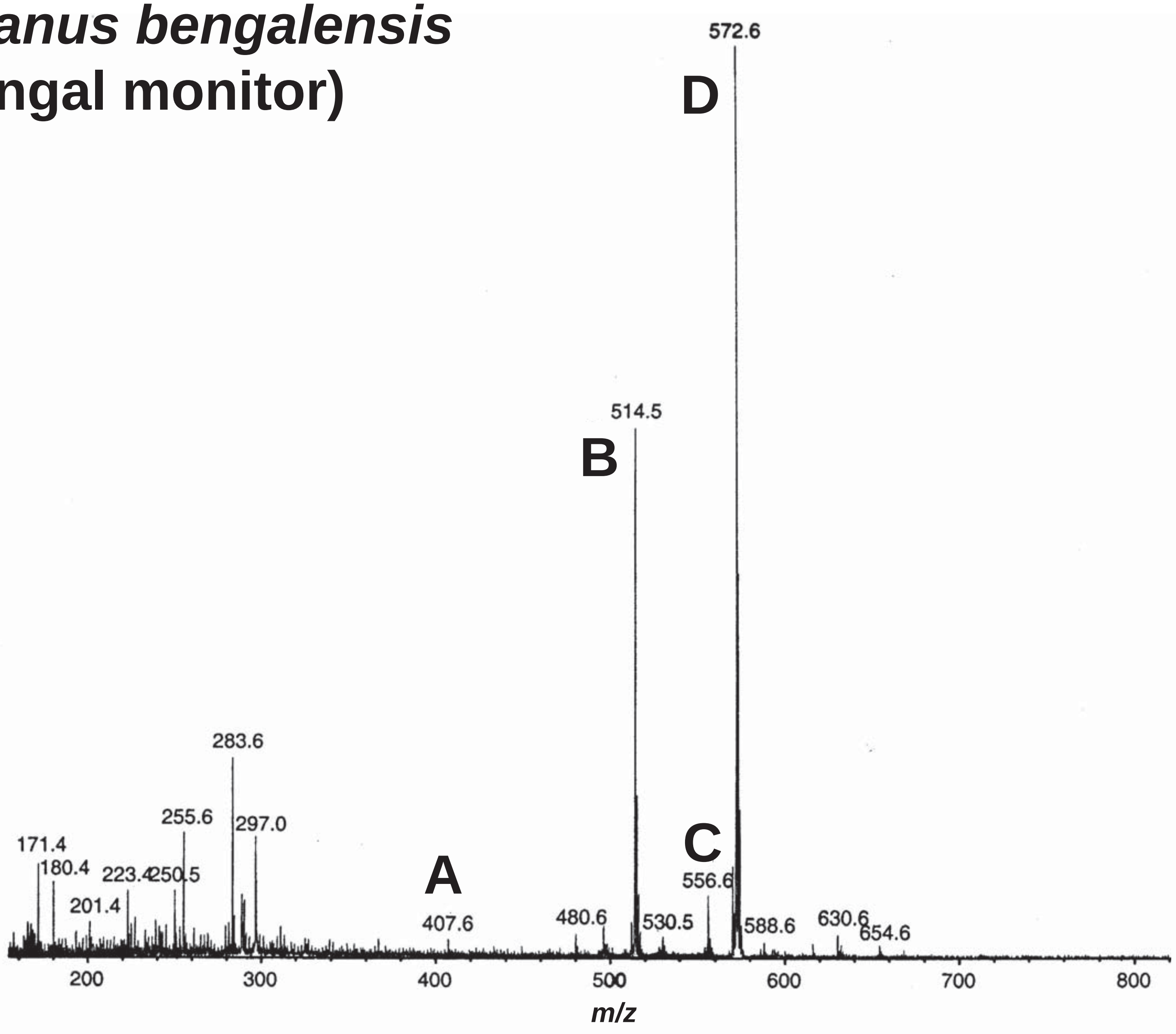

	Class	# of Hydroxyls	Conjugation (if any)
A	C_{24} acid	3	
B	C_{24} acid	3	Taurine
C	C_{27} acid	3	Taurine
D*	C_{27} acid	4	Taurine

* Varanic acid

Suppl. Fig. 3J
Varanus bengalensis
(Bengal monitor)

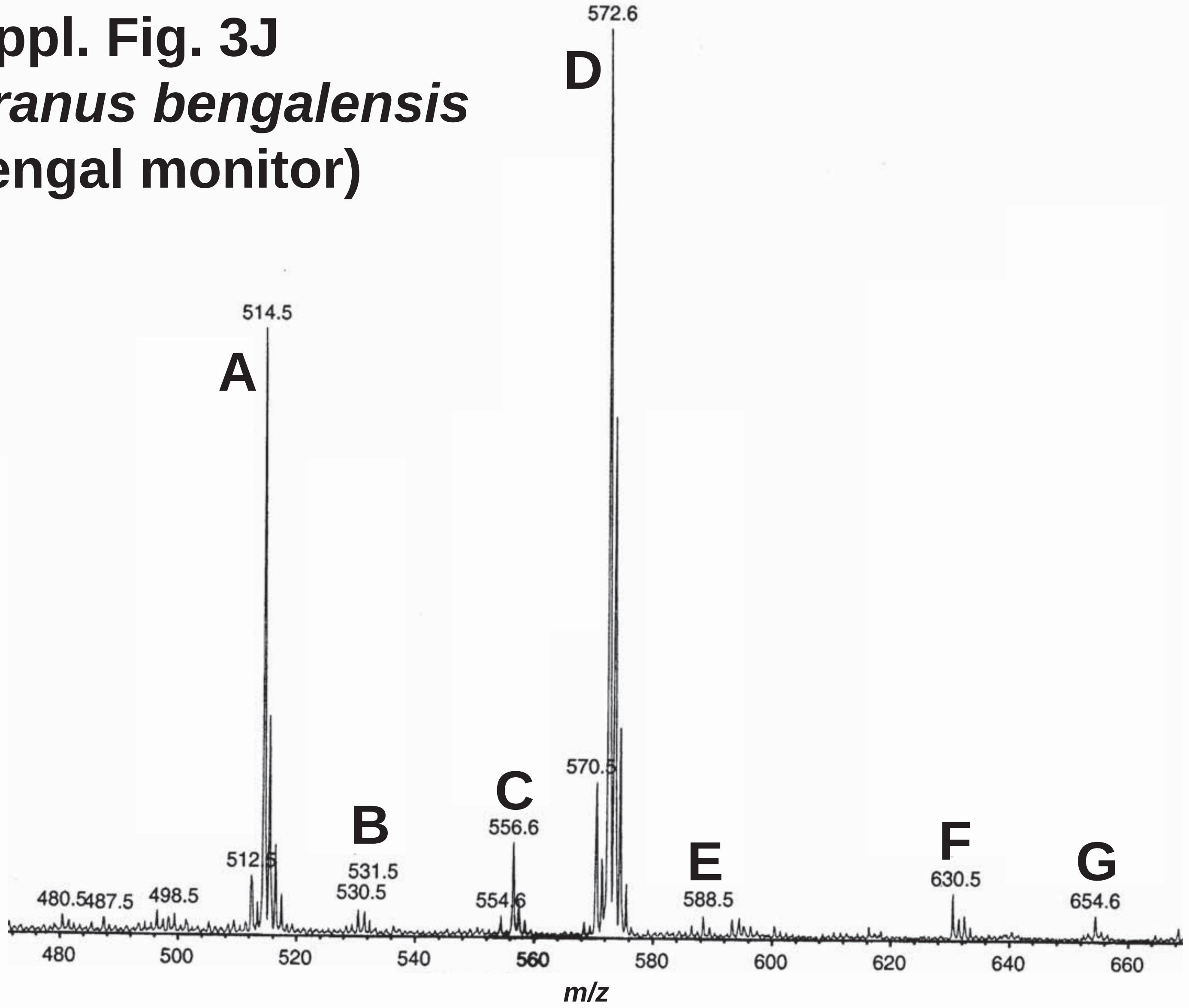

	Class	# of Hydroxyls	Conjugation	Adducts (if any)
A	C_{24} acid	3	Taurine	
B	C_{24} acid	4	Taurine	
C	C_{27} acid	3	Taurine	
D	C_{27} acid	4	Taurine	
E*	C_{27} acid	5	Taurine	
F	C_{27} acid	4	Taurine	NaCl
G	C_{27} acid	4	Taurine	NaCl•Na

* Varanic acid

Suppl. Fig. 3K
Egernia depressa (pygmy spiny-tailed skink)

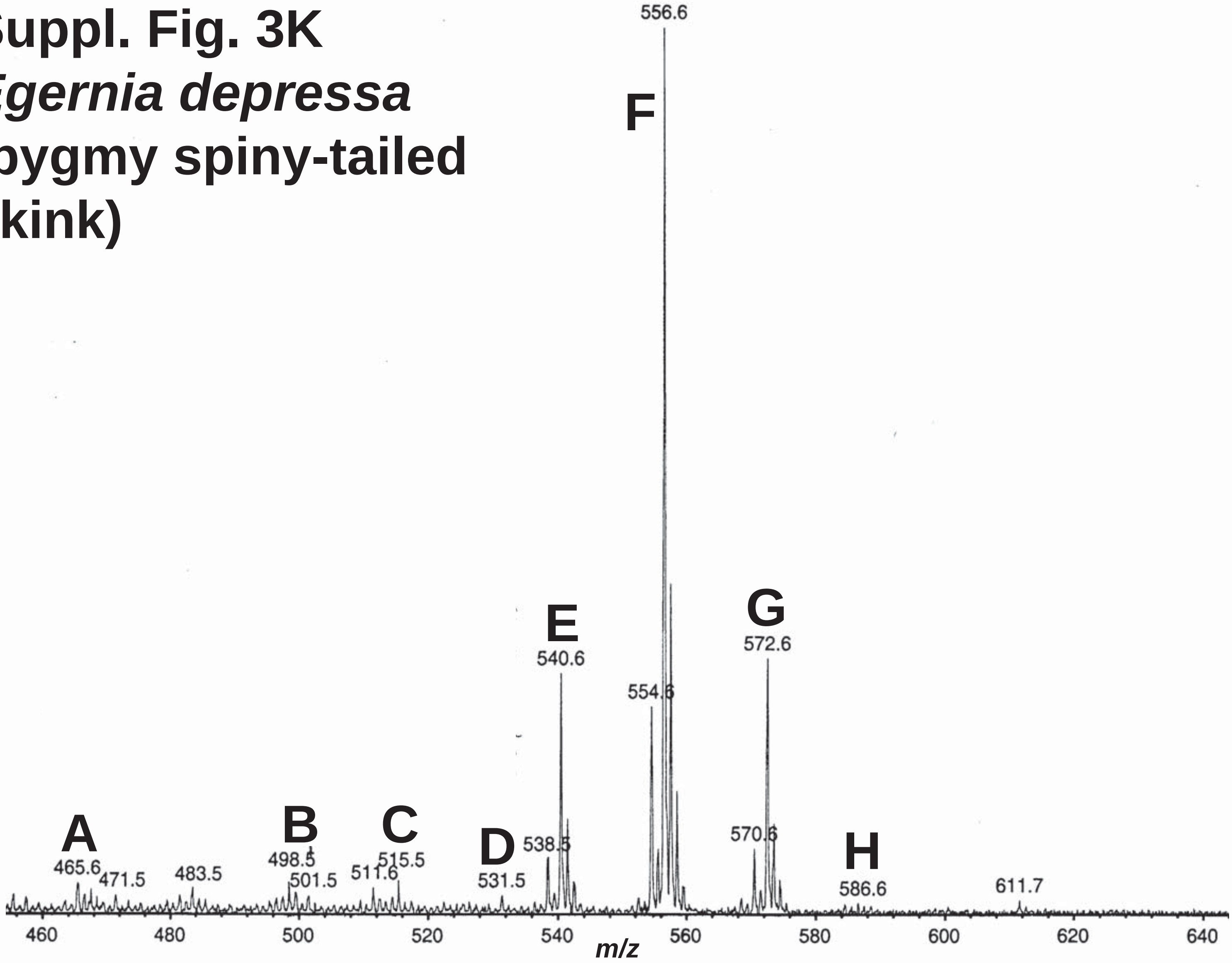

	Class	# of Hydroxyls	Conjugation
A	C_{27} alcohol	1	Sulfate
B	C_{26} alcohol	4	Sulfate
C	C_{27} alcohol	4	Sulfate
D	C_{27} alcohol	5	Sulfate
E	C_{27} acid	2	Taurine
F	C_{27} acid	3	Taurine
G	C_{27} acid	4	Taurine
H	C_{27} acid	5	Taurine

Suppl. Fig. 3L
Egernia depressa
(pygmy spiny tailed skink)

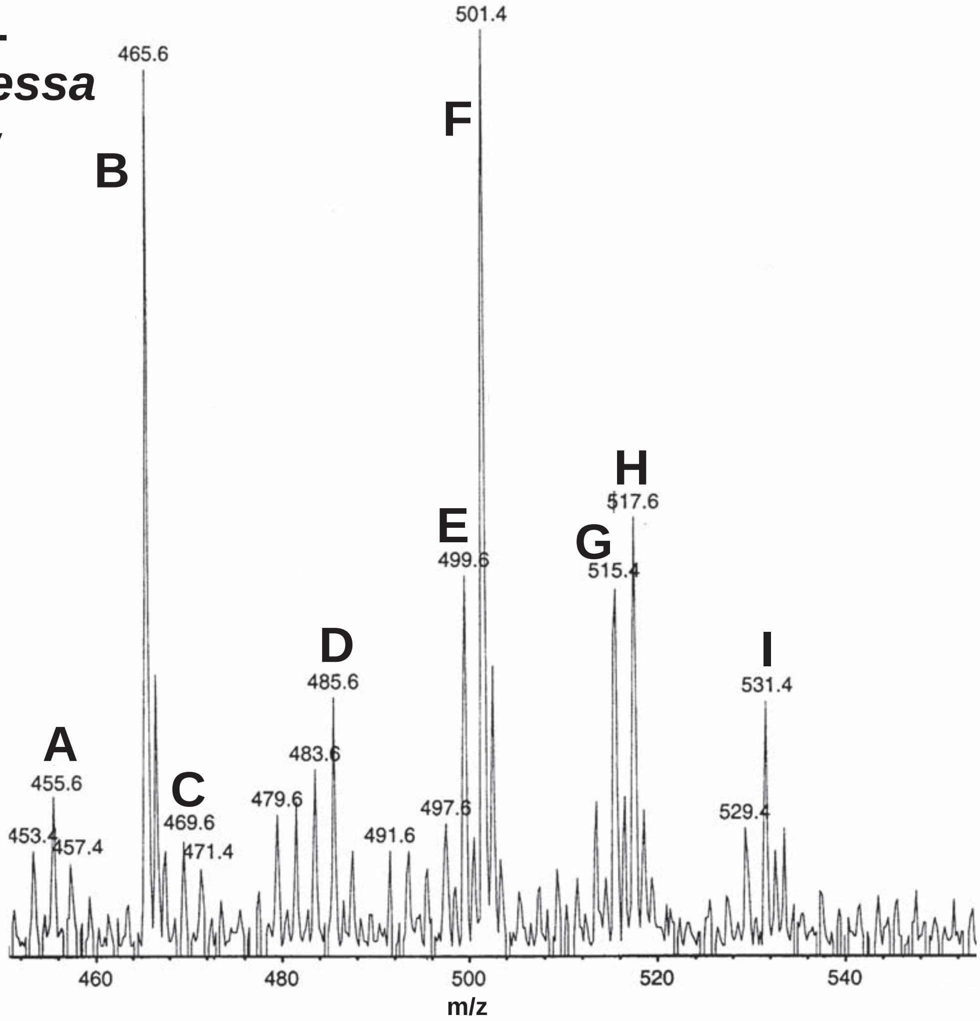

	Class	# of Hydroxyls	Double bonds (if any)	Conjugation
A	C_{24} acid	1		Sulfate
B	C_{27} alcohol	1	1	Sulfate
C	C_{24} acid	2		Sulfate
D	C_{26} alcohol	3		Sulfate
E	C_{27} alcohol	3		Sulfate
F	C_{26} alcohol	4		Sulfate
G	C_{27} alcohol	4		Sulfate
H	C_{26} alcohol	5		Sulfate
I	C_{27} alcohol	5		Sulfate

Suppl. Fig. 3M
Caiman crocodilus
(spectacled caiman)

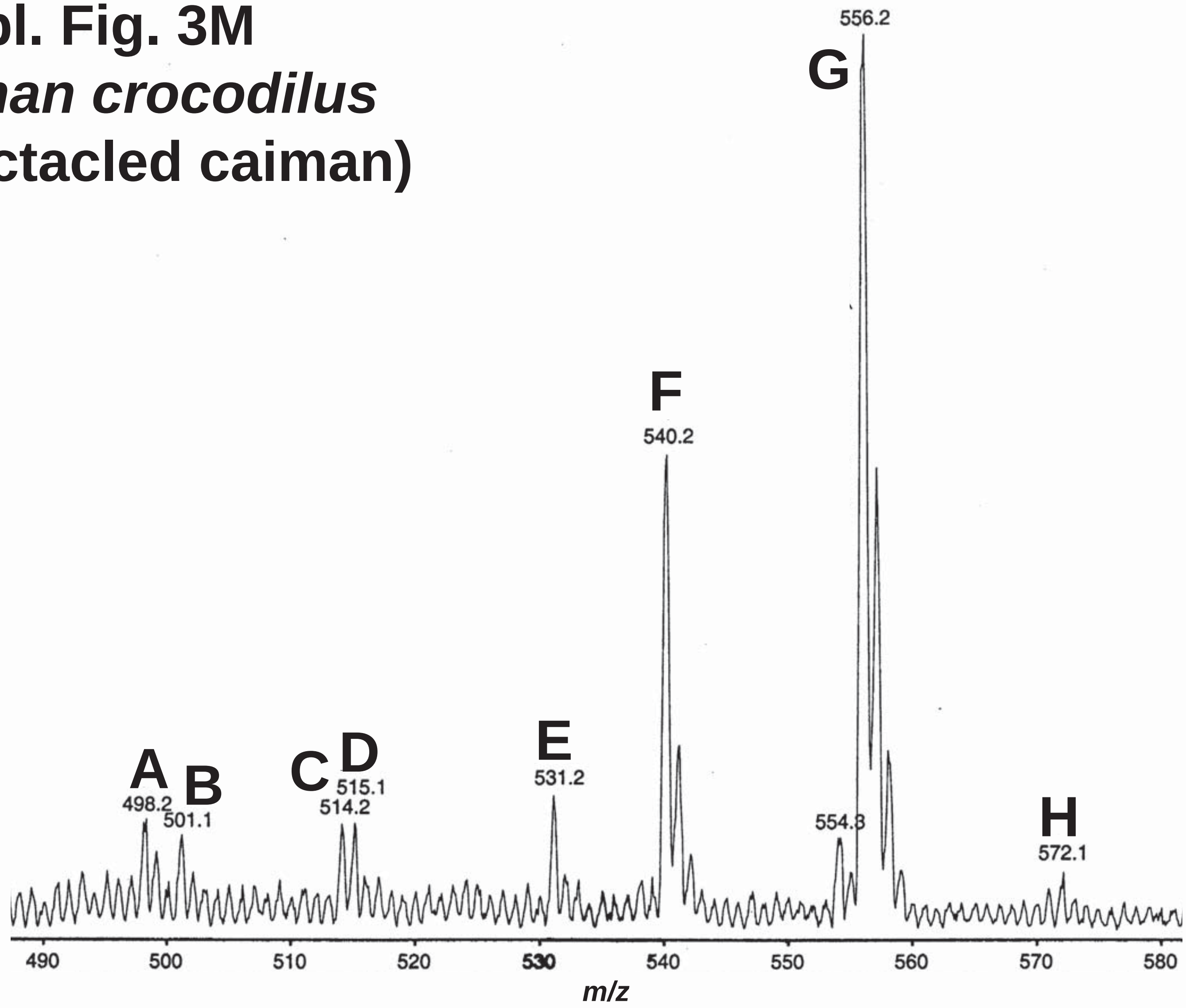

	Class	# of Hydroxyls	Conjugation
A	C_{24} acid	2	Taurine
B	C_{26} alcohol	4	Sulfate
C	C_{24} acid	3	Taurine
D	C_{27} alcohol	4	Sulfate
E	C_{27} alcohol	5	Sulfate
F	C_{27} acid	2	Taurine
G	C_{27} acid	3	Taurine
H	C_{27} acid	4	Taurine

Suppl. Fig. 3N
Caiman crocodilus
(spectacled
caiman)

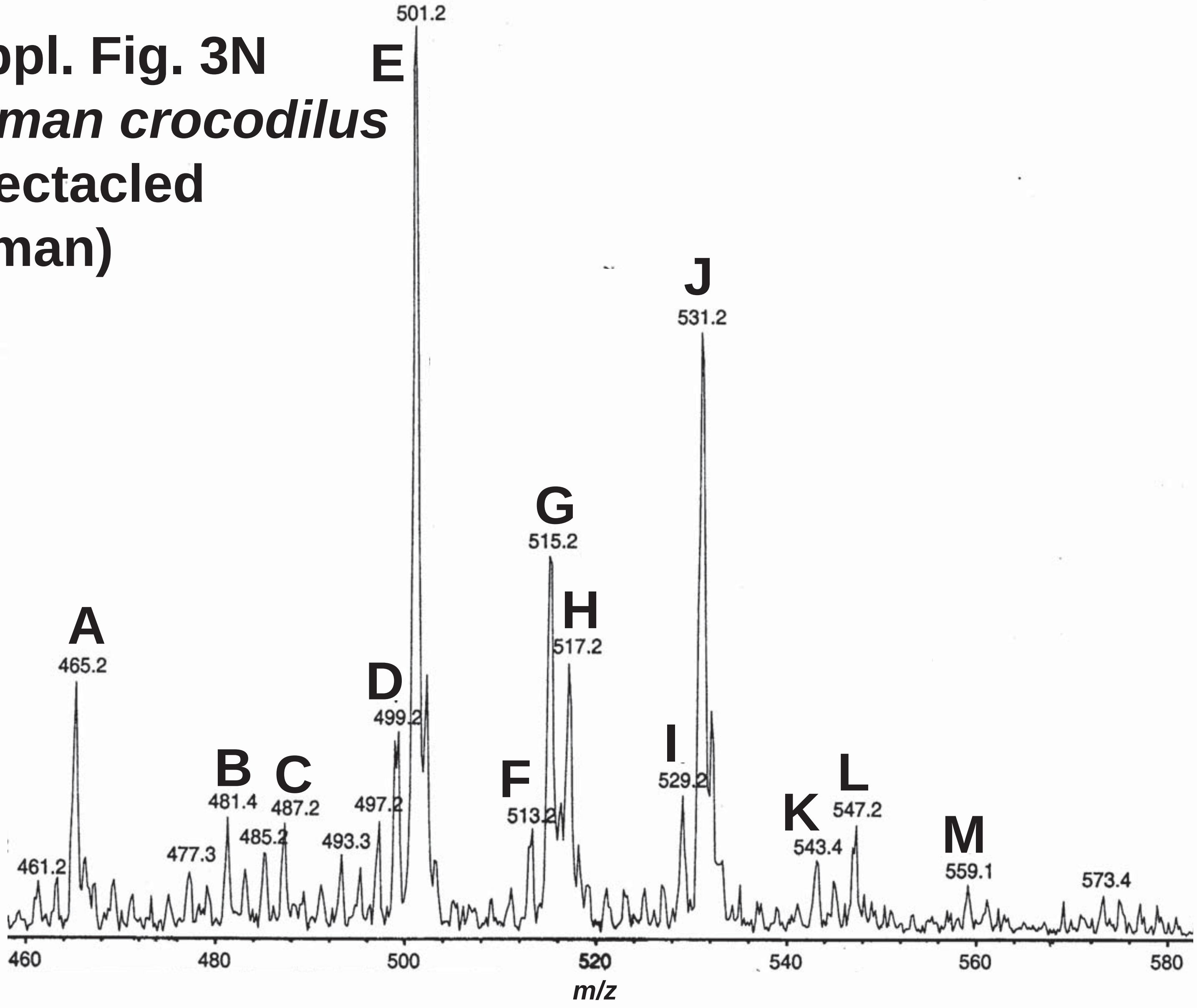

	Class	# of Hydroxyls	Double bonds (if any)	Conjugation
A	C_{27} alcohol	1	1	Sulfate
B	C_{27} alcohol	2	1	Sulfate
C	C_{24} acid	3		Sulfate
D	C_{27} alcohol	3		Sulfate
E	C_{26} alcohol	4		Sulfate
F	C_{27} acid	2		Sulfate
G	C_{27} alcohol	4		Sulfate
H	C_{26} alcohol	5		Sulfate
I	C_{27} acid	3		Sulfate
J	C_{27} alcohol	5		Sulfate
K	C_{29} acid	4		Sulfate
L	C_{27} alcohol	6		Sulfate
M	C_{29} acid	5		Sulfate

Suppl. Fig. 3O
Galago senegalensis
(lesser bushbaby)

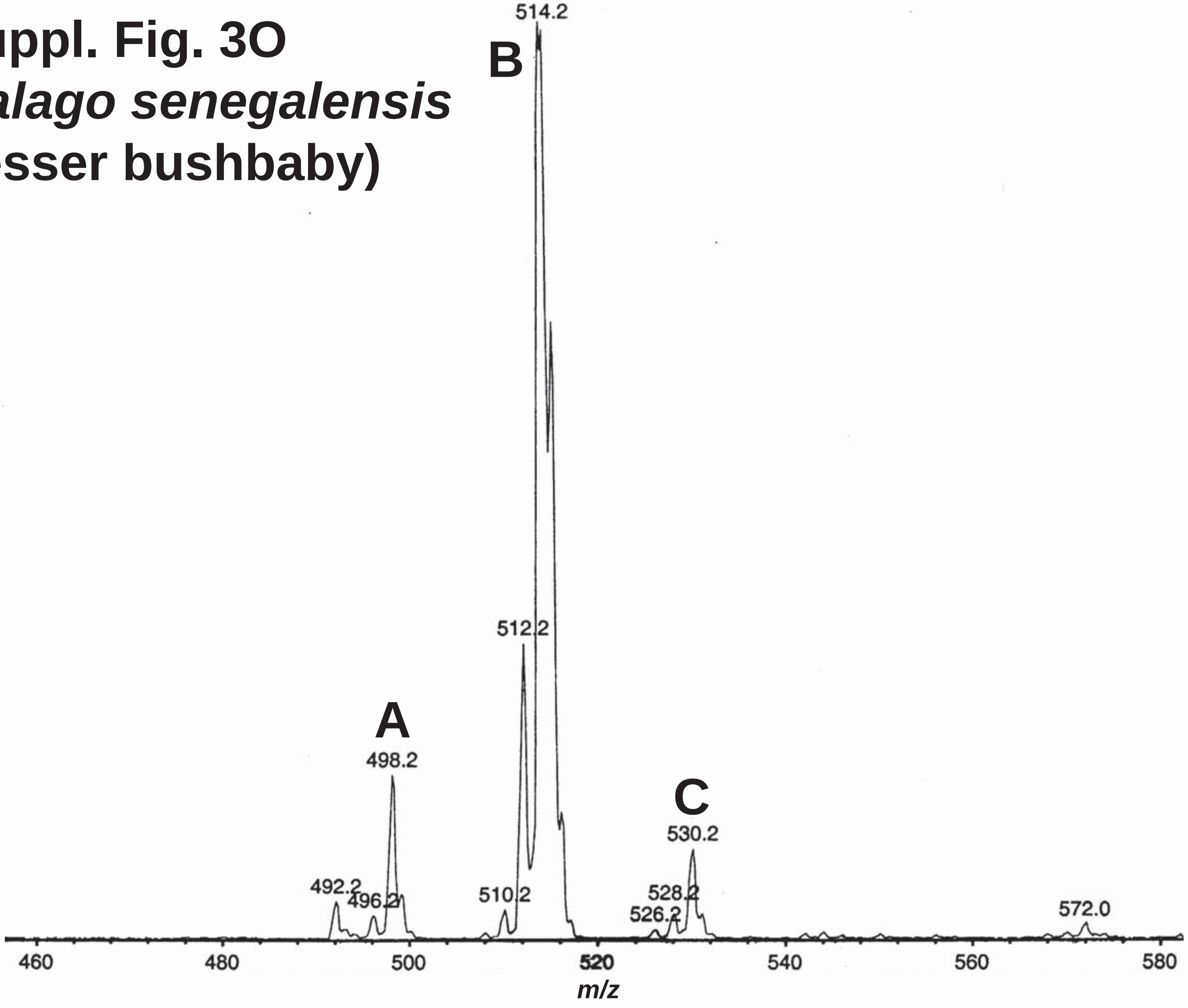

	Class	# of Hydroxyls	Conjugation
A[1]	C_{24} acid	2	Taurine
B[2]	C_{24} acid	3	Taurine
C	C_{24} acid	4	Taurine

[1] Taurochenodeoxycholic acid
[2] Taurocholic acid

Suppl. Fig. 3P
Galago senegalensis
(lesser bushbaby)

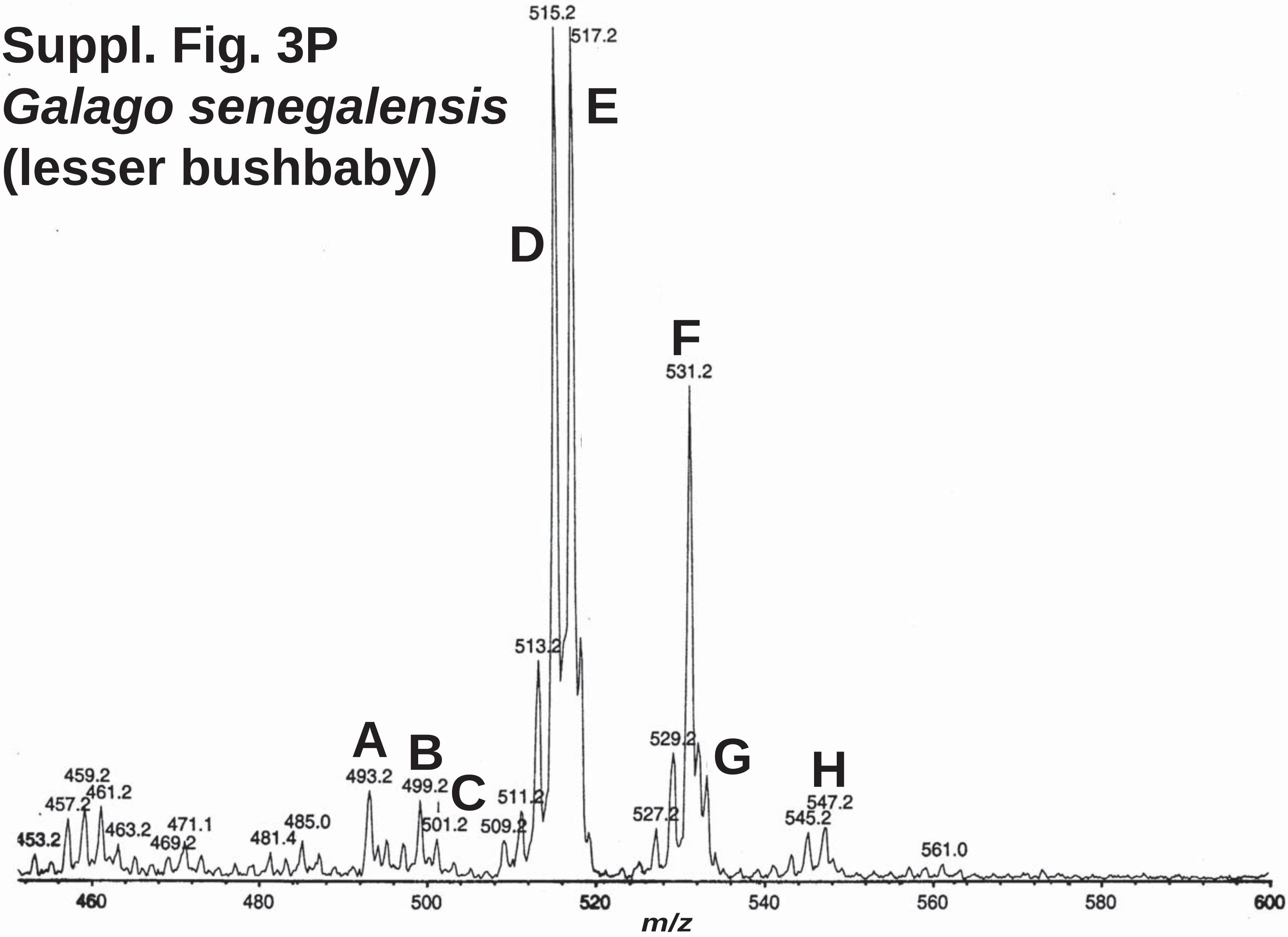

	Class	# of Hydroxyls	Conjugation
A	β-Sitosterol		Sulfate
B	C_{27} alcohol	3	Sulfate
C	C_{26} alcohol	4	Sulfate
D	C_{27} alcohol	4	Sulfate
E	C_{26} alcohol	5	Sulfate
F	C_{27} alcohol	5	Sulfate
G	C_{26} alcohol	6	Sulfate
H	C_{27} alcohol	6	Sulfate

Suppl. Fig. 3Q
Uromastyx acanthinura
(North African spiny-tailed lizard)

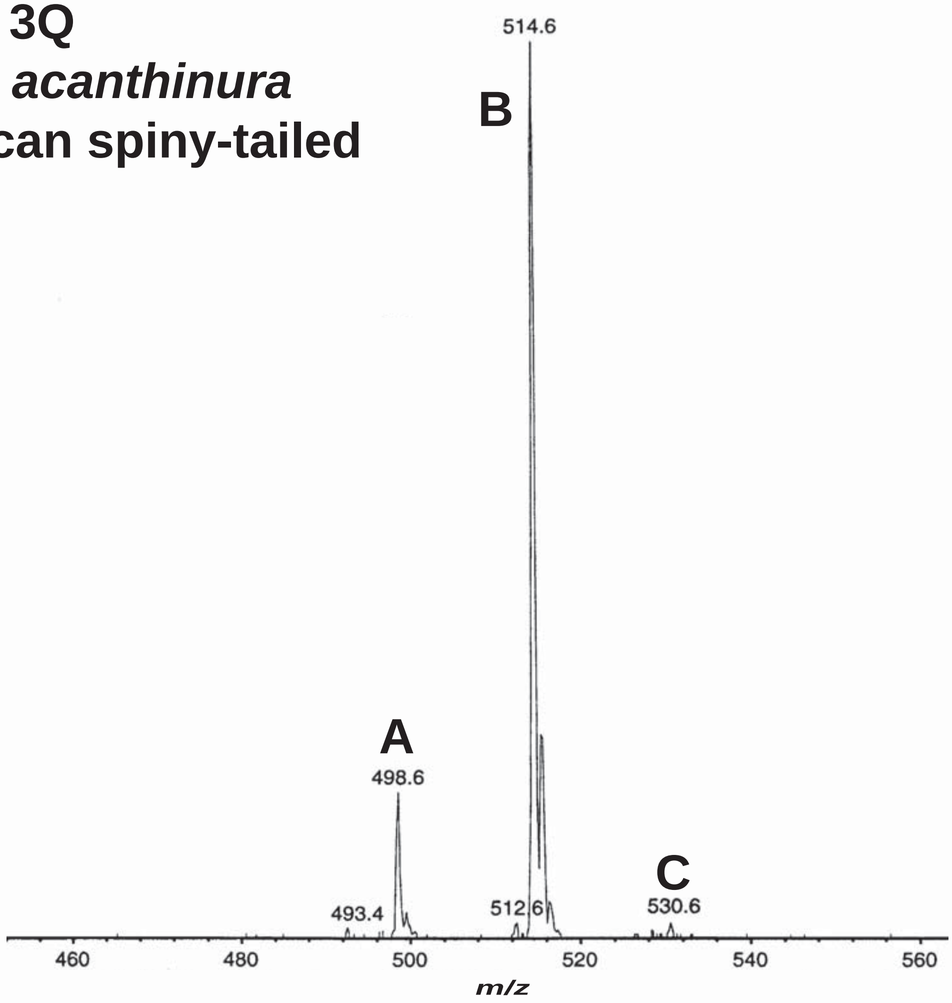

	Class	# of Hydroxyls	Conjugation
A[1]	C_{24} acid	2	Taurine
B[2]	C_{24} acid	3	Taurine
C	C_{24} acid	4	Taurine

[1] Tauroallodeoxycholic acid
[2] Tauroallocholic acid

Suppl. Fig. 3R
Uromastyx acanthinura (North African spiny-tailed lizard)

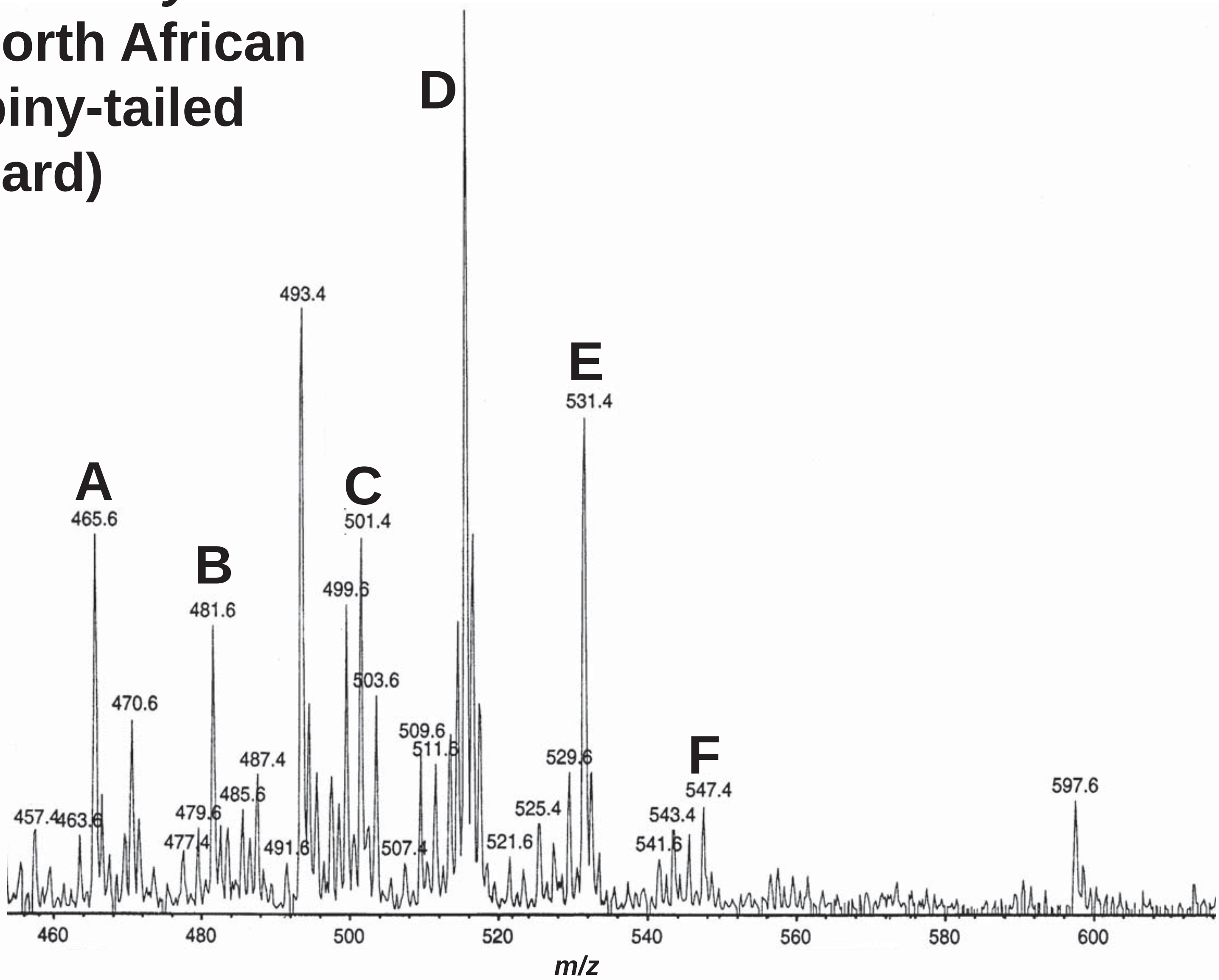

	Class	# of Hydroxyls	Double bonds (if any)	Conjugation
A	C_{27} alcohol	1	1	Sulfate
B	C_{27} alcohol	2	1	Sulfate
C	C_{27} alcohol	3		Sulfate
D	C_{27} alcohol	4		Sulfate
E	C_{27} alcohol	5		Sulfate
F	C_{27} alcohol	6		Sulfate

Suppl. Fig. 3S
Enhydris plumbea
(plumbeous water snake)

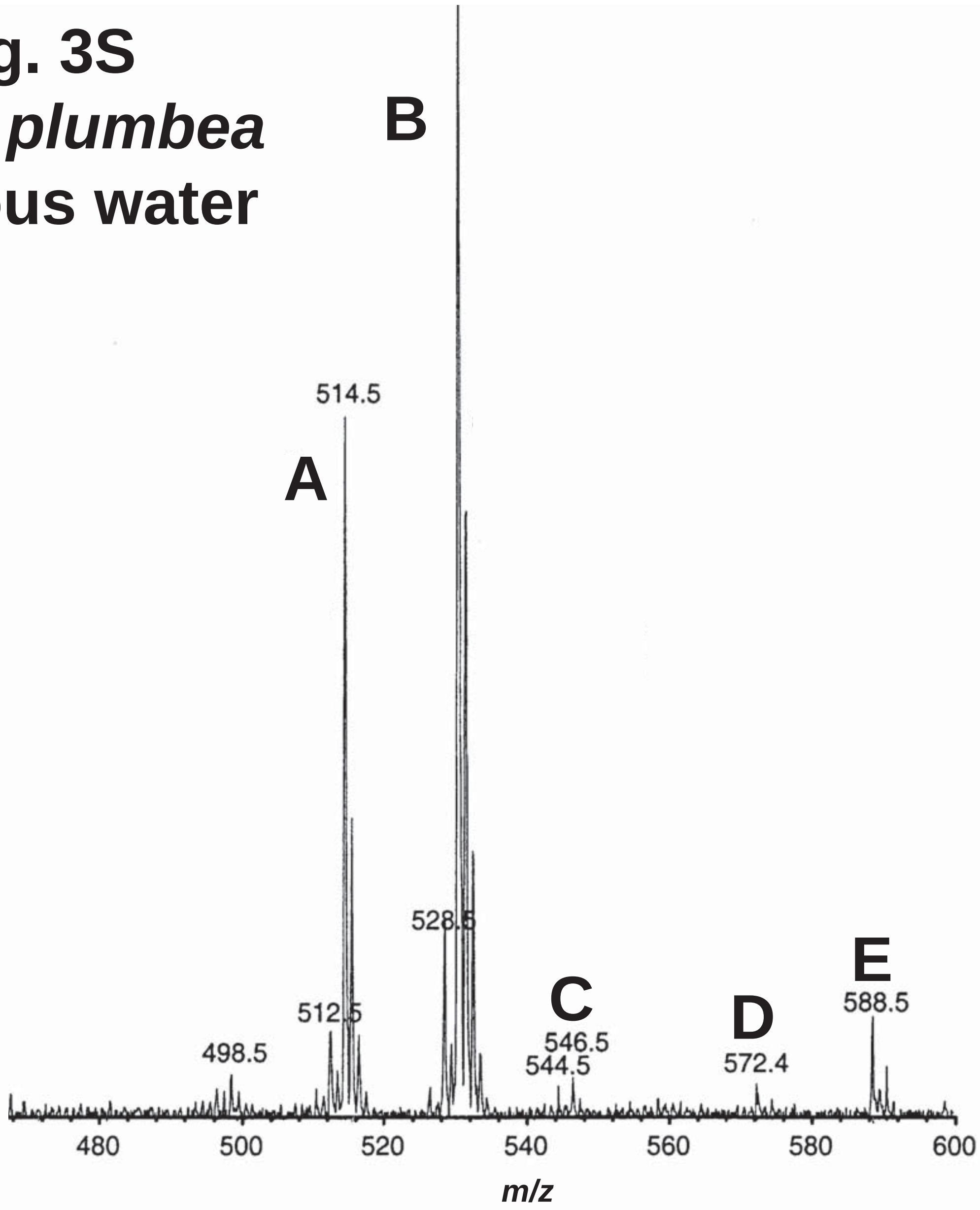

	Class	# of Hydroxyls	Conjugation	Adducts (if any)
A	C_{24} acid	3	Taurine	
B	C_{24} acid	4	Taurine	
C	C_{24} acid	5	Taurine	
D	C_{24} acid	3	Taurine	Na
E	C_{24} acid	4	Taurine	Na

Suppl. Fig. 3T
Enhydris plumbea
(plumbeous water snake)

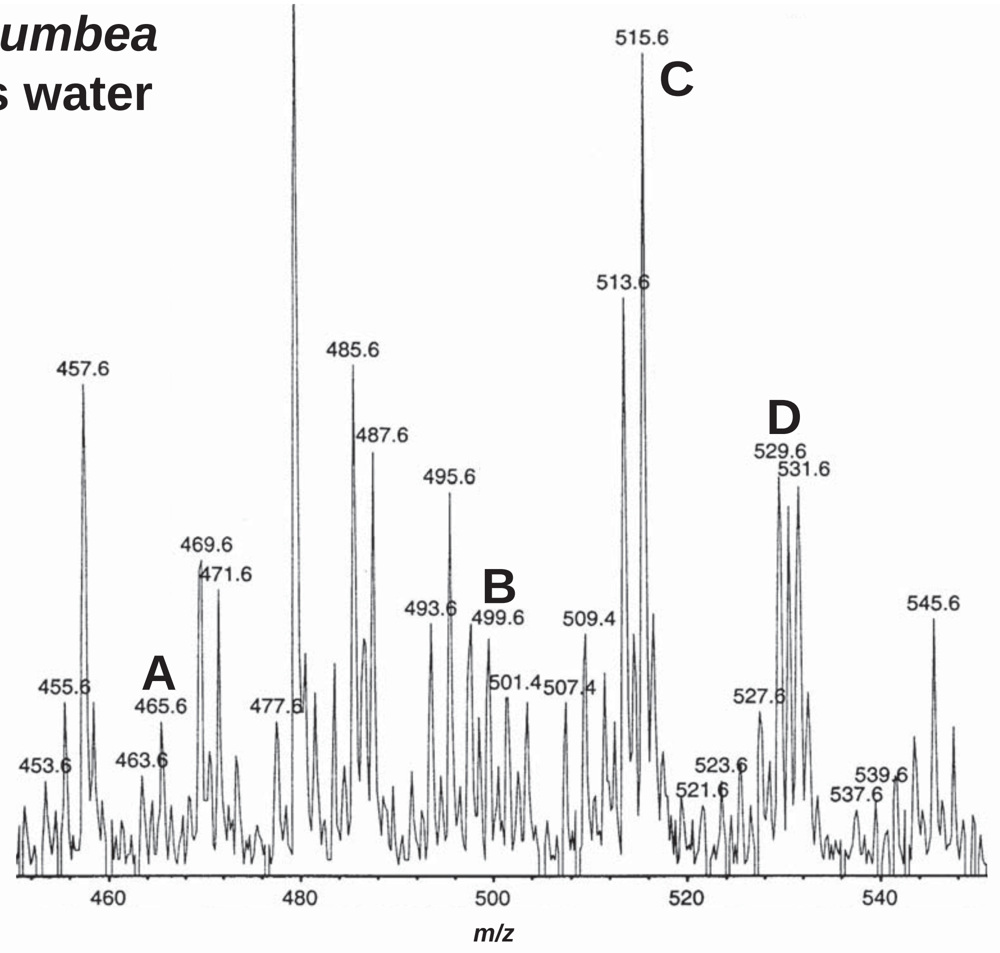

	Class	# of Hydroxyls	Double bonds (if any)	Conjugation
A	C_{27} alcohol	1	1	Sulfate
B	C_{27} alcohol	3		Sulfate
C	C_{27} alcohol	4		Sulfate
D	C_{27} alcohol	5		Sulfate

Suppl. Fig. 3U
Naja haje
(Egyptian banded cobra)

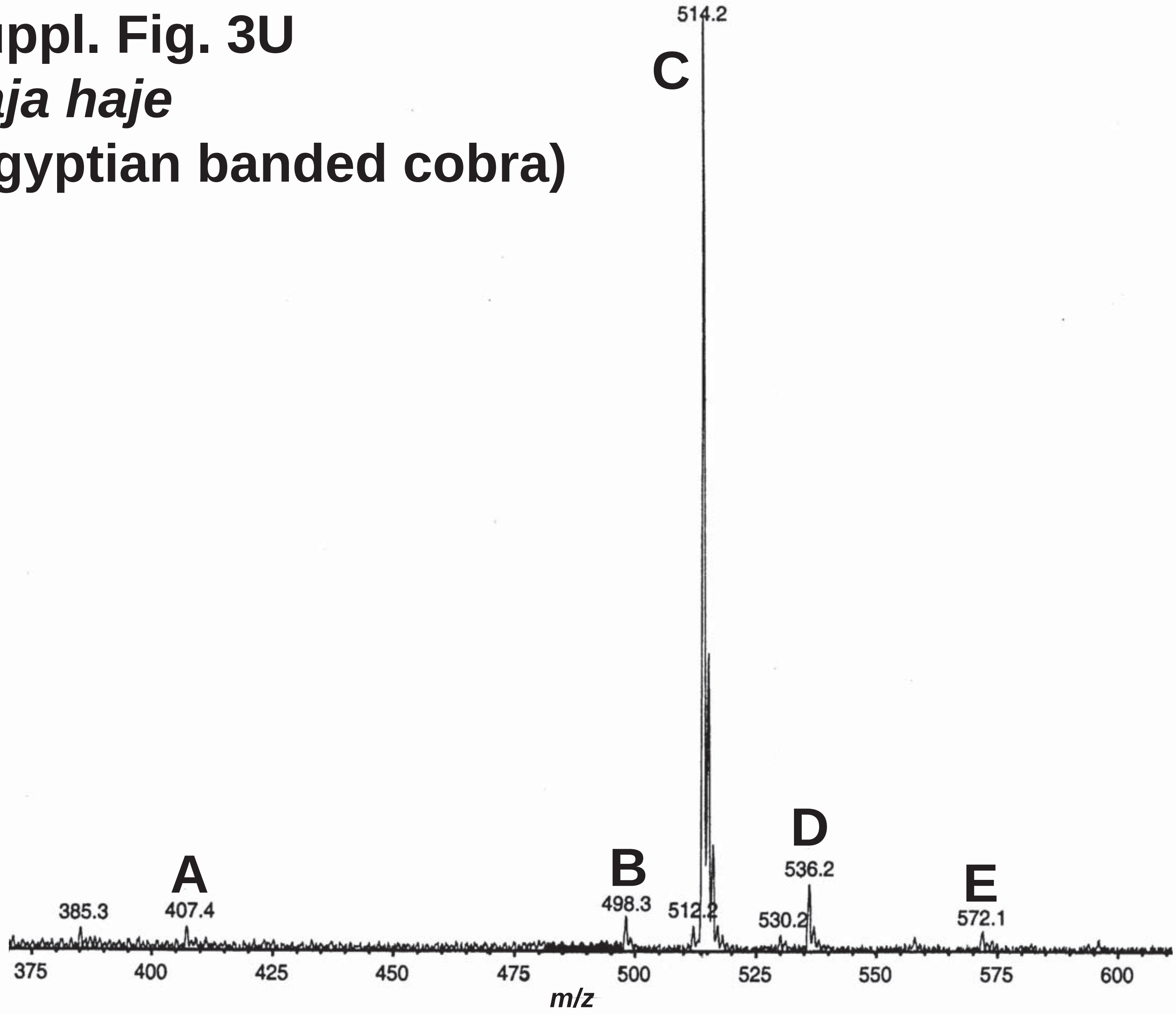

	Class	# of Hydroxyls	Conjugation (if any)	Adducts (if any)
A[1]	C_{24} acid	3		
B[2]	C_{24} acid	2	Taurine	
C[3]	C_{24} acid	3	Taurine	
D	C_{24} acid	3	Taurine	Na
E	C_{24} acid	3	Taurine	NaCl

[1] Cholic acid
[2] Taurochenodeoxycholic acid
[3] Taurocholic acid

Suppl. Fig. 3V
Tragulus javanicus
(lesser Malay chevrotain)

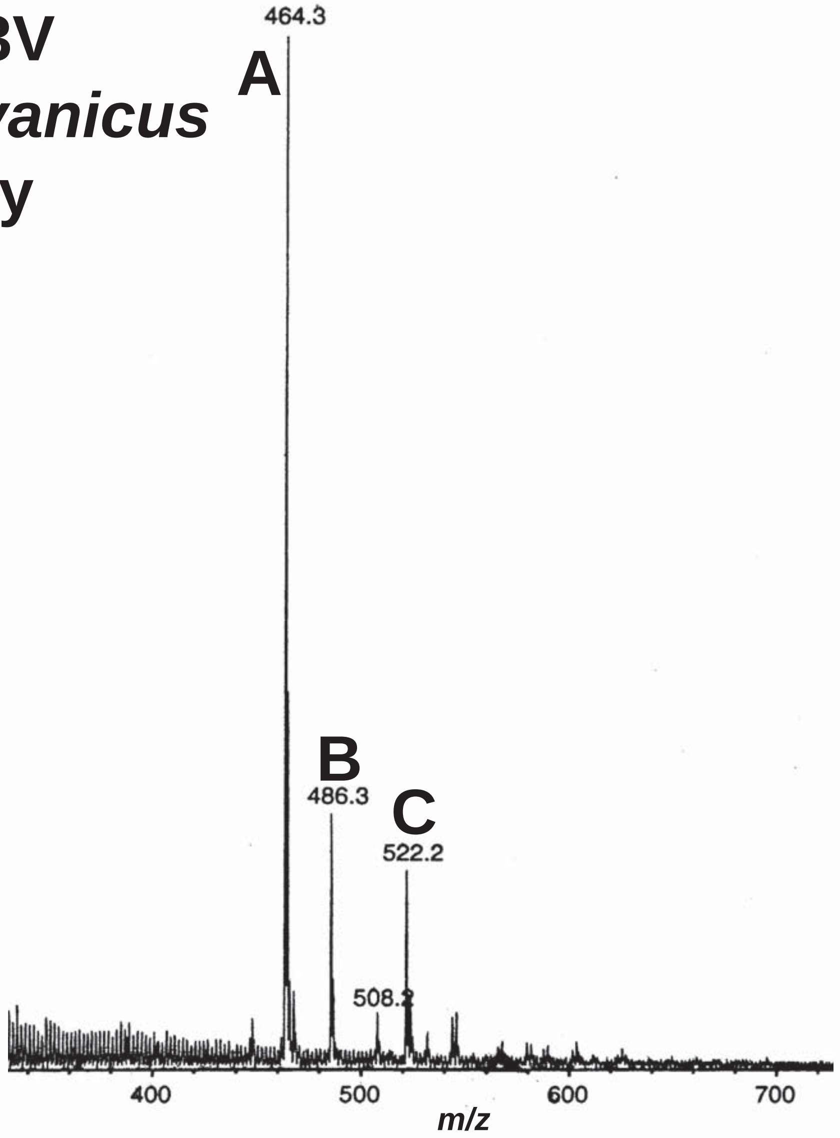

	Class	# of Hydroxyls	Conjugation	Adducts (if any)
A	C_{24} acid	3	Glycine	
B	C_{24} acid	3	Glycine	Na
C	C_{24} acid	3	Glycine	NaCl

Suppl. Fig. 3W
Tragulus javanicus
(lesser Malay chevrotain)

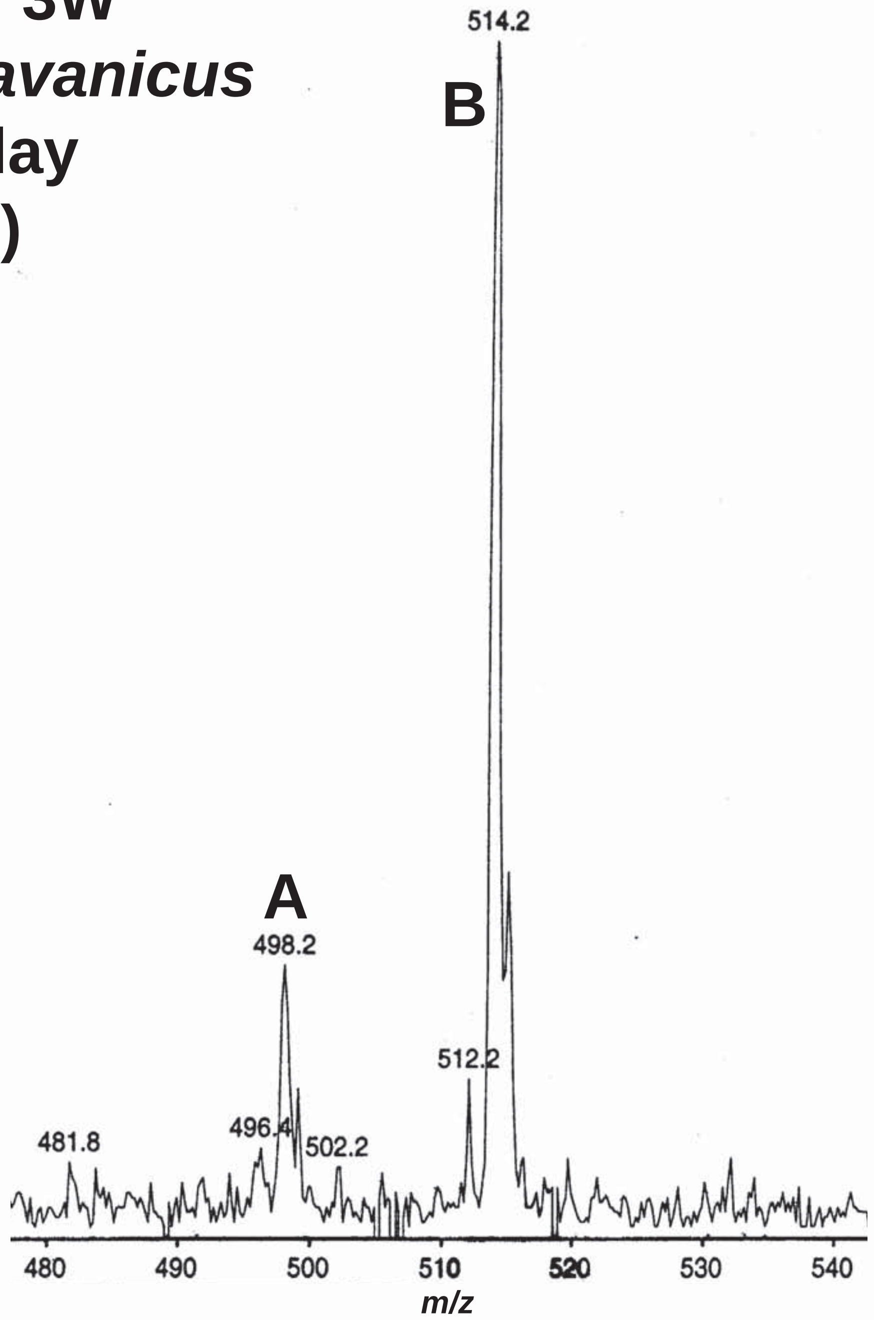

	Class	# of Hydroxyls	Conjugation
A*	C_{24} acid	2	Taurine
B	C_{24} acid	3	Taurine

* Taurochenodeoxycholic acid

Suppl. Fig. X *Tragulus javanicus* (lesser Malay chevrotain)

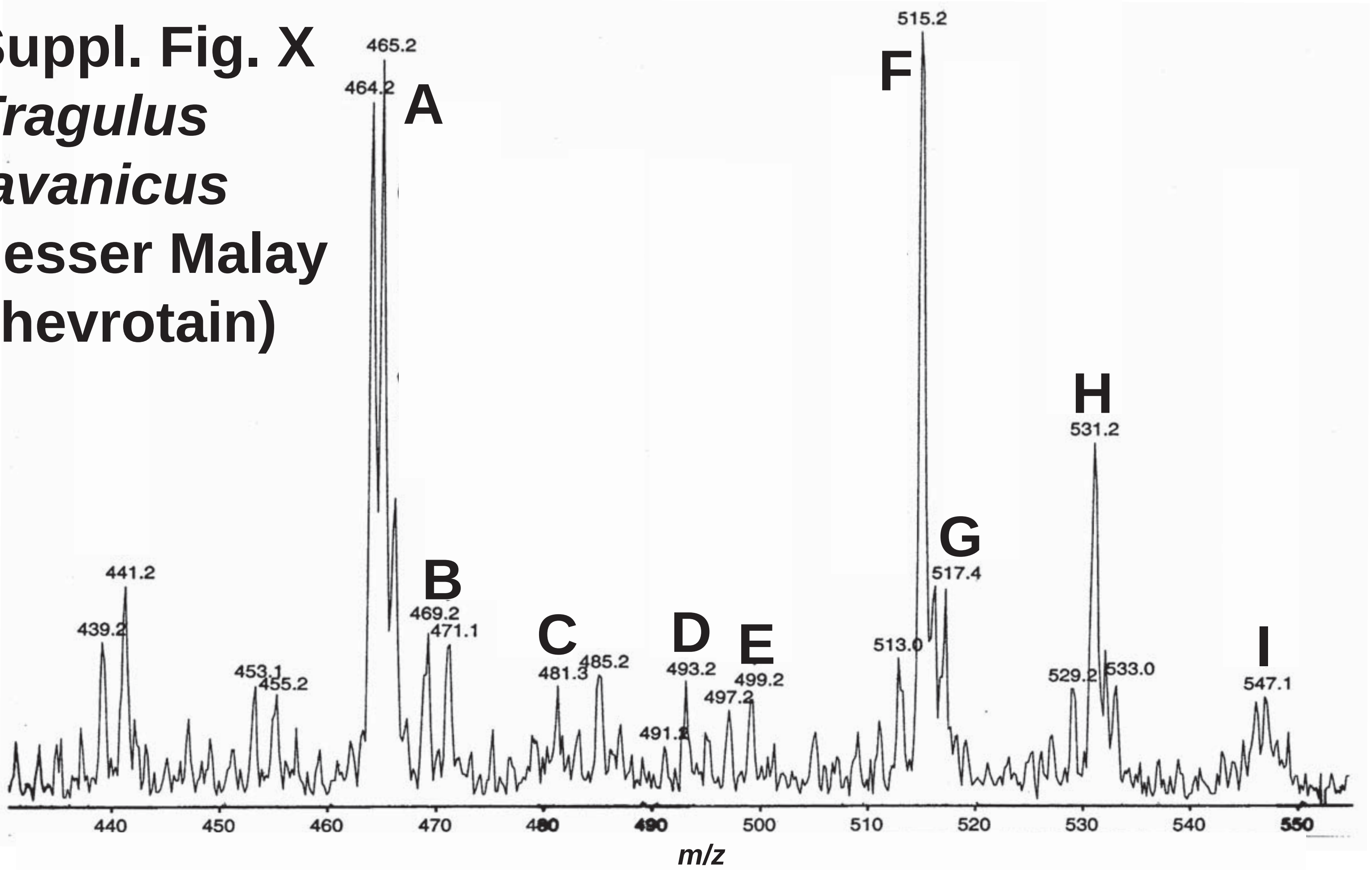

	Class	# of Hydroxyls	Double bonds (if any)	Conjugation
A	C_{27} alcohol	1	1	Sulfate
B	C_{24} acid	2		Sulfate
C	C_{27} alcohol	2	1	Sulfate
D	β-Sitosterol*			Sulfate
E	C_{27} alcohol	3		Sulfate
F	C_{27} alcohol	4		Sulfate
G	C_{26} alcohol	5		Sulfate
H	C_{27} alcohol	5		Sulfate
I	C_{27} alcohol	6		Sulfate

* For β-sitosterol, there were no extra hydroxyl groups in addition to the core structure.

Suppl. Fig. 3Y
Choloepus hoffmanni
(Hoffmann's two-toed sloth)
Feces

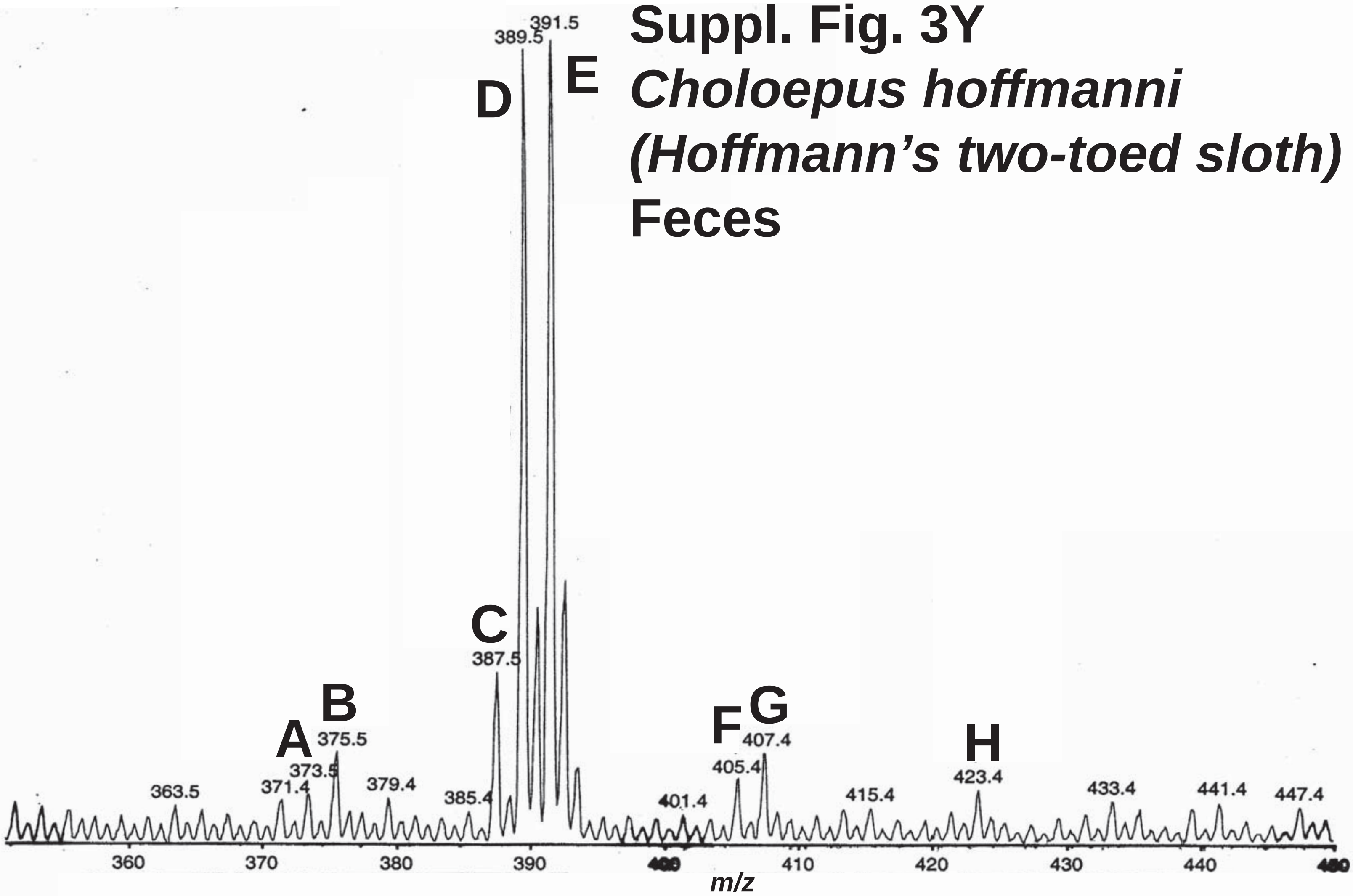

	Class	# of Hydroxyls	# of oxo groups	Double bonds (if any)
A	C_{24} acid	1		1
B[1]	C_{24} acid	1		
C	C_{24} acid	1	1	1
D	C_{24} acid	1	1	
E[2]	C_{24} acid	2		
F	C_{24} acid	2	1	
G	C_{24} acid	3		
H	C_{24} acid	4		

[1] Lithocholic acid
[2] Chenodeoxycholic acid

Suppl. Fig. 3Z
Choloepus hoffmanni
(Hoffmann's two-toed sloth)
Feces

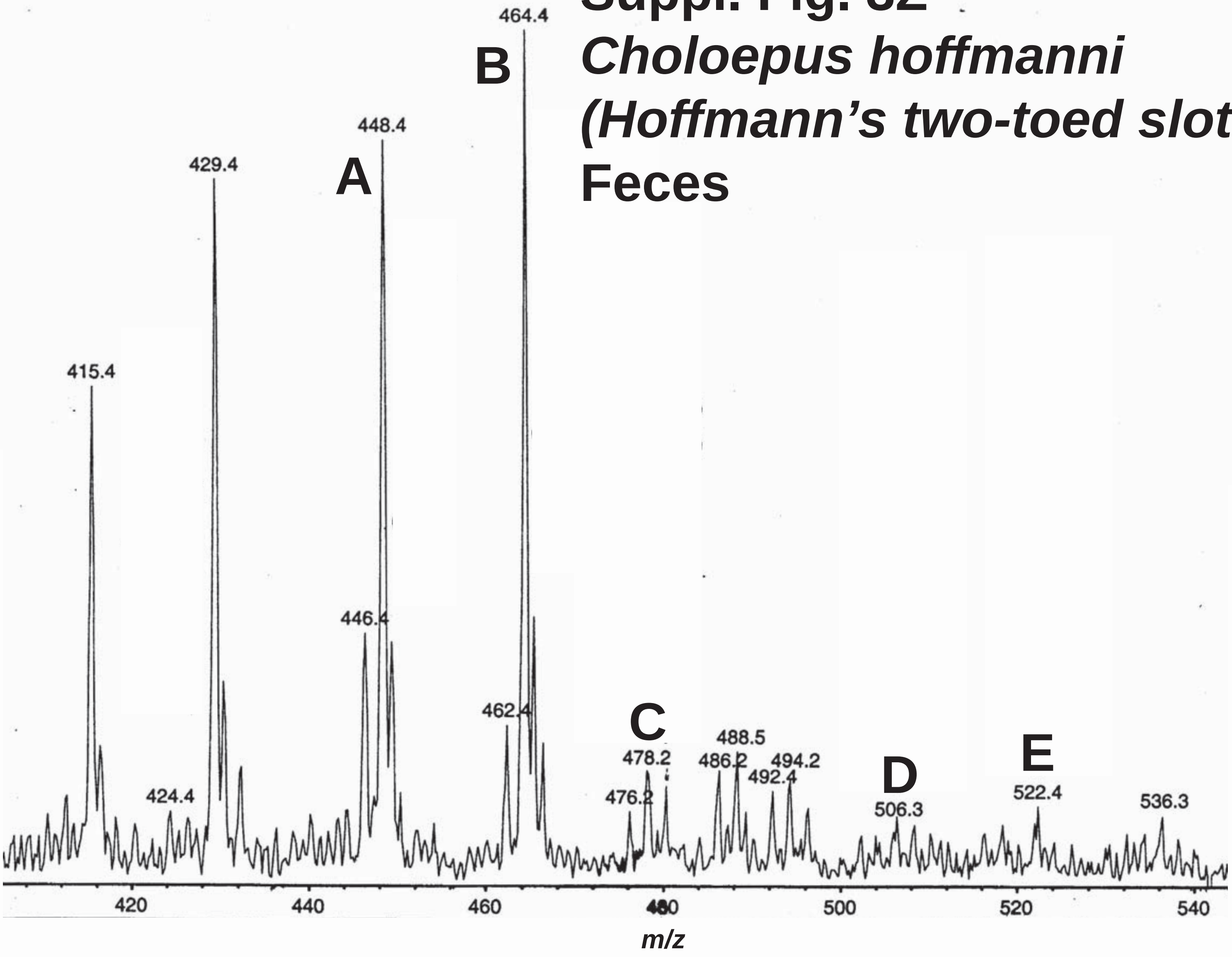

	Class	# of Hydroxyls	Conjugation (if any)
A	C_{24} acid	2	Glycine
B	C_{24} acid	3	Glycine
C	C_{24} acid	4	Glycine
D	C_{27} acid	3	Glycine
E	C_{27} acid	4	Glycine

Suppl. Fig. 3AA
Choloepus hoffmanni
(Hoffmann's two-toed sloth)
Feces

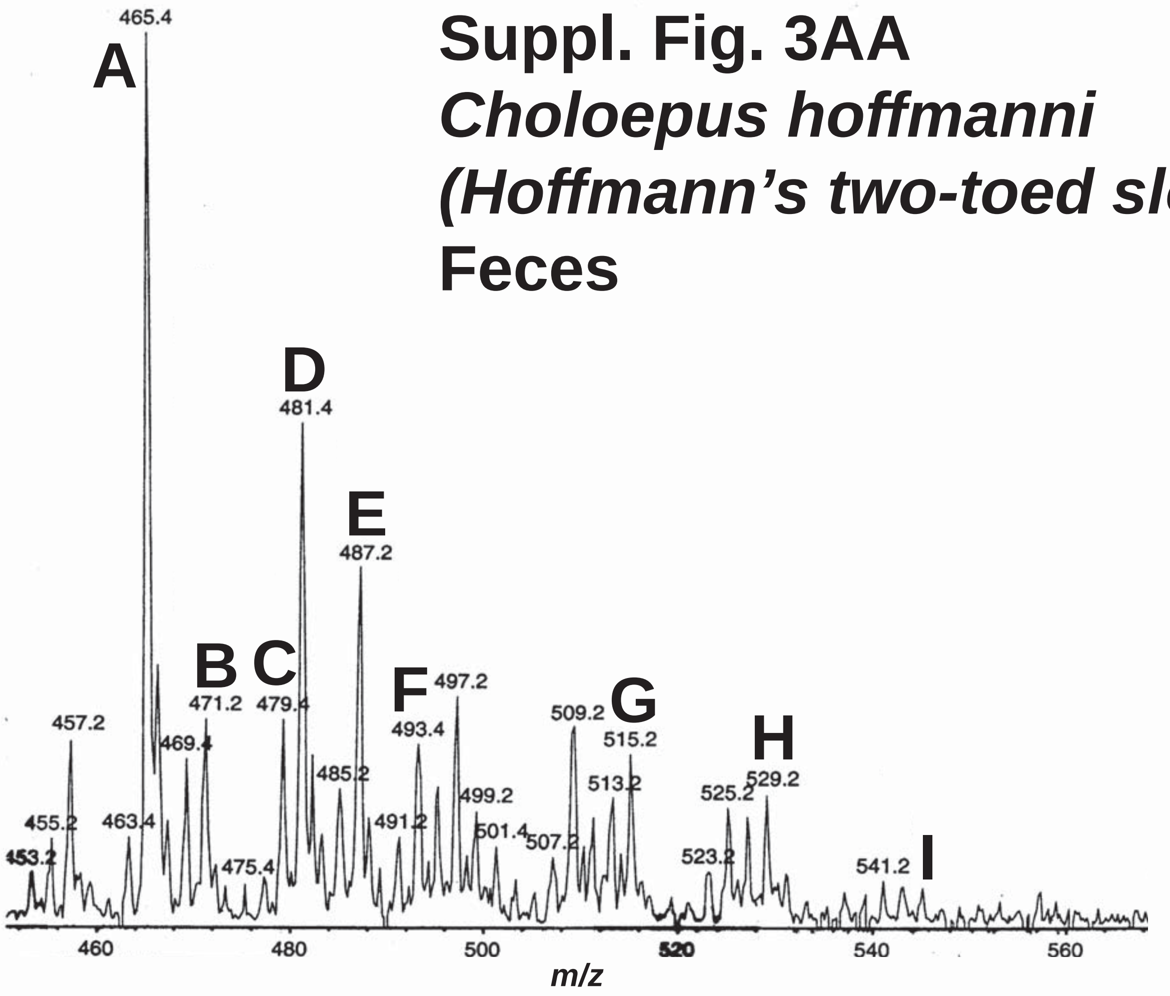

	Class	# of Hydroxyls*	Double bonds (if any)	Conjugation
A	C_{27} alcohol	1	1	Sulfate
B	C_{24} acid	2		Sulfate
C	Campesterol			Sulfate
D	C_{27} alcohol	2	1	Sulfate
E	C_{24} acid	2		Sulfate
F	β-Sitosterol			Sulfate
G	C_{27} alcohol	4		Sulfate
H	C_{27} acid	3		Sulfate
I	C_{27} acid	4		Sulfate

* For the plant sterols, there were no extra hydroxyl groups in addition to the core structure

Suppl. Fig. 3BB
Homo sapiens
(human) - 8,000 year old coprolite

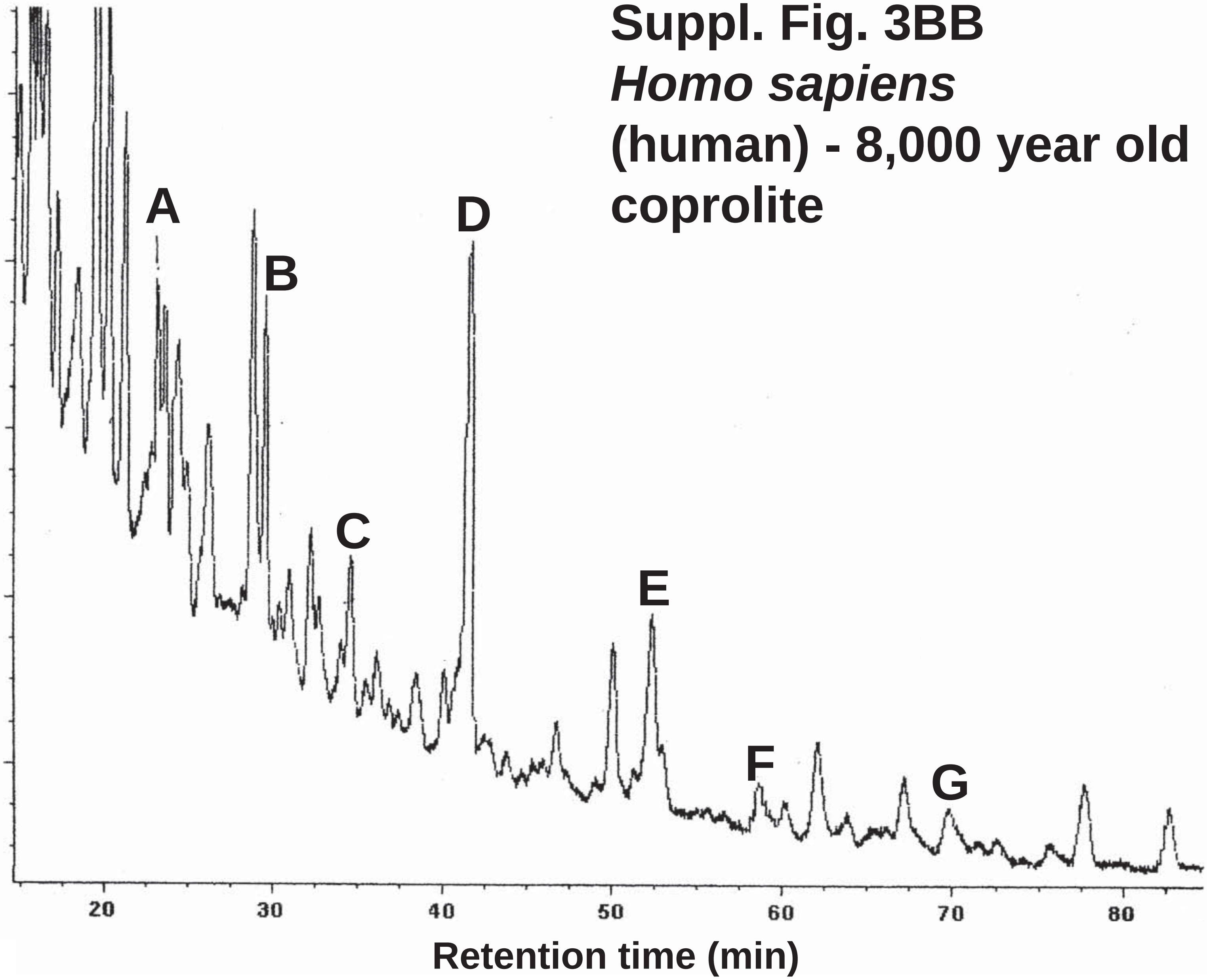

A: 3β-Hydroxy-5α-cholestane
B: Lithocholic acid
C: Stigmastanol
D: Deoxycholic acid
E: 12-Ketolithocholic acid
F: Cholic acid
G: 7-Ketodeoxycholic acid

Suppl. Fig. 3CC
Nothrotherium shastense (Shasta ground sloth) coprolite

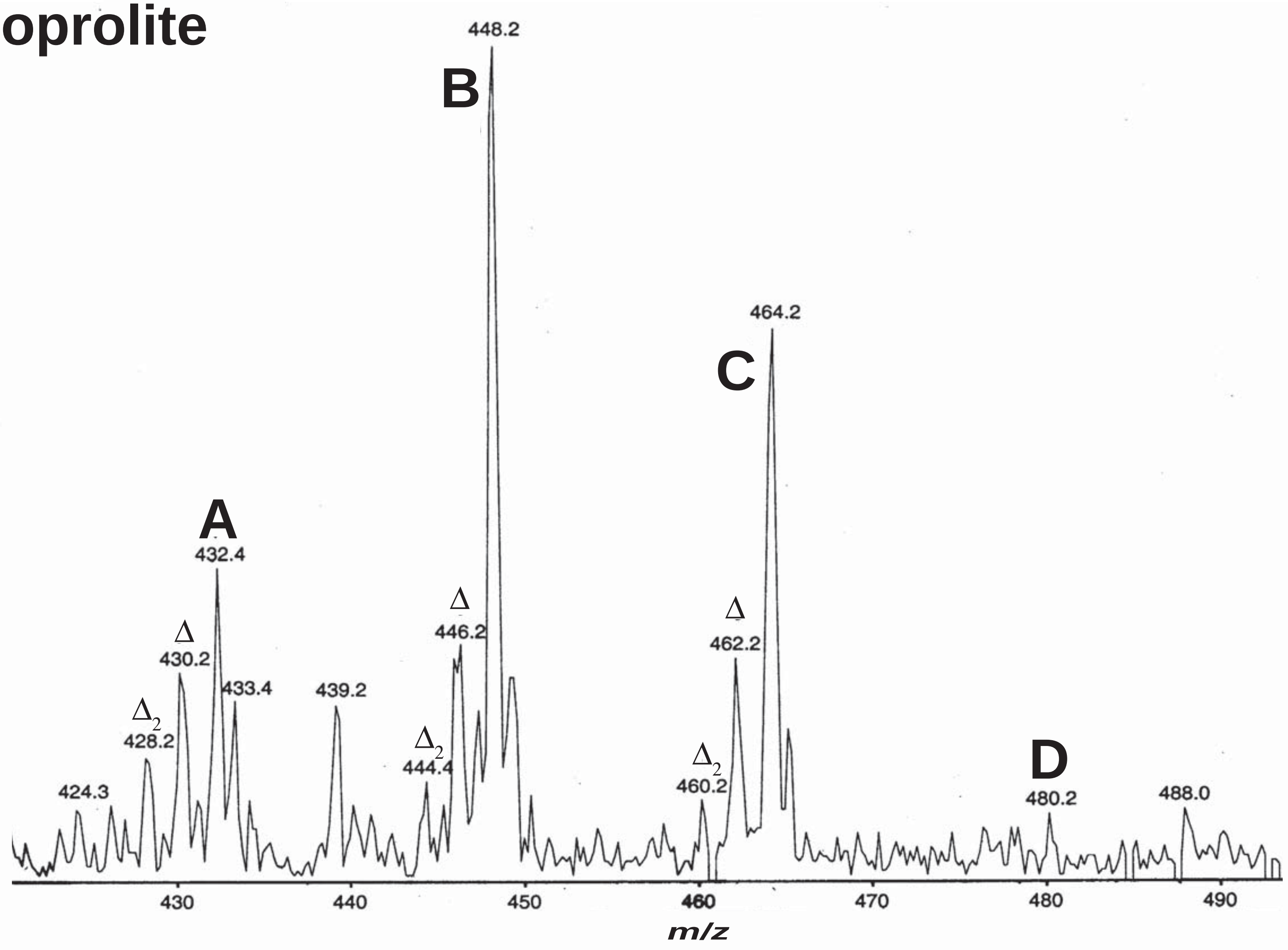

	Class	# of Hydroxyls	Conjugation
A[1,*]	C_{24} acid	1	Glycine
B[*]	C_{24} acid	2	Glycine
C[*]	C_{24} acid	3	Glycine
D	C_{24} acid	4	Glycine

[1] Glycolithocholic acid

* Peaks with *m/z* ratios consistent with compounds A, B, and C having one (Δ) or two (Δ_2) double bonds are indicated

Suppl. Fig. 3DD
Nothrotherium shastense (Shasta ground sloth) coprolite

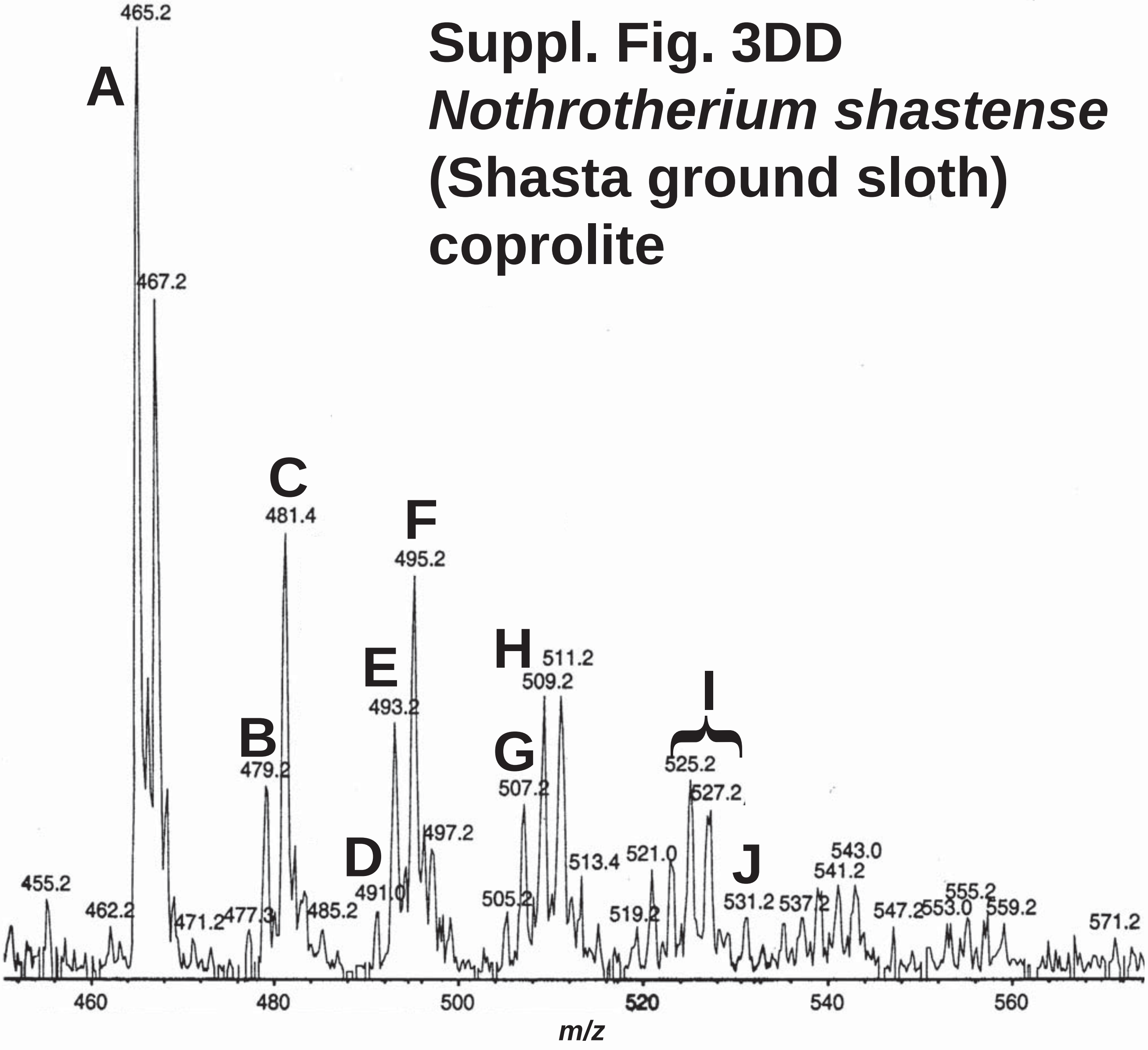

	Class	# of Hydroxyls[1]	Double bonds (if any)	Conjugation
A	C_{27} alcohol	1	1	Sulfate
B	Campesterol			Sulfate
C	C_{27} alcohol	2	1	Sulfate
D	Stigmasterol			Sulfate
E	β-Sitosterol			Sulfate
F	Stigmastanol			Sulfate
G	β-Sitosterol	2		
H	Stigmastanol	2		
I[2]	Additional plant sterols?	3?		
J	C_{27} alcohol	5		

[1] For the plant sterols, # of hydroxyls indicates hydroxyl groups in addition to the core structure

[2] The peaks at m/z = 525.2 and 527.2 are consistent with trihydroxy plant sterols
