# Supplementary material for: Evolutionary diversity of bile salts in reptiles and mammals, including analysis of ancient human and extinct giant ground sloth coprolites
Source: BMC Evol Biol. 2010 May 6;10:133. doi: 10.1186/1471-2148-10-133 (PMC2886068; doi:10.1186/1471-2148-10-133)
Supplement: Additional file 5 — Model animals that may provide key insight into bile salt biosynthesis. Table compares and contrasts differences of bile salt enzymes between 5 model species that have interesting bile salt profiles. [file 1471-2148-10-133-S5.PDF]

Additional file 5
Model animals that may provide key insight into bile salt biosynthesis

**Animal**	**Bile salt profile**	**CYP27A1 ortholog?**	**AKR1D1 ortholog?**	**Key enzymes for future studies**
Sea lamprey (*Petromyzon marinus*)	I (C_{24} and C_{27} 5α-bile alcohols)	Unknown	Unknown	Enzyme(s) involved in side-chain shortening<br>Enzymes involved in 5α-reduction
Zebrafish (*Danio rerio*)	I (C_{27} 5α-bile alcohols)	Yes	**No**	CYP27A1<br>Enzymes that mediate 5α-reduction
Green anole lizard (*Anolis caroliensis*)	VI (C_{24} 5α-bile acids)	Yes	Yes	CYP27A1<br>AKR1D1 (does it mediate 5α-reduction?)
African elephant (*Loxodonta africana*)	I (C_{27} 5β-bile alcohols)	Yes	Yes	CYP27A1, other peroxisomal enzymes
Rock hyrax (*Procavia capensis*)	I (C_{27} 5β-bile alcohols)	Yes	Yes	CYP27A1, other peroxisomal enzymes
